# Mathematical strategies for predicting resistant subpopulations from scRNAseq data of a PANC-1 3D tissue model: Insight into gemcitabine resistance and TGFB1-induced invasion and EMT

**DOI:** 10.1016/j.csbj.2025.10.032

**Published:** 2025-10-16

**Authors:** Aylin Caliskan, Samantha A.W. Crouch, Jesús Guillermo Nieves Pereira, Thomas Dandekar, Gudrun Dandekar, Tim Breitenbach

**Affiliations:** a Department of Bioinformatics, Biocenter, University of Würzburg, Am Hubland, Würzburg 97074, Germany; b Department for Functional Materials in Medicine and Dentistry, University Hospital of Würzburg, Röntgenring 11, Würzburg 97070, Germany; c Institute for Molecular Medicine, Medical Faculty, Martin Luther University Halle-Wittenberg, Kurt-Mothes-Straße 3a, Halle (Saale) 06120, Germany

**Keywords:** Pseudobulk RNAseq analysis, 3D tissue model, Pancreatic cancer, EMT, gemcitabine, TGFB1, resistance

## Abstract

**Background:**

Pancreatic ductal adenocarcinoma is characterized by high levels of chemoresistance and aggressive progression of the disease, which is a major challenge for effective treatment. Conventional 2D cultures capture therapy resistance only to a limited extent, whereas 3D cultures may better reflect relevant conditions.

**Methods:**

We established 3D PANC-1 tissue models based on a decellularized porcine jejunum with niche-specific drug response to gemcitabine (GEM) treatment. TGFB1 induced invasion and further drug resistance. Thus, we performed scRNA-seq after treatment with GEM, TGFB1 stimulation, or both. Data were analyzed using standard approaches and a novel mutual-information-based machine learning framework (gSELECT). Candidate genes were further evaluated through enrichment and survival analyses.

Additionally, we present a novel mathematical approach as proof of concept for analyzing differences in gene expression between groups seemingly similar with respect to a projection such as t-SNE or UMAP (e.g., GEM-treated and untreated cells). For this, we stratified control cells by similarity to GEM-treated survivors, yielding predicted-resistant and predicted-sensitive subgroups for downstream analysis.

**Results:**

Pre-analysis using machine learning and comparative analyses of single-cell RNA sequencing data showed only minor differences in gene expression in response to GEM treatment, whereas TGFB1 induced an invasive phenotype characterized by EMT-related transcriptional changes, including downregulation of cytokeratins.

Laboratory experiments showed that ∼75 % of PANC-1 cells survived GEM in 3D, indicating intrinsic resistance. Our mathematical approach using machine learning predicted a GEM-sensitive subpopulation consistent with these findings. Comparative analyses revealed mutual information (MI) genes distinguishing sensitive from resistant cells, several of which were supported by literature and survival data.

**Conclusion:**

Computational analysis of scRNA-seq data from 3D-cultured PANC-1 cells provides a useful framework for studying treatment effects. The potential relevance of the identified MI genes is supported by further *in silico* analyses. Based on *in silico* analyses, we demonstrate the analytical value of our mathematical approach and identify candidate genes for further functional and therapeutic validation.

## Graphical Abstract

**Figure:**
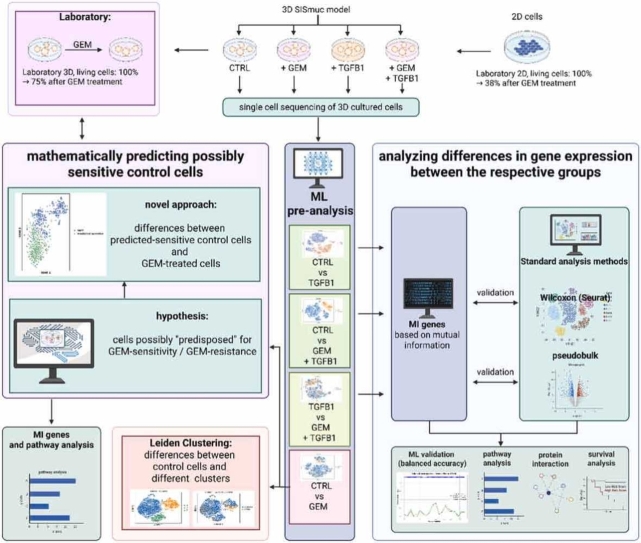

## 1 Introduction

Pancreatic cancer includes several malignancies of the pancreas, with pancreatic ductal adenocarcinoma (PDAC) being the most common and aggressive form, accounting for more than 90 % of clinical cases [1], [2]. PDAC is ranked sixth among cancer-related deaths worldwide [3], and is responsible for 7–8 % of all cancer deaths in the United States, where it is the fourth most deadly cancer type [3], [4]. Due to its increasing incidence, it is predicted to be the second most deadly cancer in Western societies by 2030 [5]. With most of the PDAC cases being incurable at diagnosis and a 5-year survival rate of approximately 11 %, the prognosis for PDAC remains poor [6]. The reasons behind this general and poor outcome could be related to its lack of specific symptoms and markers in the initial stages [7]. This directly and negatively affects the chances of early detection, which instead happens at later stages when there is already some degree of local or distal metastasis [7]. Moreover, PDAC mortality is further worsened by the commonly poor response to first-line treatments such as the chemotherapies FOLFIRINOX and gemcitabine (GEM) [8], [9], [10], which have been reported to result in a median overall survival of approximately 24 months and 6–13 months, respectively [8], [9], [10]. Taken together, this set of molecular and clinical features makes PDAC one of the most aggressive malignancies with the poorest prognosis, leading the list of the lowest 5-year survival rates among cancers, with 12.8 % in the US [3].

Traditional 2D cell culture models have demonstrated limited translational potential in PDAC research, as they fail to replicate the complex tumor microenvironment and cellular interactions observed *in vivo* [11], [12], [13]. For instance, they lack the desmoplastic reaction, a hallmark of PDAC characterized by a dense extracellular matrix (ECM) populated with activated stromal cells, which constitutes approximately 60–90 % of the tumor mass [14], [15], [16]. To address these limitations, various 3D culture systems have been developed to better mimic tumor behavior [17], [18]. In this study, the Small Intestine Submucosa and mucosa (SISmuc) matrix, rich in different collagens and fibronectin, was utilized as a scaffold in the development of a novel 3D model, providing key ECM components and tissue architecture that better simulate the desmoplastic reaction. As a surrogate for tumor-driving factors from stromal cells, TGFB1 was added. This system not only supports essential cell-ECM interactions but also facilitates a more realistic tumor morphology, including a basement membrane structure and a tissue-like homoeostasis with *in vivo*-like proliferation indices as demonstrated in models for lung cancer [19], [20] and colorectal cancer [21]. The PANC-1 cell line was selected as a representative for PDAC cells due to its characteristic mutations in *KRAS*, *P53*, and *P16/CDKN2A* [22]. In comparison to Capan-1 cells, PANC-1 cells showed a more malignant phenotype on our tissue matrix, with functional invasive tumor cells crossing the preserved basement membrane [23].

This 3D model provides a platform to study TGFB1-driven EMT, a key mechanism in tumor growth and metastasis. Moreover, the high survival rate of GEM-treated cells under 3D conditions reflects the limited clinical efficacy of GEM in patients. We therefore performed single-cell RNA sequencing of a 3D PANC-1 SISmuc model after GEM treatment, TGFB1 stimulation, or both, and analyzed the data using machine learning and mathematical approaches.

Specifically, we applied the tool gSELECT, which ranks genes by mutual information (MI genes) between cells with different phenotypic characteristics, such as treated vs. untreated or resistant vs. responsive, and uses these features in a classifier to assess subgroup separability [24], [25], [26]. High classification accuracy indicates sufficient discriminatory information, whereas low accuracy might reflect strong similarity between groups. Thus, gSELECT pre-analysis can help determine whether further subgroup comparisons are likely to be informative. When comparing treated and untreated cells, such as control cells and GEM-treated cells, a high similarity (and consequently, a low prediction accuracy) between the two groups can also indicate possible treatment resistance, in particular when there is a big subgroup within the control cells that is resistant and might not be affected by the treatment.

Since most single-cell RNA sequencing protocols require intact and viable cells [27], cells obtained from a treatment group (e.g., GEM-treated cells) have likely survived the respective treatment and are thus likely to be less affected or potentially even resistant to the treatment. Cells affected by the treatment are not sequenced. To address this inherent limitation of single-cell sequencing, we introduce a novel mathematical approach that enables the retrospective inference of treatment susceptibility within untreated cell populations. Based on the hypothesis that untreated cells with high similarities to cells surviving a certain treatment are likely to survive the treatment (predicted-resistant), we assume that cells with little similarity to treated cells are more likely to be affected by the respective treatment (predicted-sensitive). This classification aims to overcome the bias in single-cell sequencing that results from profiling only surviving cells, enabling focused downstream analyses, including MI-based gene ranking and pathway enrichment. Differences in gene expression between the subpopulation of untreated cells that are predicted-sensitive and the treated cells might identify genes involved in treatment response and could support hypothesis generation.

In the following, we analyze the sequencing data of our 3D pancreatic cancer model using a machine-learning-based pre-analysis method (gSELECT [26]) to identify potential genes of interest based on MI. Subsequently, we will introduce a mathematical approach to predict sensitivity to treatment in untreated cells. By comparing these predicted-sensitive cells to cells after treatment, in our example, GEM-treatment, we obtain potential genes of interest based on mutual information. These MI genes might be involved in the treatment response and could therefore be promising targets for future research.

## 2 Methods

### 2.1 Biology-related methods

#### 2.1.1 2D cell culture

2D cell culture was performed under standard conditions (37 °C, 5 % CO_2_). PANC-1 cells (DSMZ, ACC-783) were cultured with Dulbecco’s Modified Eagle Medium (DMEM), GlutaMAX™ high glucose (Gibco, 61965059, Germany) supplemented with 1 mM sodium pyruvate (Invitrogen, P2256, Germany) and 10 % fetal calf serum (FCS, PAN Biotech, P303306, Germany) on a T75 or T150 cm^2^ cell culture flasks (Techno Plastic Products (TPP), 90076/90151, Switzerland) until 80–90 % confluency.

Subcultivation of PANC-1 cells was performed by adding 3–6 mL of 1 % trypsin diluted in PBS without calcium and magnesium ions (PBS-, Sigma-Aldrich, D8537–, Germany) supplemented with 0.5 mM ethylenediaminetetraacetic acid (EDTA, Sigma-Aldrich, E5134, Germany) to a T75 or T150 cell culture flask, respectively, and incubated for 3 min. The reaction was stopped either by the addition of twice the amount of FCS-supplemented specific cell medium or 2 mL FCS. The resulting cell suspension was then transferred to a 50 mL centrifuge tube (Greiner Bio-One, 188271 N, Germany) and spun down at 300 g for 5 min (Thermo Fisher Scientific, Multifuge X12, Germany). Afterwards, the supernatant was completely removed with a disposable pasteur pipette attached to a vacuum pump (KNF Neuberger, Germany), and the cell pellet was gently resuspended to the desired volume with the help of plastic serological pipettes of 5 or 10 mL (Greiner Bio-One, 606180/607180, Germany) with the specific cell medium.

#### 2.1.2 3D model generation and cell culture

Explanations of porcine jejunum followed the German Animal Protection Laws (§4 Abs. 3) and all animals received humane care in compliance with the guidelines by the FELASA, WHO and FDA (WHO-TRS978 Annex3 and FDA-OCTGT Preclinical Guidance) after approval from the institutional animal protection board (registration reference number #2532–2–12, Ethics Committee of the District of Unterfranken, Würzburg, Germany). Porcine jejunum was decellularized as described previously [28]. In short, after rinsing the intestinal porcine jejunum, the segments were decellularized chemically with sodium deoxycholate monohydrate solution and underwent extensive rinsing, followed by γ-sterilization. Inside a sterile workbench, the SISmuc scaffold was prepared by making an incision using a scalpel handle (Bayha, 504, Germany) with a blade (Bayha, N22, Germany). The scaffold was then sectioned into approximately 150 × 150 mm squares. Using sterile forceps (Bochem Laborbedarf, 1023, Germany), the cut pieces were positioned between two sterile ring-like structures, commonly referred to as “cell crowns”. These assembled SISmuc constructs were subsequently transferred to a sterile 12-well plate (TPP, 92012, Switzerland). To prevent dehydration and potential structural damage, 2.5 mL of specific cell culture medium was added to each well in a transwell-like configuration. The distribution of the medium was as follows: 1.5 mL was pipetted into the space between the well of the 12-well plate and the assembled cell crown (outer compartment), while 1 mL was pipetted into the inner ring (inner compartment). It is important to note that the luminal side of the former intestinal tissue always faced upwards, corresponding to the inner compartment of the model. SISmuc 3D models were established by seeding a pre-prepared cell suspension (2D culture) containing 2 × 10^5^ PANC-1 cells per milliliter of DMEM with 10 % FCS. From this suspension, 500 μL was carefully pipetted onto the luminal side of each transwell-like construct using disposable serological pipettes. The constructs were then incubated for 1 h, after which 1 mL of specific cell medium was added to both the luminal and basolateral compartments.

For routine medium exchange, the old medium was removed from both compartments using disposable glass Pasteur pipettes, followed by replenishment with 1 mL and 1.5 mL of fresh medium in the inner and outer compartments, respectively. This procedure was performed at each medium change.

#### 2.1.3 3D models stimulation and treatment

Gemcitabine (Sigma-Aldrich, G6423–10MG, Germany) was dissolved in sterile DMEM, in enough amounts to get a stock concentration of 10 mM. This stock solution was further diluted to a working solution (10 µM) in cell-specific medium and directly added to the 3D SISmuc models, in both compartments, for 24 h. For transforming growth factor beta 1 (TGFB1) stimulation, recombinant human TGFB1 (Peprotech, 100–21–1006, Germany) was dissolved in PBS- containing 1 % BSA (Carl Roth, 01633, Germany) at a concentration of 10 µg/mL. This stock concentration was diluted to 10 ng/mL in cell-specific medium prior to use and directly added to the 3D SISmuc models after 3 days of culture for the remaining 11 days.

#### 2.1.4 HE staining

Paraformaldehyde (4 % solution, Carl Roth GmbH, Karlsruhe, Germany) fixated and paraffin-embedded samples were cut into 3 µm sections with a microtome (SM 2010R, Leica, Germany). After deparaffinization and rehydration, tissue models were stained in hematoxylin and eosin (Morphisto, Offenbach am Main, Germany), each for 6 min according to the manufacturer’s protocol.

#### 2.1.5 Viability assay

The CellTiter-Glo®Luminescent Viability assay (Promega, USA) was used to determine viability after treatment with different GEM concentrations according to the manufacturer’s protocol. In short, cells were seeded into a 96-well plate with white borders and a transparent bottom and incubated overnight. On the following day, treatments at specified concentrations were administered concurrently with a medium change. On the fifth day, the medium was removed, and the wells were washed with PBS. Subsequently, 100 μL of medium was added, followed by the application of the CellTiter-Glo® reagent diluted 1:2 in cell culture medium. This was mixed for 1 min and incubated for 10 more min inside the microplate reader (TECAN, Männedorf, Switzerland) without shaking before luminescence was measured with an integration time of 1 s.

#### 2.1.6 Immunofluorescence staining

After fixation in 4 % paraformaldehyde and embedding, SISmuc models were sectioned at a microtome (SM 2010R, Leica, Germany), deparaffinized and rehydrated as previously described [29], followed by 20 min of heating at 100°C in a working solution of citrate buffer pH 6 (42 g/L citric acid monohydrate (Carl Roth, 1002441000, Germany) + 17.6 g/L NaOH in deionized water) inside a steam cooker (Braun, FS20, Germany). Subsequently, the samples were placed in deionized water, and sections were bordered with ImmEdge® Hydrophobic Pen (Vector Laboratories, H-4000, distributed by Biozol, Germany) before being transferred to PBS-T (PBS+ with 0.5 % tween®-20 (Sigma-Aldrich, P9416, Germany)). Afterwards, slides were placed in a moisture chamber and blocked for 20 min with 100 μL of 5 % donkey serum (Sigma-Aldrich, D9663, Germany) in antibody diluent solution (DCS Innovative Diagnostic Systems, AL120R500, Germany). The sections were then covered with the primary antibodies targeting COLIV (Abcam, ab6586, Germany), vimentin (Abcam, ab92547, Germany), cytokeratins (Sigma-Aldrich, C2562–.2 ML, Germany), diluted 1:100 in antibody diluent solution, and incubated overnight at 4°C. For each replicate, a negative control was included using only the antibody diluent solution without the primary antibody. After overnight incubation, samples were washed three times with PBS-T for 5 min each before adding the secondary antibodies anti-mouse conjugated with Alexa Fluor™ 657 (Life Technologies, A-31571, Germany), anti-rabbit conjugated with Alexa Fluor™ 555 (Life Technologies, A-31572, Germany), diluted 1:400 in antibody diluent solution, followed by 1 h incubation at RT, protected from light. After three washes with PBS-T in the dark, the samples were mounted with Fluoromount-G™ with DAPI and allowed to dry overnight before imaging. Images were taken with a fluorescence microscope (BZ-9000, Keyence, Osaka, Japan).

#### 2.1.7 Sample preparation for scRNAseq

Cell culture medium of 3D models was removed from both sides of the cell crown and carefully washed three times with room temperature PBS. Then, 1 mL of prewarmed Accutase® (Sigma-Aldrich, A6964, Germany) was pipetted into the luminal side and incubated for 30 min, mixing every 10 min using a micropipette. The Accutase® was then collected and transferred to a 15 mL centrifuge tube. Subsequently, the models were incubated for an additional 10 min with 10 mg/mL Protease (Bacillus licheniformis, subtilisin A, P5380, Sigma-Aldrich, Germany) at room temperature, mixed twice, each time after 5-minute intervals. The resulting suspension was collected into a 15 mL tube, and the cell crown was then washed twice with 1 mL PBS. This washing volume was also collected.

The total volume was adjusted to 5 mL and centrifuged at 300 g for 5 min. The pellet was resuspended in the desired volume for cell counting or other subsequent analyses, subjected to sorting to eliminate dead cells using a dead cell removal kit (Miltenyi Biotec, 130–090–101, Germany) as the manufacturer indicates. Following this, the cell concentration was adjusted to the desired level for optimal sequencing performance. The cells were then processed according to the tagging and preparation protocols outlined by 10X Genomics [30]. This involved labeling individual cells with specific barcodes to track their RNA expression profiles.

Once the cells were tagged, they were pooled together in quantities indicated in Table M 1. The prepared cell mixture was then carefully aliquoted and sent to the sequencing facility for single-cell RNA sequencing.

**Table M 1:** Information on the cells of the respective samples^1^.

Sample ID	Cells	Median reads per cell	Median genes per cell	Total genes detected	Median UMI counts per cell
CTRL_1	2973	52,670	3720	25,624	16,486
CTRL_2	450	53,272	3791	20,587	16,768
CTRL_2D	4819	76,947	5594	28,105	24,178
GEM_2	422	65,830	4349	20,817	20,999
TGFB1_1	2025	44,351	3824	25,511	13,892
TGFB1_2	359	45,931	3983	20,729	14,178
TGFB1_GEM_1	386	46,087	4135	20,921	14,577
TGFB_GEM_2	485	48,493	4150	21,673	15,154

1 CTRL = untreated, GEM = gemcitabine treated cells, TGFB = TGFB1 treated cells, and TGFB_GEM = TGFB1 and gemcitabine treated cells

#### 2.1.8 Single-cell RNA sequencing

Single-cell RNA sequencing was conducted by HIRI (Helmholtz Institute for RNA-based Infection Research; Single Cell Center) utilizing the Cell Ranger pipeline (version 7.0.1) up to the Cell Ranger multi step. Single Cell 3′ v3 chemistry was used, and introns were included in the analysis. Cell Ranger Multi, in particular 3’ GEX with Cell Multiplexing, multiple CMOs/samples was performed (see Supplementary Material for details). Eight different tags were used for the analysis (Table M 2) [30].

**Table M 2:** Tag information^1^.

Samples	CTRL_1	CTRL_2	GEM_2	TGFb1_1	TGFb1_2	TGFb1_GEM	TGFb1_GEM2	CTRL_2D
CMOs	301	302	304	305	306	307	308	309

1 CTRL = untreated, GEM = gemcitabine treated cells, TGFb = TGFb treated cells, and TGFb_GEM = TGFB1 and gemcitabine treated cells; CMO = Cell Multiplexing Oligo

### 2.2 Computational analysis

#### 2.2.1 Data preparation

For each sample, the single-cell data was read into R and transformed into a Seurat object using Seurat [31], [32], [33], [34], [35] (version 4.3.0). Only single cells with nCount_RNA > 800, nFeature_RNA > 500, and less than 10 % of mitochondrial genes were kept for the subsequent analyses. After following the Seurat preprocessing workflow, doublets were removed with DoubletFinder [36] (version 2.0.4). The respective samples were merged into single Seurat object for downstream analysis, containing all experimental groups (control (CTRL), gemcitabine treated cells (GEM), TGFB1-treated cells (TGFB1), and cells treated with a combination of gemcitabine and TGFB1 (TGFB1 +GEM)), and the standard workflow to generate UMAP visualizations was performed after removing mitochondrial and ribosomal genes and cell markers (CMOs).

This Seurat object was subsequently analyzed using Seurat (FindMarkers()) as well as pseudobulk analysis, and using mutual information-based gene selection with functional and machine learning-driven evaluation.

Details on data preparation are provided in the Supplementary Material.

#### 2.2.2 Data analysis and visualization in Seurat

The Seurat object was analyzed with Seurat’s FindMarkers function [31], [32], [33], [34], [35], performing pairwise comparisons across all four groups (untreated control cells (CTRL), cells treated with gemcitabine (GEM), cells treated with transforming growth factor beta 1 (TGFB1), and cells treated with both (TGFB1 +GEM)). Genes of interest were visualized as DimPlots and Violin Plots.

#### 2.2.3 Pseudobulk analysis

After preprocessing the merged Seurat object according to the standard workflow, the expression values for the respective identity classes (e.g., CTRL and TGFB1) were aggregated using Seurat’s AggregateExpression() function [31], [32], [33], [34], [35] to perform pseudobulk analysis, and genes with less than ten reads were removed before performing DESeq2 [37] (version 1.44.0) analysis using ‘apeglm’ for LFC shrinkage [38].

Differentially expressed genes (DEGs) with an adjusted p-value < 0.05, and an absolute log2 fold change greater than 1 (<−1 for downregulated genes and > 1 for upregulated genes) were selected for subsequent visualization and downstream enrichment analyses.

The DEGs obtained via DESeq2 analysis of the pseudobulk data were visualized as heatmaps and volcano plots using pheatmap [39] (version 1.0.12) and EnhancedVolcano [40] (version 1.22.0), respectively.

#### 2.2.4 Enrichment analysis

Enrichment analysis was performed using clusterProfiler (version 4.12.6) [41], [42] combined with the R-packages org.Hs.eg.db [43] (version 3.19.1) for Gene Ontology (GO) biological processes (BPs) and msigdbr [44] (version 10.0.1) for Hallmarks (Homo sapiens, category = H).

### 2.3 Mutual information-based gene selection with functional and machine learning-driven evaluation

Single-cell gene expression matrices were prepared following the workflow described in Caliskan et al. (2023) [24] and Caliskan et al. (2025) [26], using the Seurat-based pipeline provided at https://github.com/AC-PHD/Seurat_PFA_pipeline. Cell-level counts were aggregated by condition, and gene expression values were written into CSV files. Each file included gene identifiers (rows) and sample columns, with group labels encoded in the first row (e.g., 0 = control, 1 = treatment).

#### 2.3.1 Dimensionality reduction and condition-level embedding

To explore global transcriptional structure, we applied UMAP and t-SNE on normalized gene expression data using Scanpy [45], [46] (version 1.9.3). Preprocessing steps included total count normalization, log1p transformation, and PCA. Condition labels were overlaid for visualization. Embeddings were generated using all genes.

#### 2.3.2 Mutual information-based feature ranking

To identify genes with high discriminatory power between experimental groups, we computed mutual information (MI) between each gene’s expression profile and the binary condition labels. MI was calculated using gSELECT [26], which is based on the implementation from https://github.com/LauritzR/Principal-Feature-Analysis, as described in Rasbach et al. (2024) [25]. We set min_n_datapoints_a_bin = 50. The output was a ranked list of genes based on their MI values. Complete results are provided in the Supplementary Material.

#### 2.3.3 Supervised learning and model evaluation

Multilayer perceptron (MLP) classifiers were trained on three different gene selection strategies to assess the predictive power of MI-ranked genes:

(1) top-ranked genes by MI,

(2) randomly selected genes of equal number, and

(3) all non-constant genes, meaning only genes are considered where not each expression value over all cells is the same.

Each model was evaluated over 20 random train/test splits (80 %/20 %). Balanced accuracy, standard deviations, and mean misclassification rates were computed for each run.

Results were calculated and visualized using gSELECT [26], which employs plotting routines in matplotlib and seaborn. Line plots depicted balanced accuracy trajectories across sweeps (train vs. test), while bar plots summarized misclassification rates with standard deviation.

#### 2.3.4 Violin plot visualization of selected genes

Expression distributions of selected MI-ranked genes and known pathway-associated markers were visualized using violin plots grouped by the experimental condition. Log-normalized values were plotted using Seaborn-based panels with consistent axis scaling and labeling.

#### 2.3.5 Enrichment analysis

Functional enrichment analysis was performed on the top 10 MI-ranked genes using gseapy (v1.0.5) [47], querying MSigDB Hallmark [48], GO Biological Process (2025) [49], and KEGG [50], [51] 2021 gene sets. Significance was defined as FDR-adjusted p ≤ 0.05. Bar and dot plots were generated for each category and exported together with Excel result tables.

#### 2.3.6 STRING analysis

To create the respective STRING analysis visualizations, we used the STRING database [52], [53], [54], version 12.0, which visualizes protein associations, such as associations in curated databases, co-expression, or experimental and biochemical data.

#### 2.3.7 Heatmap generation

Expression values for top-ranked genes were visualized in z-scaled heatmaps. Cell-level clustering was performed per group using Ward’s method on Euclidean distances. Columns were annotated by group, and cluster order was preserved. Separate heatmaps were generated for (a) the top 10 MI genes and (b) a manually curated gene panel.

#### 2.3.8 Leiden clustering and transcriptomic subgroup annotation

To identify transcriptionally coherent subpopulations across treatment conditions, we performed unsupervised clustering using the Leiden algorithm (tl.leiden of Scanpy [45]). Raw expression counts (CSV format) were first loaded into an AnnData object, excluding features with technical identifiers (e.g., genes starting with “CMO”). The CSV files used in this analysis were generated as described in Caliskan et al. (2023) [24] and Caliskan et al. (2025) [26], using the Seurat-based pipeline available at https://github.com/AC-PHD/Seurat_PFA_pipeline. Cell-level gene expression matrices were aggregated by condition and exported into CSV format, with genes in rows and cells in columns. The first row of each file encoded the group label, using 0 for control cells and 1 for GEM-treated cells. For the present analysis, we specifically used the CSV file in which GEM-treated cells were labeled as 1 and control cells as 0. Data were normalized to 10,000 counts per cell, log-transformed, and scaled to unit variance (Scanpy pp.normalize_total, pp.log1p, and pp.scale, respectively). Principal component analysis (PCA) was performed using 30 components, which were used for kNN graph construction and subsequent UMAP embedding for 2D visualization. Leiden clustering was performed at a resolution of 0.095 to delineate transcriptionally distinct subgroups (tl.leiden in Scanpy). Cells were annotated by combining cluster assignment and treatment status, resulting in labels such as “GEM_1” or “CTRL_0”. These composite labels (“Group_Label”) enabled stratified downstream comparisons between matching and mismatching cluster-treatment combinations.

#### 2.3.9 Data preparation and distance-based definition of predicted-sensitive cancer cells for transcriptome-based classification

To explore whether transcriptional heterogeneity among untreated cells reflects latent treatment susceptibility, we developed a retrospective stratification strategy based on transcriptional similarity to GEM-treated cells. We assume that the untreated control group consists of both sensitive and non-sensitive cells with regard to GEM, and that the non-sensitiveness is not acquired during therapy but rather already exists prior to it. The assumption is supported by the finding that the GEM-treated cells and the control group cells are projected into a shared area, and a ML model cannot separate them well based on the gene expression. In case of a bigger change of the expression profile of the resistant-susceptible cells by GEM-treatment, they should be projected separated from the GEM-treated cells (resistant) or classification with high accuracy should be possible. Therefore, if we further assume that the GEM treatment does not significantly affect the gene expression of non-sensitive cells, then cells in the untreated control group whose expression profiles are close to those of GEM cells might be labeled as non-sensitive, while those far away might be considered sensitive. Raw expression counts (CSV input) were preprocessed by excluding technical artifacts (e.g., “CMO” genes), followed by library size normalization (10,000 counts per cell), log-transformation, and z-score scaling. Highly variable genes (n = 2000) were selected using the Seurat v3 flavor, and PCA was performed on the normalized data (30 components). Several strategies can be used to assess transcriptional similarity between untreated control and GEM-treated cells, including different dimensionality reduction methods and distance metrics. We compared combinations of PCA- and t-SNE–based projections with multiple distance metrics (e.g., minimal and mean cosine distance, Euclidean distance), see the Supplementary Material for details “Transcriptome-Based Retrospective Stratification of Untreated Cells”. Stratification based on minimal cosine distance in PCA space yielded the most biologically interpretable results. We remark that causally related genes (i.e., have a biological meaning) should ensure that a model can classify with high accuracy but not necessarily with the highest accuracy possible. There are genes whose expression correlates well with a phenotype and allows a high prediction accuracy but are not causally related, i.e. changing the corresponding gene expression does not change the phenotypic observations. Consequently, if it turns out in a subsequent analysis/experiment that identified genes, which contain information to separate the phenotypes, are only correlating, one can vary a part in the pipeline, e.g., change the distance measure or delete the corresponding correlating genes from the data set. Then one can repeat the analysis to find other genes that separate the phenotypes with high accuracy but might be causally related to the phenotypes, i.e. changing the expression of the genes impacts the phenotypic observations. Since genes that are causally related with the phenotypes to be separated should provide high accuracy for predicting them, causally related genes should be among the candidates that provide a model classifying with high prediction accuracy. We remark that UMAP can also be used instead of t-SNE and since UMAP can project into spaces with more than two dimensions, UMAP can also be an alternative to PCA.

**Fig. 1:**
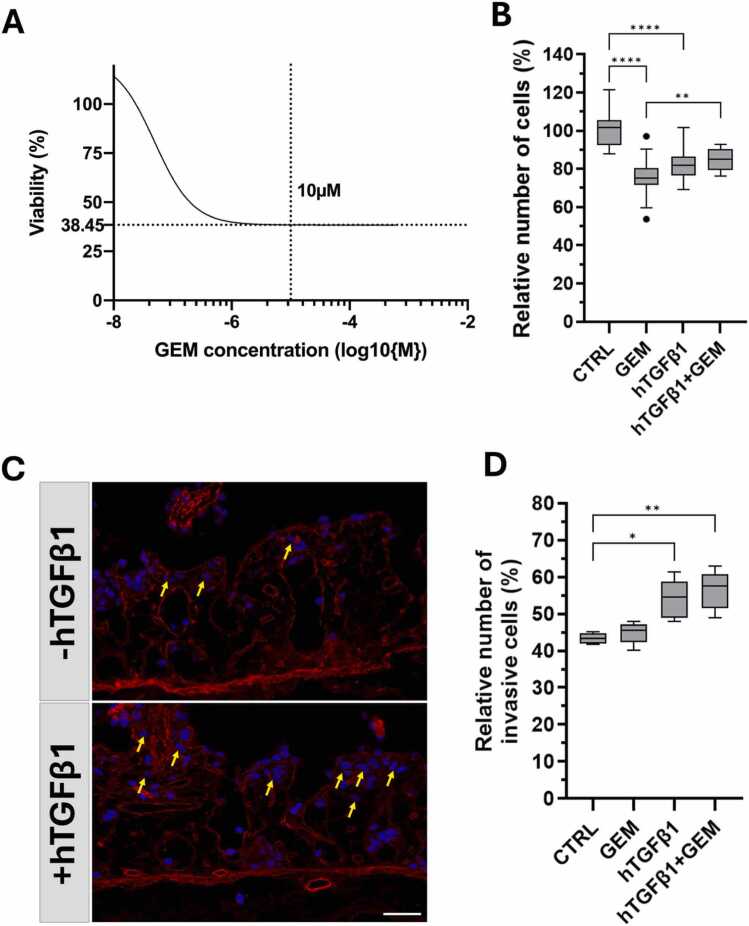
PANC-1 SISmuc 3D models show increased GEM resistance compared to 2D cell culture. (A) Viability of 2D PANC-1 cells treated with varying concentrations of gemcitabine (GEM) for 24 h, expressed as relative luminescence units (RLU) determined with CellTiterGlo® and normalized to untreated control. Representative graph of n = 3. Intersect shows remaining viability of PANC-1 cells after treatment with 10 µM GEM. **(B)** Tukey’s boxplot of cell numbers in PANC-1 SISmuc 3D models after 14 days of culture, percentages were calculated against the median value of the untreated control, GEM treatments were applied for 24 h, n = 3. Significance was determined with *t*-test, **** = p < 0.001. **(C)** Representative Immunofluorescence staining of collagen IV (COL IV, red in C) and PANC-1 cells nuclei (DAPI, blue in C) showing invasive cells (arrows). **(D)** Quantification of invasive cells calculated as the percentage of cells within matrix structures divided by the total count of cells in each condition, n = 3. Significance was determined with *t*-test, * = p < 0.1; ** = p < 0.001; **** = p < 0.001. Scale bar = 100 µm.

Cosine distances between each untreated control cell and each GEM-treated cell were calculated in PCA space. To assess the GEM-similarity of each control cell, the minimal cosine distance among all GEM-treated cells is taken as a measure of GEM-similarity (alternatively, the mean for the “mean” option instead of the before explained “min” option). Based on this definition, control cells are identified as similar to GEM-treated cells and labeled as “predicted-resistant”, while the control cells most dissimilar to GEM-treated cells were labeled as “predicted-sensitive”. From the experimental results, where about 25 % a cell group dies upon GEM treatment (see Fig. 1B, CTRL and GEM-treated cells), a rule of thumb is that the 75 % of cells with the highest GEM-similarity in the untreated control group could be considered as predicted-resistant and thus these cells should not die upon GEM treatment. All GEM-treated cells were labeled as “GEM”. These three groups were combined into a subset AnnData object for downstream analyses. A pseudo code of the method to identify the responders and the resistant cells within the control group is given in the Extended Methods of the Supplementary Material.

For our approach, we assume that GEM-treated cells represent transcriptionally resistant survivors, as dying or dead cells are typically lost during the single-cell capture process. Consequently, we assume that control cells that are highly dissimilar to GEM survivors might not survive during GEM-treatment, which might be due to differences in gene expression. Analogously, we assume that the similar control (Ctrl) cells would survive if we treated this group. Therefore, control cells showing transcriptional differences to GEM-treated cells were labeled as “predicted-sensitive”. Conversely, control cells that closely resemble GEM-treated cells are labeled as “predicted-resistant” (here sometimes referred to as resistant), as the similarities between untreated cells and GEM-treated cells might indicate that these cells would survive GEM-treatment.

To analyze the threshold for the cosine distance to define what is close enough to GEM cells to be classified as non-sensitive, and extend the insight of the experimental study shown in Fig. 1 regarding cell survival, we trained a ML model on the z-score normalized expression profile of all genes to classify the cells into predicted-sensitive and predicted-resistant and monitored the accuracy depending on the percentage of sensitive cells with regard to the whole Ctrl group. High accuracy might indicate that the expression profiles can be uniquely associated with phenotypes and the corresponding characteristics that allow the separation with high accuracy might be promising candidates to understand the resistant behavior of the corresponding cells. The following procedure was used to label untreated control cells as predicted-resistant. Starting with the Ctrl cells with the smallest distance to one GEM cell (or alternatively within any other distance measure, see the Supplementary Material), going through the control cells in ascending order with respect to the distance to one GEM cell until a predefined percentage is reached. Under the assumption that a sharp expression change is associated with the clear phenotypic difference between GEM-sensitivity and non-sensitivity, we hypothesize that the highest accuracy within classifying the groups coincides with the best distance between GEM and untreated control cells, thus the best purity of the corresponding groups, enabling the separation of corresponding phenotypes based on their expression profiles. Thus, the distance ranking with the corresponding percentage cutoff should capture a sharp change in the expression change. For a predicted-sensitive percentage of 5 %, only 5 % of the Ctrl cells, the most distant to GEM cells, were labeled as predicted-sensitive, while the rest of the cells were labeled as predicted-resistant. Similarly, a predicted-sensitive percentage of 15 % can be understood as two groups of control cells consisting of the most distant 15 % of control cells with regard to GEM cells, which appear to be most likely to respond to GEM-treatment, and the remaining 85 % of control cells, which show an increasing similarity to GEM-treated cells.

With an increasing predicted-sensitive percentage, a greater number of cells are classified as predicted-sensitive, and the criteria for classification become less stringent, which can decrease the precision of the classification, since true resistant cells might be labeled as sensitive cells when the number of cells in the predicted-sensitive group increases if the assumption is correct that the resistant cells can be separated from the predicted-sensitive cells by a characteristic expression pattern. In this case of wrongly labeled cells, the expression profile does not allow a highly accurate classification between our distance-defined predicted-sensitive and predicted resistant cells, as cells with the corresponding expression profile appear in a significant number in both classes. The script used for this evaluation is part of the python library gSELECT [79], and is also available via GitHub at https://github.com/CaliskanDeniz/gSELECT.

The scripts for calculating the distance ratings are available at https://github.com/AC-PHD/calculating-multiple-distance-metrics.

#### 2.3.10 Evaluation of class separation via supervised machine learning

To assess whether the stratified predicted-sensitive groups might be biologically meaningful, we applied a supervised machine learning classification using gSELECT [26]. Similar to the ML algorithm previously described in Rasbach et al., which is able to distinguish between two different groups (e.g., different cell types or different conditions), the resulting MI genes are based on ranked mutual information [25].

For our analyses, we defined three groups of cells: predicted-sensitive, predicted-resistant, and GEM-treated cells. While the group of GEM-treated cells always contained all GEM-treated cells, the number of control cells labeled as predicted-sensitive or predicted-resistant varied according to their respective predicted-sensitive percentages, caused variations in the threshold for the cosine distance, as described above. Supervised machine learning classification was performed for the binary classification of predicted-sensitive vs. GEM against predicted-sensitive-percentage thresholds across 23 data points (e.g., top 5 %, 10 %, …, 100 % of control cells labeled as predicted-sensitive, ranked according to their minimal cosine distance from GEM-treated cells).

#### 2.3.11 Survival analysis

To further assess the biological relevance of the MI genes, survival analyses were performed using the online tool Kaplan-Meier Plotter for pancreatic cancer [55], available at https://server2.kmplot.com/pancreas, accessed 29th September 2025. The tool uses data from several databases, including the GEO database [56] and the International Cancer Genome Consortium Data Portal on pancreatic carcinomas [55]. The tool is constantly being improved and expanded to include more data on the different cancer types that can be analyzed, including colon cancer [57] and non-small-cell lung cancer [58].

## 3 Results

We aimed to generate more relevant preclinical models by seeding PANC-1 tumor cells on a biological matrix (SISmuc) to create a 3D model better mimicking PDAC tumors. These cells were either treated with GEM, a common chemotherapeutic drug for PDAC [8], stimulated with TGFB1, a factor which is secreted from the tumor stroma to drive the desmoplasmic reaction, which is predominant in PDAC [59], or both. Afterwards, we analyzed changes in gene expression comparing control cells (CTRL, untreated and unstimulated) with PANC-1 cells after treatment with either GEM, stimulation with TGFB1 (referred to as hTGFβ1 or TGFB1), or both (TGFB1 +GEM), aiming to find changes in gene expression related to GEM-treatment and possibly drug resistance.

### 3.1 PANC-1 cell heterogeneity affects drug response

The experimental results indicate that, under conventional 2D culture conditions, only approximately 38 % of PANC-1 cells survived 10 µM GEM treatment (Fig. 1A). However, in tissue-like 3D conditions, approximately 75 % of PANC-1 cells survived GEM treatment (Fig. 1A and Fig. 1B). Under stimulation with TGFB1 in 3D culture, the cell number decreased to approximately 80 % compared to the control, but the cell number was not further reduced by GEM-treatment (Fig. 1 B). This indicates the induction of complete resistance to GEM by TGFB1, accompanied by the further induction of invasion across the preserved basement membrane structure. Collagen IV (COL IV) staining (Fig. 1C) highlights crypt and villi architecture of the SISmuc-matrix and stains the basement membrane. Cells that crossed this structure to enter deeper layers of the intestinal tissue matrix (arrows, Fig. 1C) are quantified by counting (Fig. 1D).

**Fig. 2:**
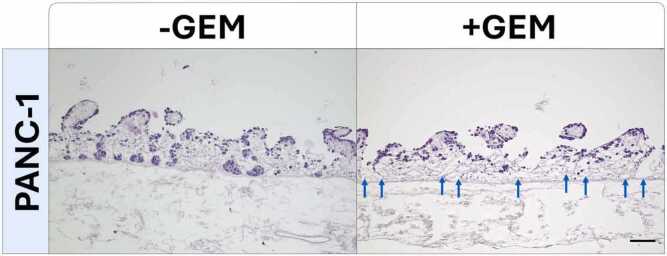
Niche-specific effect of gemcitabine treatment in PANC-1 3D SISmuc models. Representative Hematoxylin-Eosin (HE) stainings of formalin-fixed paraffin embedded samples of untreated (-GEM) and treated (+GEM) PANC-1 3D SISmuc models. Scale bar: 100 µm.

The *in vitro* results show that PANC-1 tumor cells cultured in 3D exhibit a higher basic chemoresistance to GEM compared to 2D conditions but still respond to GEM. This suggests the possible existence of PANC-1 subpopulations that are GEM-resistant in a non-concentration-dependent manner. To investigate this initial observation morphologically, the effect of GEM treatment was assessed using Hematoxylin-Eosin (HE)-stainings in the 3D SISmuc models. As this tissue matrix provides different niches, we investigated whether there are locations where the cells die first. Interestingly, we observed that cells that migrated to deeper crypt regions are first eradicated by GEM, indicating a high impact of this specific microenvironment on tumor cell survival (Fig. 2, blue arrows).

After generating models with high chemoresistance, as expected in PDAC patients, we analyzed differences in gene expression between the respective treatment groups (untreated control cells, GEM-treated cells, TGFB1-stimulated cells, or cells treated with TGFB1 +GEM).

**Fig. 3:**
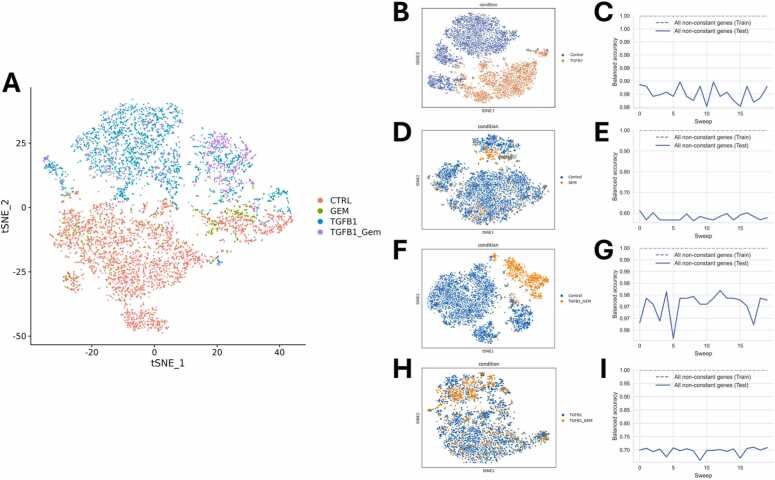
t-SNE visualization and balanced accuracy of pairwise group comparisons. **(A)** t-SNE visualization of all single cells of all samples, sorted by condition. **(B)** t-SNE visualization of the comparison between control (two biological replicates, resulting in 3152 single cells after quality control and filtering) and TGFB1-simulated cells (two biological replicates, resulting in 2194 single cells after quality control and filtering). **(C)** Visualization of the balanced accuracy from the machine learning classification, demonstrating a high prediction accuracy for the comparison between control and TGFB1-treated cells. **(D)** t-SNE visualization of the comparison between control (3152 single cells) and GEM-treated cells (one sample containing 392 single cells after quality control and filtering), illustrating the similarities between both conditions. **(E)** Balanced accuracy of the machine learning classification for control and GEM-treatment, indicating a limited ability to distinguish between the two conditions. **(F)** t-SNE plot illustrating the separation between control (n = 3152) and TGFB1 +GEM-treated cells (TGFB1_GEM, two biological replicates, 815 single cells after quality control and filtering). **(G)** Graphical representation of the balanced accuracy from machine learning, demonstrating robust discrimination between control and combination-treated cells. **(H)** t-SNE visualization of the comparison between TGFB1-treated cells (n = 2194) and cells treated with the combination treatment (TGFB1_GEM, n = 815). **(I)** Similar to the t-SNE plot, the low balanced accuracy of the machine learning results for TGFB1 versus combination treatment indicates rather high similarities between TGFB1-stimulation and TGFB1 +GEM-treatment.

Visualizing the different groups using t-distributed Stochastic Neighbor Embedding (t-SNE) indicates similarities between untreated and GEM-treated samples, as well as similarities between cells stimulated with TGFB1 and the combination of TGFB1 and GEM (Fig. 3A). Additionally, we visualized pairwise comparisons of the respective conditions using t-SNE plots. While the t-SNE plots of unstimulated and TGFB1-stimulated cells show a clear separation (Fig. 3B), untreated and GEM-treated cells are not clearly separated (Fig. 3D). Similarly, the clusters for control and TGFB1 +GEM-treated cells are clearly separated (Fig. 3F). In contrast, comparing TGFB1 stimulation and the combination of TGFB1 with GEM treatment does not result in clearly separated clusters (Fig. 3H).

Using all cells that remained after quality control and filtering using Seurat [31], [32], [33], [34], [35] to select cells with nCount_RNA > 800, nFeature_RNA > 500, and less than 10 % of mitochondrial genes, and removing doublets using DoubletFinder [36], as described in Data Preparation, we employed a machine learning algorithm to identify the most promising pairwise comparisons for in-depth analysis. Out of the two control samples (biological replicates), 3152 single cells remained. The two TGFB1 samples (biological replicates) yielded 2194 single cells, and the two biological replicates for PANC-1 cells treated with the combination TGFB1 +GEM contained 815 single cells after quality control and filtering. For GEM-treated cells, only one sample, containing 392 single cells after quality control and filtering, was available. For the machine learning (ML) analysis of the respective comparisons, 80 % of the cells of two conditions (e.g., control and TGFB1) were used to train the algorithm, while the remaining 20 % of the cells were used to test the algorithm. As features, we took all genes with their corresponding z-score normalized expression values. The resulting balanced accuracy indicates how well the algorithm was able to discern between the respective conditions after training.

For the clearly separated conditions control and TGFB1 (Fig. 3B), the accuracy of the prediction in training was 100 % (dotted line), and the test mean accuracy for 20 repetitions (solid line) was 98.51 % (Fig. 3C), indicating that the ML algorithm was able to clearly distinguish between the two conditions. For the comparison between control and GEM treatment (Fig. 3D), the balanced accuracy ranged from 50 % to 60 %, with a test mean accuracy of 58.01 % (Fig. 3E). Since the comparison involves only two conditions, the probability of assigning a sample to the correct group by random chance is 50 %. Therefore, a balanced accuracy of approximately 60 % indicates that the model’s performance is only marginally better than random guessing, suggesting that the machine learning algorithm has limited ability to differentiate between the conditions, indicating the similarity of untreated and GEM-treated cells. In other words, the surviving cells after GEM treatment are not distinguishable from untreated control cells by standard analysis. For the remaining two comparisons (control compared with combination (TGFB1+GEM) treatment (Fig. 3F and Fig. 3G) and TGFB1-stimulation compared with combination treatment (Fig. 3H and Fig. 3I)), the results were comparable. While the test mean accuracy for the comparison between control and TGFB1 +GEM was 97.59 % (Fig. 3F and Fig. 3G), the prediction accuracy for the comparison between TGFB1 and the combination was between 60 % and 70 %, with a test mean accuracy of 69.66 % (Fig. 3H and Fig. 3I). This relatively low prediction accuracy suggests that the model was unable to clearly distinguish between TGFB1-stimulated cells and those treated with the combination. The t-SNE plot (Fig. 3H) and the ML algorithm (Fig. 3I) coincide in their results, indicating that a low accuracy is not an artifact. This might indicate that GEM-treatment has only small effects on the gene expression of TFGB1-stimulated cells, which cannot be detected by standard methods.

**Fig. 4:**
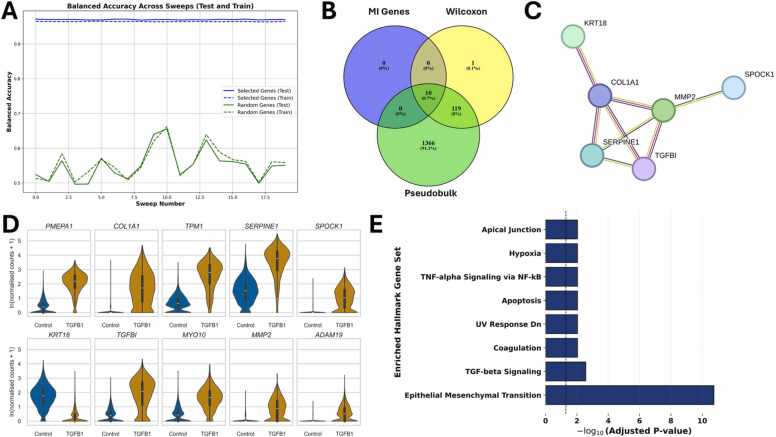
Analysis of the ten top-ranked mutual information (MI) genes for the comparison between control and TGFB1-stimulated cells. **(A)** The balanced accuracy of the ten top-ranked MI genes (blue lines, test mean accuracy of 97.01 %) is significantly higher than the balanced accuracy achieved by using the same number of randomly selected genes (green lines, test mean accuracy of 55.10 %). **(B)** Venn diagram demonstrating that all ten top-ranked MI genes (blue ellipse) are also among the differentially expressed genes (DEGs) identified using pseudobulk analysis and DESeq2 (green ellipse) and among the genes identified by using Seurat’s FindMarkers function with Wilcoxon Rank Sum test (yellow ellipse). **(C)** Visualization of the STRING analysis of the ten top-ranked MI genes (standard medium confidence of 0.4, with disconnected nodes hidden, using STRING version 12.0). **(D)** Violin plots visualizing the expression of the ten top-ranked MI genes in control (blue) and TGFB1-stimulated cells (amber). **(E)** Hallmark enrichment analysis based on the ten top-ranked MI genes (via gseapy (v1.0.5)).

Since the ML algorithm was able to distinguish between control and TGFB1 with very high prediction accuracy when using all available genes, and thus TGFB1 appears to induce significant changes in gene expression, we calculated the mutual information genes to obtain a small but meaningful set of genes that describe the differences. The algorithm ranks all available genes according to MI, with those most informative for distinguishing between the two conditions appearing at the top of the ranking. The balanced accuracy of the ten top-ranked MI genes (Fig. 4A, blue lines) was greater than 96 % in both training and testing, with a test mean accuracy of 97.01 %. When using ten randomly selected genes (Fig. 4, green lines) instead of the ten MI genes, the balanced accuracy for distinguishing between control and TGFB1-stimulated cells ranges from approximately 50–65 %, with a test mean accuracy of 55.10 %. Analyzing an equal number of random genes results in a much lower prediction accuracy than analyzing the ten top-ranked MI genes, which indicates the predictive power and the potential biological importance of the MI genes. To further validate the top-ranked MI genes, we performed pseudobulk analysis using DESeq2 [37] comparing control and TGFB1-stimulated cells and employed Seurat’s FindMarkers function using the Wilcoxon Rank Sum test. All ten top-ranked MI genes (Fig. 4B, green ellipse and Fig. 4D) are both among the identified marker genes (Fig. 4B, yellow ellipse), and among the differentially expressed genes (DEGs) according to DESeq2 analysis of the pseudobulk data of the two conditions (Fig. 4B, green ellipse).

Additionally, six of the MI genes (*KRT18*, *COL1A1*, *SERPINE1*, *TGFBI, MMP2*, and *SPOCK1*) have been associated with each other and are interconnected in a STRING visualization (Fig. 4C, using STRING [52], [53], [54] version 12.0, with a standard medium confidence of 0.4, with disconnected nodes hidden). While most of the MI genes show higher expression in the TGFB1-stimulated cells, KRT18 expression appears to be downregulated upon TGFB1 stimulation (Fig. 4D). Performing enrichment analysis of the ten top-ranked MI genes resulted in “Epithelial Mesenchymal Transition (EMT)”, “TGF-beta Signaling”, and “Apoptosis” as the most relevant Hallmarks associated with these MI genes (Fig. 4E).

To assess the biological relevance of the MI genes in pancreatic cancer, we performed survival analyses of the respective genes using the web-tool Kaplan-Meier Plotter [55], [57], [58] (access date 29th September 2025, available at https://server2.kmplot.com/pancreas). The respective plots are available in the Supplementary Material. According to survival analysis, high expression of the following key genes is associated with poor prognosis: *COL1A1*, *KRT18*, *MMP2*, *SERPINE1*, *ADAM19*, *PMEPA1*, *SPOCK1*, and *TGFBI*.

**Fig. 5:**
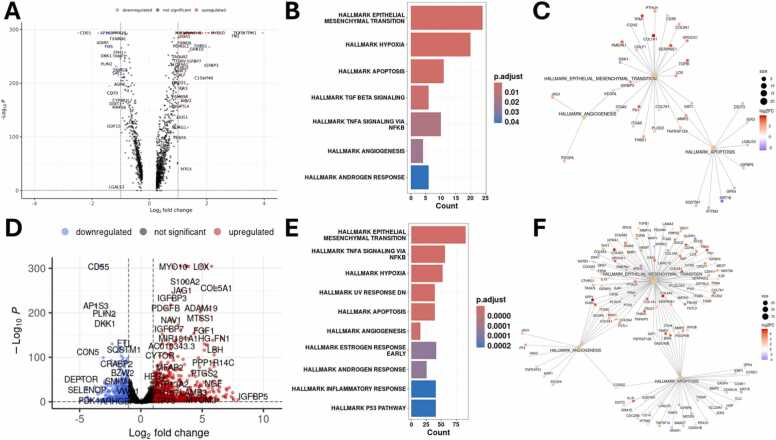
Visualizations of the differentially expressed genes and the respective enrichment analyses for the comparison between control and TGFB1-stimulated cells for Seurat and pseudobulk analysis. **(A)** Volcano plot visualizing the genes identified by Seurat’s FindMarker function when comparing control and TGFB1-stimulated cells using Wilcoxon Rank Sum test. **(B)** Enrichment analysis of the resulting genes from Wilcoxon Rank Sum test using msigdbr Hallmarks. **(C)** Cnet plot of the three Hallmarks “Epithelial Mesenchymal Transition”, “Angiogenesis”, and “Apoptosis” for the genes identified via FindMarkers with Wilcoxon Rank Sum test. **(D)** Volcano plot of the DEGs identified by pseudobulk analysis using DESeq2. **(E)** Enrichment analysis of these DEGs using msigdbr Hallmarks. **(F)** Cnet plot of the three Hallmarks “Epithelial Mesenchymal Transition”, “Angiogenesis”, and “Apoptosis” for the DEGs identified via pseudobulk analysis, visualizing the DEGs associated with the respective Hallmarks.

Subsequently, we analyzed the original Seurat object comparing control and TGFB1-treated cells via the FindMarkers function (Fig. 5A) and performed Hallmark enrichment analysis for the resulting genes using msigdbr (version 10.0.1), which provides ‘Molecular Signatures Database’ (MSigDB) gene sets [44], (Fig. 5B), before visualizing the genes associated with the three Hallmarks “Epithelial Mesenchymal Transition”, “Angiogenesis”, and “Apoptosis” (Fig. 5C). To further validate the results, we performed an additional pseudobulk analysis of the two conditions using DESeq2 after aggregating the respective identity classes with Seurat (Fig. 5D). For the resulting DEGs, enrichment analysis was performed using the *Homo sapiens* Hallmarks from msigdbr (version 10.0.1) (Fig. 5E), and the three Hallmarks “Epithelial Mesenchymal Transition”, “Angiogenesis”, and “Apoptosis” and their associated DEGs were visualized as a Cnet (gene concept network) plot (Fig. 5F). For both analyses, Wilcoxon and pseudobulk, the Hallmarks “Epithelial Mesenchymal Transition”, “Angiogenesis”, and “Apoptosis”, as well as “Hypoxia” and “TNFA Signaling via NFKB” were among the top ranked results. Additionally, some genes, such as KRT18, which is associated with apoptosis, were identified by both analysis methods, further validating their potential relevance.

**Fig. 6:**
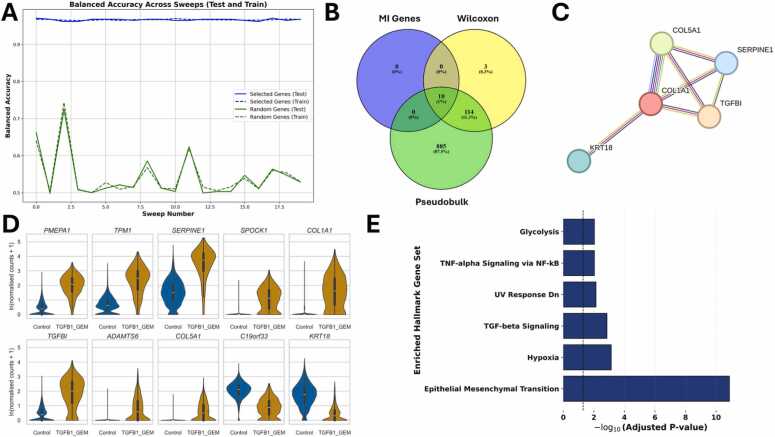
Analysis of the ten top-ranked mutual information (MI) genes for the comparison between control and TGFB1 +GEM-treated cells. **(A)** The balanced accuracy of the ten top-ranked MI genes (blue lines, test mean accuracy of 96.64 %) is significantly higher than the balanced accuracy achieved by using the same number of randomly selected genes (green lines, test mean accuracy of 54.34 %). **(B)** Venn diagram visualizing that all ten top-ranked genes identified by machine learning (blue ellipse) are also among the differentially expressed genes (DEGs) identified using pseudobulk analysis and DESeq2 (green ellipse), and among the genes identified by using Seurat’s FindMarkers function with Wilcoxon Rank Sum test (yellow ellipse). **(C)** Visualization of the STRING analysis of the ten top-ranked MI genes (standard medium confidence of 0.4, with disconnected nodes hidden, using STRING version 12.0). **(D)** Violin plots visualizing the expression of the ten top-ranked MI genes in control (blue) and TGFB1-stimulated cells (amber). **(E)** Hallmark enrichment analysis based on the ten top-ranked MI genes (via gseapy (v1.0.5)).

Our ML pre-analysis also resulted in a high prediction accuracy for the comparison between control and combination treatment (TGFB1 +GEM). Thus, we repeated the above-described analysis steps for the respective comparison to predict expression changes possibly related to resistance-induction by TGFB1 (Fig. 6). The ten top-ranked genes of this comparison resulted in a test mean accuracy of 96.64 % (blue lines in Fig. 6A), while ten randomly selected genes only yielded a test mean accuracy of 54.34 % (green lines in Fig. 6A), indicating the potential relevance of the genes identified using mutual information. Their relevance was further confirmed by the Wilcoxon Rank Sum test and pseudobulk analysis (Fig. 6B). Five of the ten MI genes are also associated with each other according to the STRING database (Fig. 6C), and most of the top-ranked genes identified via machine learning are upregulated after the combination treatment (Fig. 6D); only *KRT18* and *C19orf33* are more highly expressed in the control compared to the combination treatment. We could see the same upregulation for *PMEPA1*, *TPM1*, *SERPINE1*, *SPOCK1*, *COL1A1,* and *TGFBI* within the combination as in the comparison between the control and TGFB1. Only the upregulation of *ADAMTS6*, *COL5A*, and the downregulation of *C19orf33* are specific to TGFB1 stimulated cells treated with GEM, whereas *MYO10*, *MMP2,* and *ADAMT19* are only upregulated in TGFB1-stimulated cells compared to the control. Similar to TGFB1-stimulation (see Fig. 4E), the Hallmarks Epithelial Mesenchymal Transition and TGF-beta Signaling were among the top-ranked enriched Hallmarks (Fig. 6E). Interestingly, Glycolysis was also in the top-ranked enriched Hallmarks, indicating more metabolically active cells in TGFB1-stimulated conditions when treated with GEM.

Survival analyses of the MI genes were performed to compare the control and TGFB1 +GEM-treated cells, examining their biological relevance in pancreatic cancer. The respective plots, which were generated using the web-tool Kaplan-Meier Plotter [55], [57], [58] (access date 29th September 2025, available at https://server2.kmplot.com/pancreas), are available in the Supplementary Material. Key MI genes associated with a bad prognosis according to survival analysis include: *COL1A1*, *KRT18*, *SERPINE1*, *PMEPA1*, *TPM1*, *SPOCK1*, and *TGFBI*.

**Fig. 7:**
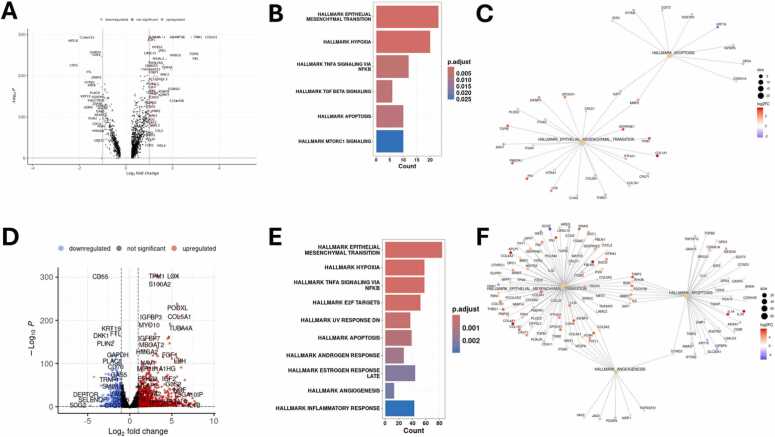
Visualizations of the differentially expressed genes and the respective enrichment analyses for the comparison between control and TGFB1 +GEM-treated cells for Seurat and pseudobulk analysis. **(A)** Volcano plot visualizing the genes identified by Seurat’s FindMarker function when comparing control and TGFB1 +GEM-treated cells using Wilcoxon Rank Sum test. **(B)** Enrichment analysis of the resulting genes from Wilcoxon Rank Sum test using msigdbr Hallmarks. **(C)** Cnet plot of the Hallmarks Epithelial Mesenchymal Transition and Apoptosis for the genes identified via FindMarkers with Wilcoxon Rank Sum test. **(D)** Volcano plot of the DEGs identified by pseudobulk analysis using DESeq2. **(E)** Enrichment analysis of these DEGs using msigdbr Hallmarks. **(F)** Cnet plot of the three Hallmarks “Epithelial Mesenchymal Transition”, “Angiogenesis”, and “Apoptosis” for the DEGs identified via pseudobulk analysis, visualizing the DEGs associated with the respective Hallmarks.

To further validate these results, we used the Seurat function FindMarkers with Wilcoxon Rank Sum test (Fig. 7A-C) and pseudobulk analysis (Fig. 7 D-F) to identify differences in gene expression between control and combination. The identified marker genes are visualized as a volcano plot in Fig. 7A; the results of the enrichment analysis using msigdbr and the *Homo sapiens* Hallmarks are shown in Fig. 7B. Since “Angiogenesis” was not among the identified Hallmarks, the Cnet plot in Fig. 7C only visualizes the genes associated with the two Hallmarks “Epithelial Mesenchymal Transition” and “Apoptosis”. The same analyses were repeated for the pseudobulk analysis of control and combination (TGFB1 +GEM). The identified DEGs are shown as a volcano plot (Fig. 7D) and were used for Hallmark enrichment analysis with the misigdbr Hallmarks (Fig. 7E). The DEGs associated with the Hallmarks “Epithelial Mesenchymal Transition”, “Angiogenesis”, and “Apoptosis” are visualized as a Cnet plot in Fig. 7F.

**Fig. 8:**
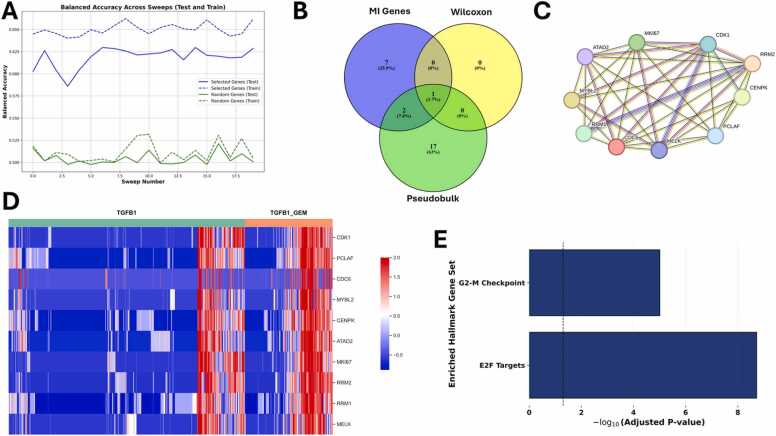
Analysis of the ten top-ranked mutual information (MI) genes for the comparison between TGFB1-stimulated cells and TGFB1 +GEM-treated cells. **(A)** The balanced accuracy of the ten top-ranked MI genes (blue lines, test mean accuracy of 61.85 %) is only slightly higher than the balanced accuracy achieved by using the same number of randomly selected genes (green lines, test mean accuracy of 50.40 %). **(B)** Only one gene (PCLAF) is among the top-ranked genes identified by machine learning (blue ellipse), among the differentially expressed genes (DEGs) identified using pseudobulk analysis and DESeq2 (green ellipse), and among the genes identified by using Seurat’s FindMarkers function with Wilcoxon Rank Sum test (yellow ellipse). **(C)** Visualization of the STRING analysis of the ten top-ranked MI genes (standard medium confidence of 0.4, with disconnected nodes hidden, using STRING version 12.0) shows that all of the genes are highly interconnected. **(D)** Heatmap visualizing the expression of the ten top-ranked MI genes for each cell (TGFB1-stimulated cells indicated by a green bar, cells treated with the combination treatment indicated by an orange bar, upregulation shown in red, downregulation in blue). **(E)** Hallmark enrichment analysis based on the ten top-ranked MI genes (via gseapy (v1.0.5)) results only in cell cycle-related Hallmarks.

Finally, we compared TGFB1-stimulated cells and TGFB1 +GEM-treated cells as these should give the best prediction about resistance-related DEGs. Since these cell clusters are not clearly separated (Fig. 3H), identifying differentially expressed genes to distinguish between the two groups is challenging (Fig. 3I and Fig. 8A). Although the balanced accuracy for the ten top-ranked genes (Fig. 8A, blue lines, with a test mean accuracy of 61.85 %) was only slightly higher than the balanced accuracy of ten randomly selected genes (Fig. 8A, green lines, with a test mean accuracy of 50.40 %), and only one gene (*PCLAF*) was identified by all methods (machine learning, FindMarkers with Wilcoxon Rank Sum test, and pseudobulk analysis, see Fig. 8B), the ten genes are highly interconnected (Fig. 8C). Visualizing the expression of the ten genes as a heatmap (Fig. 8D) indicates that, in most TGFB1-stimulated cells, their expression was downregulated. In contrast, a greater proportion of the TGFB1 +GEM-treated cells displayed upregulation of these genes. Since the Hallmarks associated with these DEGs (Fig. 8E), E2F Targets and G2-M Checkpoint, are associated with the cell cycle, TGFB1 +GEM treatment might affect the cell cycle more than TGFB1 stimulation alone.

For the MI genes resulting from the comparison between TGFB1-stimulated cells and cells treated with TGFB1 and GEM, survival analyses were performed with the Kaplan-Meier Plotter web-tool [55], [57], [58] (access date 29th September 2025, available at https://server2.kmplot.com/pancreas). The respective plots are available in the Supplementary Material. According to survival analysis, key genes include *MYBL2*, *MELK*, and *ATAD2*, whose higher expression correlates with worse survival.

**Fig. 9:**
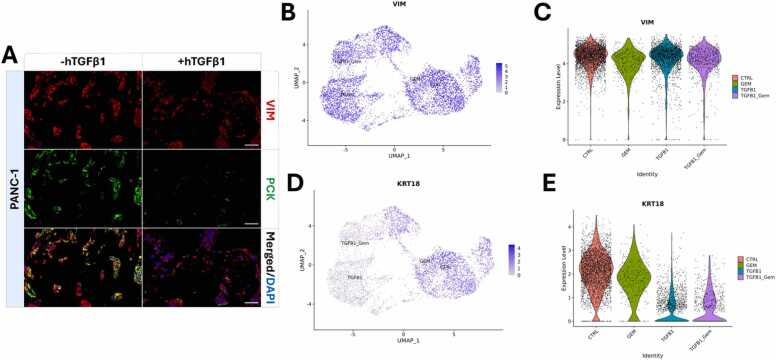
Expression of VIM and KRT18 in TGFB1-stimulated PANC-1 cells in 3D-culture. **(A)** Representative immunofluorescence stainings of vimentin (VIM, red in A), cytokeratins (PCK, green in A) and PANC-1 cells nuclei (DAPI, blue in A) without (-hTGFβ1) and with (+hTGFβ1) stimulation with human Transforming Growth Factor beta 1 (hTGFβ1), scale bar: 100 µm. **(B)** UMAP visualization of VIM expression across all samples. **(C)** Violin plot visualizing the expression of VIM in untreated control (CTRL) cells, GEM-treated cells, TGFB1-treated cells, and cells treated with the combination of both TGFB1 and GEM (TGFB1 +GEM). **(D)** UMAP visualization of KRT18 expression across all samples. **(E)** Violin plot visualizing the expression of KRT18 in untreated control (CTRL) cells, GEM-treated cells, TGFB1-stimulated cells, and cells treated with the combination of both TGFB1 and GEM (TGFB1 +GEM).

The 3D *in vitro* model (Fig. 9A) was used to assess two common EMT markers, vimentin (VIM) and pan-cytokeratin (PCK). PCK stains nine cytokeratins (cytokeratin 1, 4, 5, 6, 8, 10, 13, 18, and 19), with cytokeratin 18 (KRT18) as an example for the *in silico* analysis. After TGFB1 stimulation, the expression of VIM was not changed, while PCK expression decreased (Fig. 9A). This effect of TGFB1 stimulation on VIM and PCK from fluorescence stainings of PANC-1 models was further confirmed by the expression in scRNAseq data. The UMAP visualizations of the expressions of VIM (Fig. 9B) and KRT8 (Fig. 9D), as well as the violin plots showing the expression level of both genes (VIM, Fig. 9C, and KRT Fig. 9E), indicate that VIM is equally expressed in all samples, while KRT18 is downregulated in TGFB1 and TGFB1 +GEM-treated cells.

Both the Seurat analysis, using the FindMarkers function employing the Wilcoxon Rank Sum test, as well as the pseudobulk analysis employing DESeq2 as analysis method, identified KRT8, KRT18, and KRT19 as markers/differentially expressed genes when comparing untreated or GEM-treated cells with TGFB1-stimulated (TGFB1 or TGFB1 +GEM) cells (Table 1). In addition to KRT8, KRT18, and KRT19, the pseudobulk analysis also identified further keratins as differentially expressed (KRT16, KRT17, KRT19, KRT32, KRT80). Notably, KRT17 was found to be strongly upregulated in TGB1-stimulated conditions both with and without GEM treatment compared to control conditions.

**Table 1:** KRT-Expression levels in the different pairwise comparisons a.

		**KRT gene**	**avg_log2FC**	**p_val_adj**
**DEGs Wilcoxon**	CTRL vs. GEM	no KRTs		
	CTRL vs. TGFB1	**KRT8**	**−1.45621192378374**	**0**
		**KRT18**	**−2.47424079333701**	**0**
		**KRT19**	**−1.77664494747206**	**0**
	CTRL vs. TGFB1 +GEM			
		**KRT18**	**−2.26229346037776**	**8.49132827622506E−286**
		**KRT8**	**−1.47063609307494**	**2.06473381166114E−177**
		**KRT19**	**−1.72946679767881**	**4.49976770213092E−151**
	GEM vs. TGFB1 +GEM			
		**KRT18**	**−1.75959393686352**	**2.39315053186199E−91**
		**KRT8**	**−1.0309653765519**	**3.31022239139809E−46**
		**KRT19**	**−1.18554997104256**	**1.31856311025676E−34**
	TGFB1 vs. TGFB1 +GEM	no KRTs		
	GEM vs. TGFB1	**KRT18**	**−1.97154126982277**	**4.57774984067198E−146**
		**KRT8**	**−1.01654120726071**	**1.61919218704203E−60**
		**KRT19**	**−1.23272812083581**	**8.91150688036906E−48**
			**log2FoldChange**	**padj**
**DEGs Pseudobulk**	CTRL vs. TGFB1	**KRT8**	**−2.06666742066075**	**2.16748959143921E−39**
		**KRT18**	**−3.14488659745008**	**0**
		KRT32	−4.38357197167485	0.002484488551607
		**KRT19**	**−2.1807008699708**	**1.05086173652133E−194**
		KRT16	1.59414708698507	0.023788516440228
		KRT17	5.62492959249514	7.43546636168209E−05
	CTRL vs. TGFB1 +GEM	KRT80	−1.31925531797876	0.031018945671626
		**KRT8**	**−2.13781498291764**	**9.12146450046993E−121**
		**KRT18**	**−2.75580890266229**	**7.21565468096864E−48**
		KRT32	−2.63029875411644	0.04323116180282
		**KRT19**	**−2.1756240120821**	**1.53243298042775E−181**
		KRT17	5.17372931952058	0.000459541942597
	TGFB1 vs. TGFB1 +GEM	no KRTs		

a using Seurat’s FindMarkers function with Wilcoxon Rank Sum test and pseudobulk analysis using DESeq2, respectively, for the different comparisons

Taken together, our results demonstrate that ML can be used as quality control to assess the reliability of gene expression differences identified by standard methods. The analysis of the top 10 differentially regulated genes using the STRING network analysis tool, which utilizes experimental and literature-based protein-interaction databases, helps to correlate findings to functional networks. We observe the greatest differences between the unstimulated control samples and the TGFB1-stimulated samples. Upon TGFB1 stimulation, several cytokeratins are downregulated, indicating a further shift to an EMT phenotype. The malignant transformation by TGFB1 could be demonstrated by the induction of more functionally invasive cells across the basement membrane within the SISmuc matrix. The comparison between TGFB1-stimulated cells and those that are also treated with GEM shows upregulation of genes related to the cell cycle, indicating a resistant phenotype. The resulting DEGs were also further validated by a novel learning approach, which is described in detail in the mathematical validation section in the Supplementary Material “Objective Mathematical Validation”. Validating the information content of a set of genes instead of analyzing the expression of each gene separately can reveal additional findings, as also rules can be encoded in models that describe the relation between gene expression and phenotypes such as “sensitive to treatment if Gene A is low expressed and Gene B highly expressed or Gene A is highly expressed and Gene B is low expressed, and resistant else”, which would not be detected in case of considering each gene separately.

We could not detect great differences in gene expression between untreated control cells and GEM-treated cells, even though the tumor cell number was decreased to 75 %. Therefore, we hypothesized that most of the untreated control cells might already possess intrinsic characteristics that predict future treatment resistance, while only a comparatively small number of cells would be affected by GEM treatment. Thus, we applied a new mathematical approach to predict possible GEM-resistance characteristics in untreated cells. Detailed information on the mathematical reasoning can be found in the Methods section.

### 3.2 Mathematical approach to predicting GEM-resistant cell populations

**Fig. 10:**
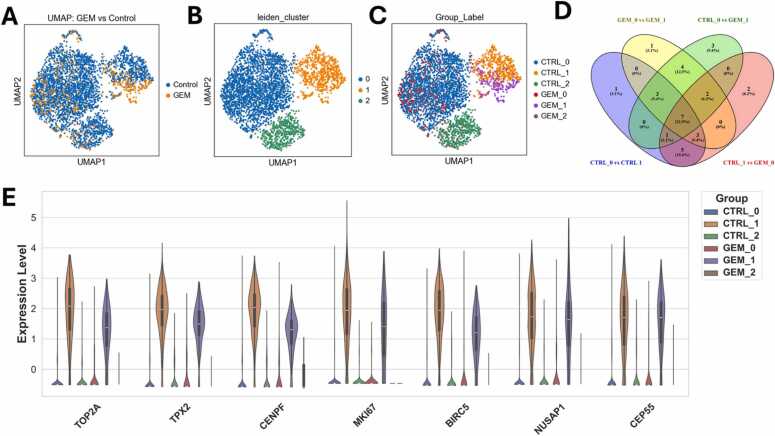
Comparing untreated control cells and GEM-treated cells. **(A)** UMAP visualization of untreated control cells (blue, two biological replicates, resulting in 3152 single cells after quality control and filtering) and GEM-treated cells (orange, one sample containing 392 single cells after quality control and filtering). **(B)** Leiden clustering of untreated control cells and GEM-treated cells results in three Leiden clusters. **(C)** All three Leiden clusters contain both untreated control cells and GEM-treated cells (e.g., for Leiden cluster 0 CTRL_0 (blue) and GEM_0 (red)). **(D)** Comparing the differences in gene expression between the cells of Leiden cluster 0 (CTRL_0 and GEM_0) and the cells of Leiden cluster 1 (CTRL_1 and GEM_1) indicates seven differentially expressed genes between the two clusters. **(E)** Visualization of the gene expression levels of the differentially expressed genes in the respective groups as violin plots.

Although the laboratory experiments indicated that approximately 25 % of the cells responded to GEM treatment, as shown in Fig. 1, the analysis of untreated control cells and GEM-treated cells indicated a relatively high similarity between the two groups (Fig. 10). Even if the control group consists of responding and non-responding cells, the laboratory experiments show that the responder group is small compared to the non-responder group, whose gene expression might be similar to that of the GEM-treated and surviving cells. Consequently, the control group is dominated by the non-responding cells. Therefore, it is challenging for ML methods to distinguish between cells treated with GEM and those in the control group. In addition, it might be misleading to associate cluster-specific differentially expressed genes with resistance, as the separation could also be due to other factors and might reflect other, possibly unknown, differences rather than GEM-resistance. Therefore, we aimed to retrospectively predict treatment responsiveness in untreated PANC-1 cells by implementing a novel transcriptome-based classification approach grounded in cosine distance and supervised machine learning, to possibly identify an inner structure of the control cell cluster with respect to the phenotype of GEM-treatment sensitivity.

As an initial step, we performed Leiden clustering (res 0.095) to identify potential subpopulations in an unsupervised manner. This standard approach detects transcriptionally similar groups of cells and is widely used to explore heterogeneity in single-cell data.

The UMAP plot (Fig. 10A) shows the high transcriptional similarity between the untreated control and GEM-treated cells. Leiden clustering identified three clusters (Fig. 10B) with clusters 0 (blue) and 2 (green) appearing similar, while cluster 1 (orange) forms a distinct group. All clusters include both control and GEM-treated cells (Fig. 10C), indicating that treatment alone does not drive the clustering. However, comparing the top 20 MI genes that carry the most information for classifying cells according to two clusters (Fig. 10D, Supplementary Material) reveals transcriptional differences. Seven MI genes (*TOP2A*, *TPX2*, *CENPF*, *MKI67*, *BIRC5*, *NUSAP1*, and *CEP55*) show higher expression in both control (CTRL_1) and GEM-treated (GEM_1) cells of Leiden cluster 1, compared to cells in other clusters (Fig. 10E). These patterns suggest the existence of a transcriptionally distinct subpopulation enriched genes associated with proliferation. The respective genes (*TOP2A*, *TPX2*, *CENPF*, *MKI67*, *BIRC5*, *NUSAP1*, and *CEP55*) are more highly expressed in both control cells (CTRL_1) and GEM-treated cells (GEM_1) of Leiden cluster 1 than in the other Leiden clusters.

These genes are either known proliferation markers (*TOP2A*, *MKI67*, and *BIRC5*) or associated with the cell cycle (*TPX2*, *CENPF*, *NUSAP1*, and *CEP55*) [60].

While the elevated expression of proliferation- and cell cycle-associated genes in Leiden cluster 1 might suggest a more aggressive phenotype, it is equally possible that this cluster simply reflects a group of cells in active cell division at the time of sequencing. Therefore, based solely on Leiden clustering, it remains unclear whether these transcriptional differences predict treatment outcomes.

We asked whether transcriptional heterogeneity among untreated control cells might already reflect latent differences in treatment susceptibility. To explore this possibility, we retrospectively compared each untreated cell to GEM-treated cells using cosine distance in PCA space. By computing cosine distances in PCA-reduced space, we measured how transcriptionally similar each untreated cell is to the GEM-treated population. This allowed us to stratify untreated cells based on their molecular resemblance to the treatment state, without prior knowledge of their actual response.

Subsequently, all cells of the control group were ranked according to their minimal cosine distance among all cosine distances to each of the GEM-treated cells. The cell with the highest minimal cosine distance to GEM-treated cells was ranked as most dissimilar, and the cell with the lowest minimal cosine distance was ranked as most similar. This novel approach is not part of standard single-cell analysis pipelines and is intended as a novel exploratory approach to assess the potential treatment relevance of transcriptional variation prior to drug exposure.

To determine the predictive value of the ranking, we divided the cells into two groups: predicted-sensitive cells and predicted-resistant cells. Since the GEM-treated cells had survived GEM treatment, we assumed that untreated control cells that show similarities in gene expression to GEM-treated cells (low minimal cosine distance to GEM-treated cells) have a high chance of surviving GEM treatment. Therefore, we labeled these cells as predicted-resistant. On the other hand, a high minimal cosine distance to GEM-treated cells indicates that the respective cells are different from GEM-treated cells. Thus, GEM-treatment might affect these cells more and could possibly kill them. Hence, the cells with the highest minimal cosine distance to GEM-treated cells were labeled predicted-sensitive.

At first, we used the supervised machine learning algorithm, which is based on earlier studies, Caliskan et al. [24] and Rasbach et al. [25], and is described in detail in Caliskan et al. [26] to calculate the prediction accuracy of a subpopulation.

**Fig. 11:**
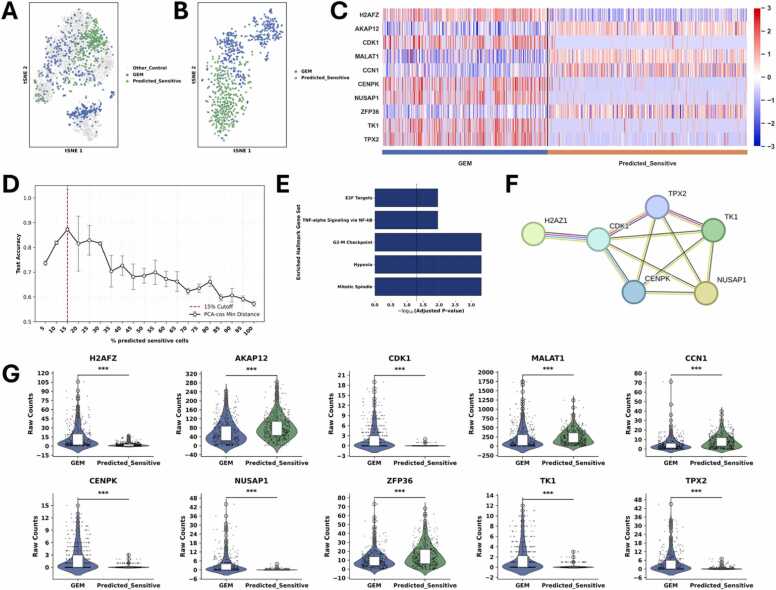
Evaluation of class separation between predicted-sensitive control cells and GEM-treated cells via supervised machine learning and visualization of the expression of the top-ranked MI genes. **(A)** t-SNE visualization of GEM-treated cells (blue), control cells (gray), and predicted-sensitive control cells (green) with 15 % of all untreated control cells labeled as predicted-sensitive (and the remaining 85 % labeled as predicted-resistant). The percentage for predicted-sensitive cells was chosen according to the high prediction accuracy for this percentage of GEM-dissimilar cells (Fig. 11D). **(B)** The t-SNE visualization of GEM-treated cells (blue) and predicted-sensitive cells (the top 15 % of GEM-dissimilar cells) shows a clear separation between the two groups. **(C)** Heatmap of the ten top-ranked MI genes. **(D)** Predicted accuracy for different percentages of predicted-sensitive cells. The highest predictive accuracy is achieved with 15 % of all untreated control cells labeled as predicted-sensitive (and the remaining 85 % labeled as predicted-resistant). **(E)** Hallmark enrichment analysis based on the ten top-ranked MI genes (via gseapy (v1.0.5)). **(F)** Visualization of the STRING analysis of the interconnected top-ranked MI genes (standard medium confidence of 0.4, with disconnected nodes hidden, using STRING version 12.0) **(G)** Violin plot visualizations of the expression of the top-ranked MI genes in GEM-treated cells (blue) compared to predicted-sensitive cells (green).

We observed that setting the proportion of predicted-sensitive cells to 15 % – corresponding to the 15 % of control cells most dissimilar to GEM-treated cells – led to the highest prediction accuracy of approximately 89 % (Fig. 11D). Increasing the fraction of cells labeled as predicted-sensitive (e.g., labeling 50 % of the cells as predicted-sensitive) decreased prediction performance (Fig. 11D). We remark that given the uncertainties of the accuracies, we observe a plateau of accuracy in Fig. 11D that drops after approximately 20–25 %. Higher percentages of predicted-sensitive cells result in a lower prediction accuracy. This indicates that cells that are among the 25 % top-ranked “most GEM-dissimilar cells” contain information that helps the ML algorithm to distinguish these cells from GEM-treated cells. Considering more cells (e.g., 40 % or 50 % of the top-ranked GEM-dissimilar cells) results in information that is less helpful for the algorithm to distinguish between predicted-sensitive and GEM-treated cells. This results in a lower prediction accuracy. In our case, this threshold is reached when cells that are not among the top-ranked 25 % GEM-dissimilar cells are considered as predicted-sensitive, which appears to prevent the algorithm from correctly identifying predicted-sensitive and GEM-treated cells. This finding corresponds well with the experimental finding of approximately 25 % cells dying under treatment, see Fig. 1, and suggests a cutoff value between 15 % and 25 % for subsequent analyses (see Fig. 11D).

To assess whether transcriptional similarity to the GEM-treated state corresponds to previously identified transcriptomic subgroups, we selected the 15 % of the untreated control cells that were ranked as most dissimilar to GEM-treated cells (predicted-sensitive, i.e., the highest minimal cosine distance, highlighted in Fig. 11A, which also resulted in the highest prediction accuracy, see Fig. 11D). We use the assumption that high separation accuracy coincides with the difference in the gene expression profile with regard to responsive and resistant cells. While the control cells (gray in Fig. 11A) show no distinct differences in clustering from GEM-treated cells (blue in Fig. 11A), the predicted-sensitive control cells (green in Fig. 11A and in Fig. 11B) can now be clearly separated from the GEM-treated cells (blue in Fig. 11A and Fig. 11B).

Visualizing only the predicted-sensitive cells and the GEM-treated cells results in two clusters: One comprising all predicted-sensitive cells (green in Fig. 11B) and another comprising the GEM-treated cells (blue in Fig. 11B). As described in detail in Rasbach et al. (2024) [25] and Caliskan et al. (2025) [26], the ML algorithm uses mutual information to identify the genes that are most relevant for distinguishing between two groups, with the most relevant genes being the top-ranked MI genes. The ten top-ranked MI genes are visualized as a heatmap in Fig. 11C. Hallmark enrichment analysis demonstrates that the top-ranked MI genes are also associated with the cell cycle (Fig. 11E). Additionally, six of the MI genes are also highly interconnected in a STRING analysis (Fig. 11F, STRING version 12.0 [52], [53], [54], with a standard medium confidence of 0.4 and disconnected nodes hidden). Compared to GEM-treated cells, predicted-sensitive control cells express significantly less *H2AFZ*, *CDK1*, *CENPK*, *NUSAP1*, *TK1*, and *TPX2* (Fig. 11G) and show a higher expression of *AKAP12*, *MALAT1*, *CCN1,* and *ZFP36*.

To see the results of other distance measures, please see the Supplementary Material “Extended Results”.

Finally, we performed survival analyses for the MI genes resulting from the comparison between predicted-sensitive control cells and GEM-treated cells using the Kaplan-Meier Plotter web-tool [55], [57], [58] (access date 29th September 2025, available at https://server2.kmplot.com/pancreas). The plots are available in the Supplementary Material. High expression of several of the MI genes, including *CDK1*, *CENPK*, *TK1*, and *TPX2*, has been associated with a worse prognosis according to survival analysis.

## 4 Discussion

In this study, we used 3D PANC-1 SISmuc models reflecting the high resistance to GEM observed in the clinic [8], which was further enhanced by TGFB1. Our laboratory data indicate niche-specific responses to GEM, with only approximately 25 % of 3D cultured cells being affected by GEM treatment. To analyze the resulting data, we combined standard scRNA-seq analyses with an ML-based pre-analysis (gSELECT [26]), which confirmed the high similarity between cells with and without GEM treatment. Additionally, we utilized a complementary mathematical approach to assess group separability and identify possibly treatment-sensitive subpopulations in untreated PANC-1 cells.

TGFB1 stimulation was associated with the upregulation of EMT and TNF-α signaling via NF-κB compared to control cells without TGFB stimulation. Treatment with TGFB1 +GEM was not only associated with EMT and TNF-α signaling via NF-κB but also with “Glycolysis”. When comparing GEM-treated cells with and without TGFB1 stimulation, the cell-cycle-related hallmarks “G2-M Checkpoint” and “E2F Targets” were the only hallmarks associated with the differentially expressed genes. To analyze differentially expressed genes between untreated control cells and GEM-treated cells, we hypothesized that some PANC-1 cells might be less affected by GEM-treatment than others.

Using a mathematical approach, we stratified the control cells by similarity to GEM-survivors. This approach identified a smaller “predicted-sensitive” subgroup and a predominant “predicted-resistant” cell population, aligning differences in gene expression with the heterogeneous GEM response observed *in vitro*. The resulting MI genes are associated with hallmarks such as “E2F Targets”, “G2-M Checkpoint”, and “Mitotic Spindle”, which are relevant to the cell cycle. They include important cell cycle regulators such as *CDK1*, which is essential for G2/M phase transition and DNA repair [61], as well as *NUSAP1*, *TK1*, and *TPX2*.

TGFB1 stimulation induced EMT and mainly downregulated the expression of cytokeratins, involved in EMT, which is a feature related to the desmoplastic reaction and high-grade malignancy in PDAC.

### 4.1 Effect of TGFB1-stimulation on 3D-cultured PANC-1 cells

As traditional 2D methods cannot reproduce cellular interactions and the tumor microenvironment observed *in vivo* [11], [12], [13], we employed a SISmuc matrix [19], [29] to generate a 3D environment instead of cocultures with stromal cells such as fibroblasts or mesenchymal stromal cells [29]. The PANC-1 cell line was selected as a representative for PDAC cells due to its characteristic mutations in *KRAS*, *P53*, and *P16/CDKN2A* [22].

In our data, we observed a strong induction of EMT by TGFB1. PANC-1 cells expressed high levels of vimentin, consistent with a mesenchymal phenotype and literature [62], and previous reports have associated TGFB1-induced EMT in this cell line with increased invasiveness [63]. Our observations are therefore in line with the established role of TGFB1 as a potent inducer of EMT in PDAC, its key role in the tumor microenvironment, and its correlation with invasive capabilities and metastasis potential [64], [65], [66], [67]. This indicates that our model can capture aspects of *in vivo* conditions and may therefore serve as a useful tool for PDAC research [29], [68].

GEM treatment did not appear to alter EMT, as both TGFB1 and TGFB1 +GEM conditions resulted in MI genes associated with EMT. Furthermore, TNF-α signaling, which was also enriched in our MI gene analysis for the comparison between predicted-sensitive cells and GEM-treated cells, has been implicated in therapy resistance, tumor aggressiveness, and immune suppression [69]. Inhibition of TNF-α in combination with chemotherapy has been shown to partially reverse PDAC chemoresistance [70].

Using an antibody targeting numerous cytokeratins, we performed immunofluorescence staining of our PANC-1 tissue models. TGFB1 stimulation resulted in downregulation of cytokeratins in the stainings but vimentin (VIM) expression remained unchanged, which is also confirmed by our *in silico* analyses particularly the downregulation of cytokeratin 18 (KRT18). It is reported that depletion of KRT8/KRT18 does not affect expression levels of EMT markers such as VIM, E-cadherin, and N-cadherin [71]. The investigation of additional EMT markers (e.g., SNAI1, ZEB1, FN1) would further strengthen this observation. They will be included in future studies that further analyze the potential interactions between KRT18, EMT, and GEM-response observed in this study. Loss of KRT8/KRT18 expression has been associated with chemoresistance and metastasis in epithelial cancer [71]. In epithelial cancer cells, a stable knockdown of KRT8/KRT18, which are usually co-expressed, resulted in increased invasiveness and migration, indicating that KRT8/KRT18 expression can affect the phenotype of epithelial cancer cells [71]. In breast and prostate cancer, downregulation of KRT18 was associated with increased aggressiveness [72], [73]. For pancreatic cancer, the effect of KRT18 dysregulation has been controversially discussed in the literature. While some associate KRT18 with a shorter overall survival of pancreatic cancer patients [74], others have implicated low KRT18 expression with a more aggressive pancreatic cancer phenotype [75]. Additionally, KRT8, KRT18, and KRT19, which were all among the identified markers and DEGs for our comparisons between untreated or GEM-treated cells compared to cells treated with TGFB1 or TGFB1 and GEM, are known to influence cell death [76]. Therefore, further research regarding the role of KRT18 and the other cytokeratins in pancreatic cancer, preferably in 3D models, could be of great interest.

To further examine transcriptional changes associated with TGFB1 stimulation, we compared control cells with both TGFB1-stimulated cells and TGFB1 +GEM-treated cells. MI-analysis revealed a strong overlap between both comparisons, with seven of the ten top-ranked genes shared (*PMEPA1*, *SERPINE1*, *TGFBI*, *TPM1*, *SPOCK1*, *COL1A1*, and *KRT18*). Several of these genes are known to be dysregulated in pancreatic cancer and to interact with TGFB1, such as *PMEPA1* and *TPM1*. *PMEPA1* is involved in TGFB1 regulation and has been associated with the progression of pancreatic cancer [77]. Additionally, it has been reported that *PMEPA1* interference enhanced GEM-sensitivity in human pancreatic cancer cells [78]. TGFB1 can stimulate *TPM1* expression, and elevated TPM1 levels have been associated with a poor prognosis in PDAC [79]. A table summarizing the known functions of these MI-genes in pancreatic cancer and their potential effect on GEM-treatment, as well as their impact on clinical outcomes, is available in the Supplementary Material (Supplementary Table 1).

### 4.2 Effect of GEM-treatment on 3D-cultured PANC-1 cells

Peindl et al. (2022) highlight this 3D tissue model for Non-small cell lung cancer (NSCLC) as a more realistic tumor microenvironment characterized by homeostasis of tumor cell growth and altered expression patterns, reflecting chemoresistance and improving predictability compared to 2D and animal models [19]. However, it is important to note that the 3D model provides still only a limited microenvironment, excluding essential *in vivo* characteristics such as the systemic immune response, even though different immune therapeutic strategies can be tested, as done with CAR T cells or with bispecific antibodies [19], [80], [81], [82], [83].

Our analysis suggests changes in gene expression related to enhanced cell cycle progression after GEM-treatment in combination with TGFB1. Contrary to that, research has shown that GEM treatment inhibits tumor cell proliferation [84]. We cannot determine whether our observations are based on the selection of cells with a highly active cell cycle after GEM treatment or if the cell cycle is even induced by GEM treatment. However, our data would suggest that GEM-resistant cells might efficiently circumvent cell cycle arrest.

Looking at the clinic, most patients treated with GEM alone or in combination only get a marginal benefit in terms of survival when compared to other available chemotherapeutics, such as the combination of leucovorin and fluorouracil plus irinotecan and oxaliplatin (FOLFIRINOX) which results in a survival time nearly double than that observed with GEM [9], [10]. This disparity in findings reinforces the marked failure-rate of translating results from preclinical setups to clinical settings, suggesting that information regarding GEM treatment in *in vitro* and *in vivo* models is somehow biased and/or that these models do still not accurately represent the reality of PDAC drug response sufficiently well.

To find potential mechanistic reasons for the niche-specific response to GEM in the deeper crypt areas of our PDAC tissue model, which we never have observed before in our other models for NSCLC with targeted drugs [19], [20] or in colorectal cancer (CRC) models with chemotherapeutics [21], we applied a new mathematical approach to compare control cells with GEM treatment surviving cells. One limitation of this study is the limited cell number in the cell cluster treated with GEM. Since histological sections show no reduction of cell numbers in TGFB1-treated samples and only a small reduction of cell numbers in samples without TGFB1 that were treated with GEM alone, we assume that GEM-treated cells might not survive the extraction procedure from the tissue matrix as well, which might be due to being pre-damaged by the chemotherapeutic drug.

### 4.3 Mathematical approach for predicting GEM-sensitivity and clinical relevance

Cell-cycle pathways such as “E2F Targets” and “G2-M Checkpoint” are linked to the basal-like state and aggressiveness of PDAC [85]. Moreover, these pathways, as well as the mitotic spindle, have been associated with *TP53* codon 273 mutations [85], which are present in PANC-1 cells [86]. These observations suggest that alterations in cell-cycle control contribute to PDAC plasticity and therapy resistance.

Using Leiden clustering, both control and GEM-treated cells separated into three groups, one of which showed high expression of proliferation- and cell cycle-associated genes, such as the proliferation markers *TOP2A*, *MKI67*, and *BIRC5* [60], as well as genes which are associated with the mitotic spindle, such as *TPX2*, *CENPF*, *NUSAP1*, and *CEP55* [60]. These markers, linked to different cell-cycle phases, might be associated with more aggressive phenotypes. For instance, *BIRC5* (survivin) is overexpressed in multiple cancers, including PDAC, and has been proposed as a diagnostic and therapeutic target [87]. However, their presence in both control and GEM-treated samples suggests that they do not specifically capture GEM effects. Consistent with this, standard ML analysis revealed only modest differences between untreated and GEM-treated cells.

To address this limitation, we applied a mathematical transcriptome-based classification approach, which is presented here as a proof-of-concept. Control cells with low transcriptional similarity to GEM-treated survivors were classified as “predicted-sensitive”, whereas those with high similarity were classified as “predicted-resistant”. The highest classification accuracy (approximately 89 %) was achieved when approximately 15 % of control cells were defined as predicted-sensitive, with performance plateauing around 20–25 %. This proportion matched our experimental observation that approximately 75 % of cells survived GEM treatment in 3D culture, supporting the plausibility of the approach. PDAC is well known for both intrinsic and acquired chemoresistance [88]. In patient-derived xenograft models, GEM resistance was associated with pathogenic *TP53* mutations and upregulated “Glycolysis” [88]. Notably, PANC-1 cells harbor a *TP53* mutation [86], and “Glycolysis” was among the top enriched pathways in the MI gene analysis of TGFB1 +GEM-treated cells. This suggests the biological relevance of the identified MI genes.

The possible existence of intrinsic GEM-resistant cancer cell subpopulations has also been studied in laboratory experiments, such as in experiments by Principe et al. (2022), who observed potentially GEM-resistant clusters in their analyses of GEM-treated PANC-1 cells [89]. Furthermore, Ungefroren et al. [90] and Färber et al. [91] reported heterogeneity among single cell-derived clonal cultures of PANC-1 cells [90], [91].

Färber et al. created single-cell-derived cell lines (SCDCLs) from the basal-like PDAC cell line PANC-1 [91]. Some of these SCDCLs exhibited more spindle-shaped cells, which indicate a more mesenchymal phenotype, while others grew in forms indicating a more epithelial phenotype [91]. Additionally, the SCDCLs showed a heterogeneous response to GEM treatment, with some SCDCLs being more resistant to GEM treatment (survival rate of 0.8) and others being more sensitive to GEM treatment (survival rate of 0.66) [91]. This supports our assumption that predicted-sensitive and resistant subpopulations coexist within the control population. Importantly, their pathway enrichment results overlapped with ours: three Hallmark gene sets (“E2F Targets”, “G2-M Checkpoint”, and “Hypoxia”) identified in our comparison between predicted-sensitive and GEM-treated cells were also enriched in Färber et al.'s analysis of resistant and sensitive SCDCLs [91]. Future analyses could also combine their approach of generating SCDCLs with our *in silico* approach, predicting their treatment response *in silico*, and validating the prediction *in vitro* using the SCDCLs.

Interestingly, many of the top-ranked MI genes distinguishing predicted-sensitive from GEM-treated cells (e.g., *CDK1*, *H2AFZ*, *CENPK*, *NUSAP1*, *TPX2*, and *MALAT1*) have also previously been linked to clinical outcome or drug response in PDAC. For instance, CDK1 downregulation in predicted-sensitive cells aligns with reports associating high CDK1 expression with poor prognosis and GEM resistance [92], [93], while depletion of H2AFZ isoforms has been observed to sensitize pancreatic ductal adenocarcinoma cells to GEM, and to reduce tumor size in a mouse xenograft model [94]. A complete overview of all ten MI genes for the comparison between “predicted-sensitive” cells and GEM-treated cells and their reported clinical associations is provided in Supplementary Table 2.

However, the genes identified here should be regarded as candidate genes potentially involved in GEM response, based on *in silico* analyses. Their relevance requires further validation. An additional validation strategy is performing a survival analysis. Using the Kaplan-Meier Plotter tool [55], [57], [58], we confirmed the potential prognostic value of several MI genes. Future studies should include functional validation, for example by knockdown or overexpression of candidate genes in PDAC cell lines, followed by assessment of GEM response and other standard therapies *in vitro* and in preclinical models. Moreover, time-resolved single-cell sequencing after GEM exposure could help to capture dynamic changes in gene expression and identify early versus late response mechanisms. Timely resolved data is also helpful to generate gene interaction networks [95] that allow studying causal mechanisms between treatment and gene interaction. Furthermore, such gene interaction networks facilitate separating genes correlating with phenotypic difference from the ones that are causally related to observed changes.

Additionally, the relatively small number of GEM-treated cells and the low sample number might limit the interpretability and generalizability of the findings. A recent publication by Breitenbach and Dandekar [96] addresses the critical question of how the data size is related to the uncertainties of results and to estimate a sufficient size of the data. In future work, we plan to extend the gSELECT framework [26] to address the question of data sufficiency in single-cell analyses systematically. Building on recent methodological advances [96], this will involve defining criteria for when datasets are large enough such that uncertainties of results fall below a predefined threshold, similar to approaches used in bootstrap-based uncertainty quantification [96]. This consideration is particularly relevant given the limited number of GEM-treated cells in our dataset. Another limitation of our study is the predominant focus on *in silico* approaches. To mitigate this limitation, we performed survival analyses for all genes of interest (available in the Supplementary Material) to analyze their biological relevance in pancreatic cancer.

Further experiments could include different GEM treatment durations to better evaluate potentially GEM-related changes in gene expression.

However, since our novel approach has indicated several genes that might be involved in GEM-sensitivity and could contribute to a better understanding of GEM-resistance, we would like to share the results of the analyses to draw attention to these genes and to the method by which we identified a potentially sensitive subgroup in the group of control cells, thus more information about its sub or fine structure.

### 4.4 Conclusion

This study shows that the 3D SISmuc model can capture tumor-specific processes such as EMT, invasion, and drug resistance in PANC-1 cells. Compared with 2D culture, GEM treatment had limited effects in 3D culture, whereas TGFB1 induced EMT and nearly complete resistance. Using transcriptome-based classification, we were able to distinguish “predicted-sensitive” from “predicted-resistant” subpopulations among control cells. Comparing these “predicted-sensitive” cells to GEM-treated cells resulted in several candidate genes linked to cell-cycle regulation, consistent with known mechanisms of GEM resistance. These findings point to molecular factors that may contribute to GEM response and highlight the value of combining 3D models with computational analyses to better characterize treatment sensitivity in PDAC.

## CRediT authorship contribution statement

**Tim Breitenbach:** Writing – review & editing, Writing – original draft, Supervision, Conceptualization. **Jesús Guillermo Nieves Pereira:** Writing – review & editing, Data curation. **Thomas Dandekar:** Writing – review & editing, Supervision. **Gudrun Dandekar:** Writing – review & editing, Writing – original draft, Supervision. **Aylin Caliskan:** Writing – review & editing, Writing – original draft, Formal analysis, Data curation. **Samantha A. W. Crouch:** Writing – review & editing, Writing – original draft, Formal analysis.

## Funding, Acknowledgement

This research was funded by the 10.13039/501100002745Bavarian Research Foundation (GD, TD: project: AZ-1365–18, JNP DOK-184–20). TD thanks also DFG (270563345 /GRK2157 3DInfect; funding 3DTissue culture analysis) and Land Bavaria for support (contribution to DFG Project number 324392634 – TRR 221/INF, funding GvL/cancer treatment) and (492620490 /SFB1583 DECIDE/INF modelling cellular decision processes). SAWC thanks Hans-Böckler-Stiftung for support. We (TD, GD, JNP) thank the Single-Cell Center at the Helmholtz Institute for RNA-based Infection Research (HIRI, University Würzburg) for funding of this study with a single cell seed grant (#19_2021_11).

The funders had no role in deciding about the research conducted, the decision to publish and did not interfere in any way with our research. There was no writing assistance, no use of generative AI.

## Declaration of Competing Interest

The authors declare there are no conflicts of interest, neither personal nor financial ones.

## Footnotes

Appendix A Supplementary data associated with this article can be found in the online version at doi:10.1016/j.csbj.2025.10.032.
