# Supplementary material for: Mathematical strategies for predicting resistant subpopulations from scRNAseq data of a PANC-1 3D tissue model: Insight into gemcitabine resistance and TGFB1-induced invasion and EMT
Source: Comput Struct Biotechnol J. 2025 Oct 16;27:4476–95. doi: 10.1016/j.csbj.2025.10.032 (PMC12593213; doi:10.1016/j.csbj.2025.10.032)
Supplement: Supplementary file 1 — Supplementary material [file mmc1.pdf]

Additional information on the resulting MI genes

Supplementary Table 1: The seven common MI genes of the ten top-ranked genes differentially expressed between control and TGFB1-stimulated cells and control and TGFB1+GEM-treated cells.

Gene	Known in Pancreatic Cancer? Known in Cancer?	In PANC-1	Effect on GEM-treatment?	Expression Impact on Outcome	Notable Literature Findings
PMEPA1 Prostate Transmembrane Protein, Androgen Induced 1	is involved in TGFB1 regulation and has been associated with the progression of pancreatic cancer [1],	**In PANC-1** Overexpression of PMEPA1 drives invasive capacity and cell viability of PANC-1 cells, knockdown suppressed invasive capacity and cell viability [1], In PANC-1 cells, PMEPA1 increases the proliferative potential via activation of the MAPK signaling pathway [1]	PMEPA1 interference enhanced GEM-sensitivity in human pancreatic cancer cells by activating PTEN/PI3K/AKT signaling [2].	Low PMEPA1 expression has been shown to be associated with disease-free survival [2] Overexpression of PMEPA1 correlates with worse clinical outcomes [1],	PMEPA1 has been reported to facilitate tumor growth via interacting with Smad2 and Smad3 [1], Involved in the progression of pancreatic cancer via MAPK signaling, and triggers deregulation of P53 in PANC-1 cells [1], High expression has been reported for several solid cancers, including hepatocellular carcinoma and breast carcinoma [1],
SERPINE1 Serpin Family E Member 1 (PAI-1)	SERPINE1 can be used as a marker to identify quasi-mesenchymal cells in PDAC [3].	In PANC-1 cultures, TGFB1 stimulation could induce a more extreme M phenotype, which is indicated by dysregulation	-	SERPINE1 encodes plasminogen activator inhibitor-1 (PAI-1), whose role in pancreatic cancer has	In esophageal cancer, SERPINE1 is among the most prognostic genes and is involved in invasion and metastasis [6]

		of several genes, including upregulation of SERPINE1 [4].		been controversially discussed in literature [5], high mRNA and protein levels of PAI-1 appear to be associated with a poor prognosis [5]	In oxaliplatin-treated cells of the colorectal cancer cell line HCT116, inhibition of SERPINE1 reversed chemoresistance and metastasis [7],<br>In breast cancer metastasis-associated lung endothelial cells, upregulation of SERPINE1 has been reported to promote cancer stem cell properties and to shield cancer cells from paclitaxel-induced apoptosis [8]<br>In triple-negative breast cancer, expression of SERPINE1 has been reported to correlate with patient's BMI and poor outcomes, and has been linked to radioresistance [9]
TGFBI<br>Transforming Growth Factor Beta Induced	It has been reported that TGFB1 can activate the PI3K-AKT pathway in pancreatic cancer cells, which then promotes EMT, resulting in perineural invasion[10]	Using PANC-1 derived circulating tumor cells (Panc-1-CTCs), Sato et al. (2018) identified *TGFBI* as a crucial gene for acquiring a malignant phenotype,	Targeting TGFBI in macrophages has been reported as a promising approach for enhancing the efficacy of pancreatic cancer chemotherapy [11]	TGFBI expression has been reported as upregulated in perineural invasion tissue and significantly correlates with a poor	TGFBI has been identified as key player in perineural invasion, a common pathological feature of pancreatic cancer [10]

	In murine pancreatic cancer tumors, TGFBI was identified as Tumor-associated macrophage (TAM)-producing factor and a macrophage-intrinsic regulator, which contributes to tumor growth [11]	which suggests TGFBI as a potential therapeutic target in PDAC [12]		prognosis in pancreatic cancer [10]	
TPM1 Tropomyosin 1 (Alpha)	TGFB1 stimulates Smad-dependent TPM1 expression [13], and in PDAC patients, elevated expression of TPM1 was associated with poor prognosis [14].<br>In drug-resistant pancreatic cancer cell lines, TPM1 expression is reduced [15]	PANC-1 cells showed defective proliferation after being exposed to microRNA-21 antisense oligonucleotides, which downregulate the oncogenic miRNA 21, which targets several genes, including TPM1 [16]	Co-delivery of microRNA-21 antisense oligonucleotides and GEM was more effective than both treatments alone, resulting in more cell apoptosis and a greater extend of growth inhibition in pancreatic cancer cells, which suggests that the combination of miR-21 gene silencing and GEM-treatment might be a promising treatment strategy [16]	Reduced expression of TPM1 in pancreatic cancer has been associated with worse survival [15]	TPM1 is one of the targets of miR-21, a microRNA that is significantly upregulated in pancreatic cancer [17, 18], in chemoresistant cancer cell lines, the miR-21-family is overexpressed, while the expression of TPM1 is significantly reduced [18]
SPOCK1 SPARC/Osteonectin, CWCV and Kazal-like Domains Proteoglycan 1	SPOCK1 has been identified as TGFB1-target in lung cancer, where it regulates the EMT of lung cancer cells [19]. TGFB1-stimulation increases the expression of SPOCK1, and increased SPOCK1 levels were associated with shorter	Knockdown of SPOCK1 significantly reduced the growth of PANC-1 cells [20]	In pancreatic cancer cells, ablation of SPOCK1 did not appear to result in enhanced efficiency of paclitaxel or GEM-treatment [21], however, since *SPOCK1* overexpression contributes to EMT, pancreatic cancer cell proliferation, and metastasis, *SPOCK1* has been	In PDAC patients, a higher expression of SPOCK1 correlated with shorter disease-free and overall survival time, while knockdown of SPOCK1 increased apoptosis in PDAC	In other tumor types than PDAC, where its expression appears to be stromal rather than epithelial, SPOCK1 appears to be mainly expressed in the epithelial fraction [21]

	disease-free survival in lung cancer, while silencing SPOCK1 inhibited lung cancer cell growth, invasion and colony formation *in vitro* [19].<br>In pancreatic cancer, SPOCK1 promotes metastasis via NF-κB-dependent EMT [20].		suggested as potential therapeutic target [20].	cells, which suggests its potential use as prognostic and therapeutic target [22]	
COL1A1<br>Collagen Type I Alpha 1 Chain	COL1A1 is a member of the collagen family and known to be involved in EMT and the regulation of cellular processes such as apoptosis, proliferation, and metastasis, and has been associated with cisplatin resistance [23]. Human pancreatic cancer cell lines have recently been reported to only express Col1α1, which is encoded by COL1A1, but do not express Col1α2 (encoded by COL1A2), while human fibroblasts produce both Col1 chains [24]. The Col1	bone morphogenetic protein1 (BMP1) has been reported to selectively suppress metastasis and tumor growth in cells that express high levels of COL1A1. In an analysis of different pancreatic cancer cell lines (BxPC, AsPC1, and PANC-1), the highest expression of COL1A1 and the largest tumor-suppressing effect of BMP1 has been observed in the PANC-1 cell line [26]	In pancreatic cancer cells, COL1A1 has been reported as involved in GEM resistance [27]	In pancreatic cancer, higher COL1A1 expression has been associated with a shorter overall survival time [25]	COL1A1 is linked to KRT18 via co-expression in several organisms according to the STRING database [28-30] and expressed by cancer-associated fibroblasts (CAFs), which only exist in tumor tissues [31].

	homotrimer of α1 chains resulting from COL1A2 Col1a2 gene suppression appears to be specific to cancer cells and has been reported to be oncogenic and to influence the tumor microenvironment [24]. In pancreatic cancer, type I collagen (Col I) exhibits both pro- and anti-tumoral effects [25]. The heterotrimeric Col I consisting of Col I (α1/α1/α2) has been reported to suppress tumor growth [25]. In contrast, homotrimeric Col I (α1/α1/α1), which is produced by tumor cells, promotes immune invasion and activates TGF-β/SMAD3 signaling, which drives EMT and substantially increases tumor cell migration in 3D cell culture models [25].				
KRT18<br>Keratin 18	In epithelial cancer cells, loss of K8/18 expression during EMT has been	In pancreatic cancer cells, KRT18 expression is downregulated, and Kim et	-	In TCGA cohorts, higher KRT18 expression was	Cook and Vanderhyden assessed four different cancer cell lines known to be

	associated with metastasis and chemoresistance [32]<br>High expression of KRT8 negatively impacts the prognosis of PDAC patients and is involved in migration and viability of PDAC cells [33]. Knockdown of KRT8 was shown to impair migration and proliferation and to induce apoptosis in PDAC and was associated with a slight downregulation of KRT18 [33].	al. (2014) have reported a more than 2-fold downregulation in PANC-1 cells [34]		associated with a worse prognosis [33].	capable of EMT by exposing them to EMT-inducing factors, generating data from 12 distinct EMT time course experiments. Among the genes that most frequently changed were the epithelial-associated keratins *KRT8*, *KRT18* and *KRT19*, which were downregulated, consistent with the loss of epithelial features [35].<br>However, the impact of KRT18 on survival and malignancy is controversially discussed in literature. In one study, overall survival analysis of KRT18 expression has indicated that pancreatic cancer patients with lower KRT18 expression had better survival, while high KRT18 expression was associated with a shorter overall survival [36]. On the other hand, low KRT18 expression has also been associated with a more

					aggressive phenotype in a study by Walsh et al. (2009), who reported that a highly invasive pancreatic cancer clone showed low KRT18 expression and that low KRT18 expression had been implicated with a more aggressive phenotype [37].

Supplementary Table 2: The ten top-ranked genes differentially expressed between predicted-sensitive cells (control cells with a high minimal cosine distance distribution to GEM-treated cells) and GEM-treated cells, and their implications in pancreatic cancer.

Gene	Known in Pancreatic Cancer? Known in Cancer?	In PANC-1	Effect on GEM-treatment?	Expression Impact on Outcome	Notable Literature Findings
H2AFZ<br>H2A.Z Variant Histone 1 (downregulated in predicted-sensitive (ps))	Highly expressed in pancreatic cancer cell lines and pancreatic cancer patients, overexpression of H2AZ1 has been reported to suppress senescence and chemosensitivity in pancreatic ductal adenocarcinoma [38]<br>upregulated in various cancers, including colorectal cancer and lung cancer, also elevated in cardiac hypertrophy [39]<br>upregulated in hepatocellular carcinoma and associated with tumor malignancy and poor prognosis [40]	Significantly upregulated in PANC-1 and other pancreatic cancer cell lines (e.g., Capan-1 and MiaPaCa-2) [38]	Depletion of H2A.Z isoforms has been observed to reduce tumor size *in vivo* (in a mouse xenograft model) and to sensitize pancreatic ductal adenocarcinoma cells to gemcitabine [38]<br>Knockdown of H2A.Z increases senescence by affecting the expression of genes involved in senescence [41]	Overexpression is associated with a poor prognosis in hepatocellular carcinoma [40]	A histone variant that is involved in processes such as DNA repair, transcriptional control, and regulation of centromeric heterochromatin [39]<br>In PDAC, H2A.Z depletion has been suggested to favor a senescent phenotype and reduced proliferation resulting from a G2/M phase arrest [38]. Additionally, Ávila-López et al. (2021) have reported that depletion of two H2A.Z genes in PDAC resulted in decreased expression of genes involved in cell cycle regulation, nucleoside metabolism, and apoptosis [38].
AKAP12<br>A-Kinase Anchoring Protein 12	Overexpression of *AKAP12* has been reported in various pancreatic cancer cell lines	Compared to other pancreatic cancer cell lines, PANC-1 showed high mRNA	-	Higher expression of AKAP12 mRNA was correlated with	*AKAP12* has been reported to correlate with tumorigenesis of some cancers, and as

(upregulated in ps)	and in patient samples compared to normal pancreatic RNA samples and *AKAP12* might be associated with a less aggressive pancreatic cancer phenotype [42]<br>In some anti-VEGF therapy-resistant cancers, including glioblastoma, colorectal cancer, and ovarian cancer, AKAP12 has been reported as upregulated and associated with dismal prognoses in patients being treated with anti-VEGF inhibitors [43]<br>In various cancers, AKAP12-regulated signaling pathways play an essential role in initiation and tumor progression, and it has been reported that AKAP12 can inhibit the cell cycle protein CDK1, which can prevent the growth of cancer cells [44].	expression of AKAP12 in qRT-PCR [42]		decreased metastasis and invasion scores and might be associated with less aggressive phenotypes of pancreatic ductal adenocarcinoma [42]	greatly associated with differential and angiogenesis of retinoblastoma and with metastasis of lung cancer, and appears to play a dual role in cancer, as high expression of *AKAP12* has been associated with both favorable and unfavorable prognoses in different cancer types (e.g., in colorectal cancer elevated *AKAP12* expression was associated with poor survival, while *AKAP12* indicated better prognoses in other cancers, such as kidney renal clear cell carcinoma, myeloid leukemia, and thyroid carcinoma [43] In various cancers, AKAP12-regulated signaling pathways play an essential role in initiation and tumor progression, and it has been reported that AKAP12 can inhibit the cell cycle protein CDK1, which can prevent the growth of cancer cells [44].

CDK1 Cyclin Dependent Kinase 1 (downregulated in ps)	In tumor cells of pancreatic ductal adenocarcinoma patients, CDK1 genes have been reported as significantly overexpressed, and overexpression of CDK1 is associated with more advanced PDAC stages and indicates poor survival rates [45]<br>Higher expression of CDK1 was associated with a poor prognosis in pancreatic cancer [46].	In GEM-resistant PANC-1 cells, high expression of CDK1 and altered cell cycle signaling pathway have been reported [47];	In pancreatic cancer tissue and GEM-resistant cells, high expression of *CDK1* has been observed [47]; fisetin has been reported to enhance the effect of gemcitabine by inhibiting the CDK1-STAT3 axis *in vitro* and *in vivo* [47];<br>A phase I study with patients with refractory solid tumors showed clinical benefit for the combination of a CDK1/2/4/5 inhibitor (milciclib) and gemcitabine, the results included disease stabilization over 6 months for a pancreatic cancer patient [45];<br>Comparable results have also been reported in a phase II study with hepatocellular carcinoma patients; indicated some efficacy [45];<br>In a clinical phase I study of another CDK1/2/4/5 inhibitor (PHA-848125AC) some efficacy has been reported for patients with advanced solid tumors, and pancreatic cancer patients showed stable disease for 10 months [45];	High *CKD1* levels have been associated with poor overall survival and poor disease-free survival [47];<br>In preclinical models, targeting CDK1 has shown promising results for the treatment of pancreatic ductal adenocarcinoma by inducing cell cycle arrest in G2/M-phase and stimulating apoptosis; additionally, PDAC cancer stem cells have been reported as sensitive to CDK1 inhibition [45] and increased expression of *CDK1* and *CCNA2* have been associated with a poor prognosis in pancreatic cancer [46]	Post-translational modifications of CDK1–STAT3 signaling are involved in maintaining cancer stemness of pancreatic ductal adenocarcinoma, and targeting the CDK1-STAT3 axis with inhibitors appears to be a potential therapeutic strategy [47];

MALAT1 Metastasis Associated Lung Adenocarcinoma Transcript 1 (upregulated in ps)	In pancreatic ductal adenocarcinoma, MALAT1 expression is increased, and MALAT1 upregulates cellular autophagy in pancreatic cancer cell lines [48], it also facilitates cell growth, migration and invasion in pancreatic cancer [49] Highly expressed in several malignancies, including pancreatic cancer, prostate cancer, hepatocellular carcinoma [48]	Relative expression of MALAT1 in PANC-1 cells is higher than in human pancreatic duct epithelial cells, Aspc-1, and SW1990, while the relative MALAT1 expression of Bxpc-3 and CFPAC is higher than in PANC-1 [48]	In non-small cell lung cancer, MALAT1 was implicated in GEM-resistance via the MALAT1/miR-27a-5p/PBOV1 axis [50]; forced level of MALAT1 was reported to enhance GEM-resistance of A549 cells, while silencing MALAT1 weakened this impact [50] In pancreatic cancer, upregulation of the long noncoding RNA (lncRNA) MALAT1 has been associated with modulating GEM resistance [51]. Additionally, it has also been reported that GEM treatment reduces the expression of MALAT1 in PANC-1 cells [52].	Elevated expression of MALAT1 has been associated with poorer prognoses in pancreatic ductal adenocarcinoma [48] and unfavorable prognosis in pancreatic cancer [53]	MALAT 1 is also referred to as nuclear-enriched abundant transcript 2 (NEAT2) and was originally identified as a sign of metastasis in lung cancer [48]. Knockdown of MALAT1 has been reported to inhibit cell cycle progression and to impair tumor cell migration and invasion [54], knock-down of MALAT1 also reduces proliferation and cell growth in pancreatic tumors *in vitro* and *in vivo* and attenuates the protein expression of KRAS [54],
CCN1 Cellular Communication Network Factor 1 (upregulated in ps)	*CCN1* is known to play a critical role in pancreatic carcinogenesis by inducing EMT and stemness, and it has been shown to regulate the Sonic Hedgehog signaling pathway, which is associated with progression of pancreatic ductal adenocarcinoma and a poor prognosis [55], *CCN1*	Lack of CCN1 expression in PANC-1 cells reduces differentiation and decreases GEM-sensitivity, which has been reported to be due to a reduced expression of genes involved in transport and metabolism of GEM [57]		Both CCN1 mRNA and protein were detected in early precursor lesions of PDAC, and their expression intensifies during disease progression, [55]; overexpression of CCN1 in pancreatic cancer and its precursor lesions have been shown to	*CCN1* has been proposed as promising target in pancreatic cancer cells as it appears to affect two critical signaling cascades, Sonic-Hedgehog signaling and the integrin αvβ3-Notch1 signaling pathway, which appears to be critical for CCN1-induced Sonic Hedgehog expression in pancreatic cancer [55]

	expression is upregulated in pancreatic, ovarian, and prostate cancer, but downregulated in lung and gastric cancer [56]<br>While upregulation of CCN1 has been detected in early precursor lesions of PDAC and increases during PDAC progression [55], knockout of CCN1 has been reported to reduce cell differentiation [57].			promote proliferation, EMT and cancer cell migration [55];<br>PANC-1 cells with CCN1 knockout have been reported to show reduced differentiation and decreased GEM-sensitivity [57]	
CENPK<br>Centromere Protein K<br>(downregulated in ps)	*CENPK* is differentially expressed several cancers and high CENPK expression is associated with a worse prognosis, including in lung adenocarcinoma [58], *CENPK* is upregulated in lung cancer, triple-negative breast cancer, ovarian cancer, and hepatocarcinoma [59]	-	-	Upregulation of *CENPK* is associated with malignant progression in triple-negative breast cancer, ovarian cancer, and hepatocarcinoma; in hepatocarcinoma, depletion of CENPK decreased the expression of YAP1 and reduced invasion, migration and proliferation in HCC cells, this suggests that	In prostate cancer, a splice variant of *CENPK* has been associated with resistance to the CYP7 inhibitor Abiraterone [61]<br>CENPK knockdown was also associated with decreased expression of CCN1 and YAP1 [60]. YAP1, which is a key part of the Hippo signaling pathway, has been reported as essential for initiation, progression and metastasis of numerous cancer types [60].

				knockdown of CENPK can inhibit proliferation, migration and invasion of hepatocarcinoma cells by regulating YAP1, YAP1 in turn is involved in regulating EMT in various cancers, including pancreatic cancer [60]	In murine models, YAP1 activity is attenuated by the small molecule inhibitor verteporfin [60], which has been shown to result in photodynamic therapy-induced tumor necrosis in locally advanced pancreatic cancer [60, 62].<br>Downregulation of NUSAP1 and CENPK, both of which have been associated with YAP1 regulation [60, 63], which in turn is involved in regulating EMT in various cancers [60], also appears to be associated with a more favorable prognosis.
NUSAP1<br>Nucleolar And Spindle Associated Protein 1 (downregulated in ps)	Upregulation of *NUSAP1* has been associated with tumor development and poor prognosis in several cancers, including breast, prostate, and cervical cancer and oral squamous cell carcinoma [64];	In a comparison of five pancreatic cancer cell lines, PANC-1 cells exhibited the highest *NUSAP1* expression [64]	-	*NUSAP1* has been suggested as an essential hub gene in pancreatic cancer and novel target for treatment of pancreatic cancer and has been reported to promote tumor growth, migration	*NUSAP1* is highly expressed in tumor tissue of most cancer types, primarily in malignant and immune cells [63];<br>In stomach cancer, *NUSAP1* has been observed to promote tumorigenesis and cancer progression via stabilization of the YAP1 protein [63]; In a

	In PDAC, *NUSAP1* acts as an oncogene and *NUSAP1* is higher expressed in tumor tissues compared to normal tissues, and might be involved in the development of PDAC [64]			and invasion in PDAC [64],<br>High expression of *NUSAP1* was observed in pancreatic ductal adenocarcinoma tissue and is associated with poor survival [64]	variety of cancers, including kidney cancer, lung adenocarcinoma, and pancreatic cancer, high expression of *NUSAP1* has been identified as risk factor in prognosis and tumorigenesis [63]<br>Downregulation of NUSAP1 and CENPK, both of which have been associated with YAP1 regulation [60, 63], which in turn is involved in regulating EMT in various cancers [60], also appears to be associated with a more favorable prognosis.
ZFP36<br>ZFP36 Ring Finger Protein<br>(upregulated in ps)	In non-small cell lung cancer, loss of ZFP36 caused upregulation of Barh-like homeobox 1 (BARX1), further promoting proliferation, migration and invasion of non-small cell lung cancer cells [65]; in various cancers, including hepatocellular carcinoma, breast,	In PANC-1 cells and AsPC-1 cells, ZFP36 overexpression has been reported to inhibit cell growth and colony formation compared with controls [67]	ZPF36 is downregulated by miR-29a in pancreatic cancer, which increases the expression of EMT-markers and pro-inflammatory factors. This suggests an oncogenic role for miR-29a due to downregulating ZFP36. Additionally, miR-29a is involved the response of pancreatic cancer cells to GEM-treatment by activating the Wnt/β-	Low expression of the tumor suppressor ZFP36 predicts poor patient outcome and reduced survival rate in pancreatic cancer, while overexpression of ZFP36 decreases proliferation and increases apoptosis	Also known as Zinc Finger Protein 36, Tristetraprolin, TTP and TIS11 [67];

	colorectal, and pancreatic cancer, ZFP36, which has been described as tumor suppressor, is downregulated or lost [65];<br>Downregulation of ZFP36 in pancreatic cancer has been associated with increased inflammation and EMT [66].		catenin pathway in pancreatic cancer cell lines and inducing GEM-resistance [66]	in pancreatic cancer cells [67];	
TK1<br>Thymidine Kinase 1 (downregulated in ps)	Overexpression of *TK1* has been associated with progression of pancreatic cancer and poor prognosis [68];<br>Expression patterns of the TK1 protein have been reported as potential clinicopathological predictive markers in a variety of cancers, including breast, lung, kidney, and gastrointestinal cancer [68]	Compared to a normal human pancreatic ductal cell line (hTERT-HPNE), TK1 is higher expressed in PDAC cell lines like SW1990, MiaPaCa-2, BxPc-3, CFPAC-1, and PANC-1, with a relatively high expression of *TK1* in PANC-1 and CFPAC-1 [68]	In lung cancer patients, a significant increase of TK1 activity was observed after the first and second cycle of platinum-based therapy combined with gemcitabine [69]	Knockdown of *TK1* could suppress cancer cell proliferation in pancreatic cancer, while overexpression was associated with shorter disease free survival time and overall survival time [68]; *TK1* has been suggested as promising therapeutic target in pancreatic ductal adenocarcinoma [68]	*TK1* , which is known as proliferation marker in breast cancer and a marker for predicting reoccurrence of prostate cancer, has been suggested as prognostic and therapeutic target in pancreatic cancer; in mitotic and malignant cells, *TK1* is overexpressed, in quiescent cells it is absent [68]<br>Downregulation of *TK1* has been observed to inhibit cell proliferation *in vitro* and *in vivo* [68]
TPX2<br>TPX2 Microtubule Nucleation Factor	TPX2 has been suggested as potential oncogene due to its overexpression in non-small-	The PANC-1 cell line expresses high levels of TPX2 protein [70]	TPX2 knockdown has been shown to sensitize PANC-1 and MIA PaCa-2 to paclitaxel treatment, but no	TPX2 inhibition has been reported to lead to PANC-1 cells failing to	*TPX2* has been validated as potential therapeutic target in pancreatic cancer [70]; During

(downregulated in ps)	cell lung cancer and pancreatic ductal adenocarcinoma [70];		synergistic effect for the combination of silencing TPX2 via TPX2 siRNA and gemcitabine was observed [70]	progress through mitosis; Additionally, TPX2 inhibition might cause apoptosis [70];<br>In patients with advanced pancreatic ductal adenocarcinoma, high TPX2 expression indicated significantly shorter progression-free survival and overall survival times, and high TPX2 expression has been suggested as negative predictor of gemcitabine-based palliative and adjuvant chemotherapy in PDAC [71]	mitosis, TPX2 interacts with downstream partners including the Aurora A kinase [70];

Transcriptome-Based Retrospective Stratification of Untreated Cells

To assess whether untreated cells already differ in their transcriptional predisposition to GEM-treatment, we developed a retrospective, unsupervised stratification strategy based on cell similarity. Specifically, we asked: **Can untreated control cells be subdivided according to their transcriptional resemblance to GEM-exposed cells, prior to any drug exposure, and can this be predictive of treatment response?**

We computed pairwise distances between each untreated control cell and all GEM-treated cells in a low-dimensional representation of the transcriptomic space.

Based on these distances, control cells were ranked and stratified: those most similar to GEM-treated cells were labeled *predicted-resistant*, whereas those most dissimilar were labeled *predicted-sensitive*. This yields a pseudo-supervised classification that may capture latent predispositions to therapeutic sensitivity or resistance, based on the hypothesis that control cells with dissimilar gene expression to GEM-treated cells are more likely to be affected by GEM-treatment. In contrast, control cells with gene expression similar to GEM-treated cells would be less affected by GEM-treatment and thus be able to survive GEM-treatment (similar to the GEM-treated cells, which were the surviving cells sequenced after GEM-treatment).

To explore the robustness of this approach, we systematically compared four distance-based strategies defined by two components:

1. **The dimensionality reduction space** used to represent global transcriptomic structure:
    - **PCA** (Principal Component Analysis) to preserve as much of the variability as possible while reducing the dimensionality of the dataset [72]. PCA reduces the dimensionality of data with a high number of interrelated variables by transforming the data into uncorrelated variables called principal components, with the first few components retaining most of the original variation [73]
    - **t-SNE** (t-distributed Stochastic Neighbor Embedding), a method which was designed to retain the local structure of high-dimensional data [74, 75] to emphasize local transcriptional neighborhoods.
2. **The distance metric** used to quantify similarity between cells:
    - **Cosine distance**, which allows measuring similarity or difference between sets based on the angle between two vectors, rather than the magnitude of the vectors [76], thus focusing on direction, while being less sensitive to magnitude variations.
    - **Euclidean distance**, which reflects the magnitude of differences between two gene expression profiles [77] and emphasizes absolute expression-level changes across genes.

This resulted in four classification strategies:

- **PCA + cosine (mean/min)**
- **t-SNE + Euclidean (mean/min)**

For each method, we tested both the minimum distance (i.e., the closest GEM cell) and the mean distance (i.e., average across all GEM cells) as decision heuristics.

To capture the transcriptional proximity of untreated cells to the GEM-induced state, we systematically explored different combinations of dimensionality reduction techniques and distance metrics. Principal Component Analysis (PCA) was employed due to its widespread use in transcriptomics [78] and its strength in preserving global structure [78] while attenuating noise. By projecting cells into a variance-maximizing linear space [72], PCA ensures that large-scale, biologically meaningful transcriptional shifts dominate the resulting similarity structure. In contrast, t-distributed Stochastic Neighbor Embedding (t-SNE) was used to highlight local topological relationships [74], offering sensitivity to finer substructures and transitional states that may be obscured in PCA space. However, this gain in resolution comes at the expense of global interpretability, as t-SNE is known for nor representing the global structure of the data accurately [75], due to the non-linear and stochastic nature of the embedding.

In parallel, we compared two distance metrics: cosine and Euclidean. Cosine distance is scale-invariant and emphasizes directional similarity in gene expression patterns [76], making it well-suited for capturing relative transcriptional programs independent of magnitude. Euclidean distance, by contrast, is sensitive to absolute differences in expression values and emphasizes magnitude-based dissimilarities [77], which can be informative when large expression shifts underpin biological divergence.

Each method combination was evaluated using both the minimum and the mean distance between individual control and GEM-treated cells. The minimum distance heuristic prioritizes proximity to the closest GEM-treated cell, potentially highlighting outlier-driven similarity. Mean distance, in contrast, reflects the average similarity or dissimilarity of a control cell to the entire GEM-treated cell population.

Together, these design choices enable a systematic and nuanced exploration of whether baseline transcriptomic similarity to GEM-treated cells can serve as a surrogate for latent treatment sensitivity or resistance. Importantly, by evaluating prediction accuracy across a spectrum of stratification thresholds (5-100%), we assess how the granularity of class definition influences predictive performance and variance.

We hypothesize that cells most dissimilar to the GEM-induced state – designated as *predicted-sensitive* – may undergo the most pronounced transcriptional transitions upon treatment and might be more sensitive to GEM-treatment. Conversely, cells with high baseline similarity to

GEM-treated cells – *predicted-resistant* – may already exhibit features of the drug-adapted state, thus exhibiting a reduced response or even possibly resistance. This framework offers a novel, label-free strategy to infer pre-treatment sensitivity from transcriptomic context alone and may serve as a hypothesis-generating tool for uncovering early determinants of therapeutic response.

Extended Methods

Stratification Based on Transcriptomic Similarity

To identify transcriptionally predisposed subpopulations prior to GEM treatment, we implemented an unsupervised framework that quantifies similarity between untreated (control) and GEM-treated single cells in reduced-dimensional expression space.

Dimensionality Reduction

We first projected all cells into lower dimensions using either Principal Component Analysis (PCA) or t-distributed Stochastic Neighbor Embedding (t-SNE):

```
sc.pp.normalize_total(adata, target_sum=1e4)
sc.pp.log1p(adata)
sc.pp.highly_variable_genes(adata, n_top_genes=2000)
sc.pp.scale(adata, max_value=10)

sc.tl.pca(adata, n_comps=30, svd_solver="arpack")  # PCA projection
sc.tl.tsne(adata, n_pcs=30)                         # t-SNE projection
```

PCA was selected for its ability to capture global transcriptional variance [72], while t-SNE was used to explore local substructures [74], which might be potentially related to transitional states or rare subpopulations.

Similarity Metrics

We computed pairwise distances between all control and GEM-treated cells using both cosine and Euclidean distance metrics:

```
from sklearn.metrics.pairwise import cosine_distances, euclidean_distances

ctrl_idx = adata.obs["condition"] == "Control"
gem_idx  = adata.obs["condition"] == "GEM"

X_ctrl = adata.obsm["X_pca"][ctrl_idx]
X_gem  = adata.obsm["X_pca"][gem_idx]

dist_cos = cosine_distances(X_ctrl, X_gem)       # Cosine
dist_euc = euclidean_distances(X_ctrl, X_gem)    # Euclidean
```

Cosine distance is scale-invariant and emphasizes expression directionality, whereas Euclidean distance reflects absolute magnitude differences. For each control cell, we derived two heuristics:

- **Minimum distance** to any GEM cell (potentially capturing the closest GEM-treated cell for the respective control cell)
- **Mean distance** across all GEM cells (taking all GEM-treated cells into account, resulting in an average value)

```
min_dist_cos = dist_cos.min(axis=1)
mean_dist_cos = dist_cos.mean(axis=1)
```

Cell Stratification by Distance Thresholds

Cells were stratified into *predicted-sensitive* and *predicted-resistant* subgroups based on quantiles (5%-100%) of their distance scores. For instance, for 5%, the 5% of control cells with the least similarity to GEM-treated cells were labeled as *predicted-sensitive*, while the remaining 95% of control cells were labeled as *predicted-resistant*. This approach allowed systematic evaluation of how the stringency of the stratification affects downstream predictive performance.

```
# Example for top 10% most distant (Predicted_Sensitive)
nsel = int(0.10 * len(mean_dist_cos))
rank = np.argsort(mean_dist_cos)
```

```
sens_cells = adata.obs_names[ctrl_idx][rank[-nsel:]]
res_cells  = adata.obs_names[ctrl_idx][rank[:nsel]]
```

The resulting groups were labeled as GEM (for GEM-treated cells), *predicted-sensitive* (for the subset of control cells with the least similarities to GEM-treated cells (e.g., 5%), and *predicted-resistant* (for the remaining control cells with higher similarity to GEM-treated cells, e.g., the remaining 95%) follows:

```
labels = pd.Series("GEM", index=adata.obs_names)
labels[sens_cells] = "Predicted_Sensitive"
labels[res_cells]  = "Predicted_Resistant"

adata.obs["Resistance_Class"] = labels.astype("category")
```

Rationale

- **PCA + cosine distance** yields interpretable, stable projections that prioritize directional similarity.
- **t-SNE + Euclidean distance** may reveal finer expression programs, but with increased variability and potential distortion.
- **Minimum distance** emphasizes nearest GEM-like states (but risks sensitivity to outliers).
- **Mean distance** provides more robust, population-averaged similarity metrics.

This framework allows unsupervised, hypothesis-generating classification of pre-treatment cells based on their transcriptional resemblance to GEM response, offering a way to identify latent sensitivity patterns.

Evaluation of Predictive Class Separation

We then evaluated the biological relevance of these predicted groups using supervised classification. Following Caliskan et al. (2023) [80] and Rasbach et al. (2024) [81], we trained a binary classifier to distinguish predicted-sensitive control cells from actual GEM-treated cells across varying thresholds (5%-100%). The model's performance, assessed by balanced accuracy, served as an objective measure of how well the stratification reflects true biological differences.

The genes identified during these analyses are based on mutual information (MI genes) and might be of interest as potential prognostic markers or therapeutic targets.

Objective Mathematical Validation

In order to better distinguish the cell groups classified as similar by the previously used standard method, we added a further validation step to our analysis process. In the next paragraph, we explain the mathematical backbone and important assumptions for this. The objective of our performed mathematical validation of identified genes describing characteristic differences in phenotypes or cell fate development is the following. Usually, mathematical methods used to extract genes whose expressions are related to the differences in the phenotype, or the cell fate development, rely on assumptions. These assumptions are not always fulfilled. In our case, we assumed that GEM-treatment, TGFB1-stimulation, and the combination of TGFB1_GEM, all affect PANC-1 cells and their gene expression. Additionally, we assumed that the most relevant differences in gene expression, the most relevant MI-genes, which were identified using mutual information, would be sufficient to distinguish between the different conditions, e.g., control and TGFB1- stimulated cells, resulting in a high balanced accuracy for the machine-learning classification.

As our results show, previous assumptions on gene expression, e.g., based on literature research or based on a hypothesis, can be misleading. While machine-learning analysis comparing control cells and TGFB1 or TGFB1_GEM-treatment resulted in very high balanced accuracies of over 95% when analyzing all genes (Figure 3C and Figure 3G), the machine learning approach was not able to reliably distinguish between control and GEM-treated cells (Figure 3E). However, relying on these results without further validation might also lead to wrong conclusions, and consequently, laboratory experiments or clinical trials might fail.

Therefore, we added a further validation step: We aim at verifying computational analysis results with a second method that is independent of the used analysis methods to test if, based on these results, the characteristic features or the development of the cell fate can correctly be described.

For this purpose, we train an ML model based on the analyzed genes to predict the characteristic differences or cell fates accordingly. In our case, we used the ten top-ranked MI genes of the respective analyses for the comparison between control and TGFB1 and the comparison between control and TGFB1_GEM. If this works with sufficient accuracy, we can be sure that the expression pattern of the selected genes contains enough information and the method we used to extract the corresponding genes was successful.

For the procedure, we split the single-cell data into a training and a test dataset. We train the model on all available genes that have a non-constant expression pattern over all cells. The purpose is to have an estimation of the information content with regard to describing the characteristic features. The accuracy difference on the test set between an ML model trained on all genes and only on the selected genes should not be too large because this might mean that information is somehow lost, and the essential genes for the characteristic difference or cell fate of interest might not be captured by the analyzed genes. In addition, to analyze what is a large difference, a third model is trained on randomly selected genes where the number of genes equals the number of the specifically selected genes, in our examples ten random genes. This is the accuracy possible due to redundancy in the expression pattern in the gene set. Due to randomness in the training process, the procedure is done several times, and the mean value and standard deviation can be taken to evaluate the model accuracy of the three different models and thus the information content of the selected genes. This procedure is described in more detail in Caliskan et al. (2023) [80]. The python script was taken from https://github.com/AC-PHD/Seurat_PFA_pipeline/tree/main/06_PFA/03_Validate_PFA_Results to perform the corresponding experiments. As the results in Figure 4A (control and TGFB1) and Figure 6A (control and TGFB1_GEM) show, performing the analyses with only the ten most relevant MI genes resulted in high balanced accuracies of over 95%.

A further advantage of this procedure is that we can systematically reduce the number of selected genes, e.g., according to a ranking from the analysis method, and balance the size of selected genes and accuracy, in other words, the complexity of the explanation and its accuracy. For instance, it is possible to perform the same analyses with only the five top-ranked MI genes. If the prediction accuracy remains high, the machine-learning algorithm can correctly predict the condition with only five MI genes.

We remark that a model failure, e.g., a low accuracy, does not mean that the selected genes are not working well because it might be that the chosen model cannot describe the functional dependencies between the gene expression and the characteristic differences or the cell fate well. However, if all methods provide consistent results, we have confident evidence that our analyses worked fine.

Besides visualizing the balanced accuracies when analyzing all genes, we also visualized the balanced accuracies for two additional gene selection scenarios: (1) using selected genes (here, the ten most relevant genes based on mutual information analysis) (2) an equal number of random genes selected from the non-constant features, e.g., ten random genes.

For the comparison between control and TGFB1, the balanced accuracy plot using all non-constant genes (Figure 3A) indicates a consistently high performance for differentiating between TGFB1-stimulated PANC-1 cells and untreated PANC-1 cells. Even considering only ten genes (the ten top-ranked MI genes for the comparison, Figure 4A) results in a high prediction accuracy.

The test performance for GEM treated cells is significantly lower and more variable, even when using all genes (Figure 3E), which might indicate a less distinct gene expression signature in response to GEM treatment or a more heterogenous response, which would make an accurate classification challenging. This observation is further supported by the t-SNE analysis in Figure 3D, where a distinction between control and GEM treatment groups is not apparent.

GEM-treated cells do not form a distinct cluster. The overlap between untreated control cells and GEM treated cells in the Figure 3D suggests that the transcriptional changes induced by GEM are either less pronounced in the cells that survive or more heterogeneous across the cell population that was measured, which might be an endpoint of the cell development, or no change was induced at all by the treatment at least in the cells that remain from GEM administration to the measurement. In contrast, TGFB1 stimulated cells form a distinct cluster that is clearly distinguishable from untreated control cells, which corroborates the high balanced accuracy using all non-constant genes. These findings indicate possible differences between both treatments and might indicate GEM-resistance. Therefore, we developed a mathematical approach testing the above-mentioned hypothesis that only a small number of control cells are affected by GEM-treatment, while most of the cells possess characteristics related to future treatment resistance.

Extended Results

Retrospective stratification approach

To investigate whether untreated cells exhibit transcriptional features indicative of their future response to GEM treatment, we implemented a retrospective stratification approach based on transcriptional similarity. In the following, we demonstrate two combinations of dimensionality reduction (PCA or t-SNE) and distance calculation (cosine distance or Euclidean distance) for both minimal and mean distance.

Specifically, we calculated the distance between each untreated control cell and the GEM-treated population using four combinations of dimensionality reduction and distance metrics: (1) PCA with mean cosine distance, (2) PCA with minimal cosine distance, (3) t-SNE with minimal Euclidean distance, and (4) t-SNE with mean Euclidean distance.

For each combination, we ranked control cells according to their distance from GEM-treated cells and labeled the top x% (e.g., 5%, 10%, ...) as *predicted-sensitive*, representing the control cells most dissimilar to the GEM-treated cells. Figure S 1 shows the classification accuracy across varying sensitivity thresholds (5% to 100%) for each method.

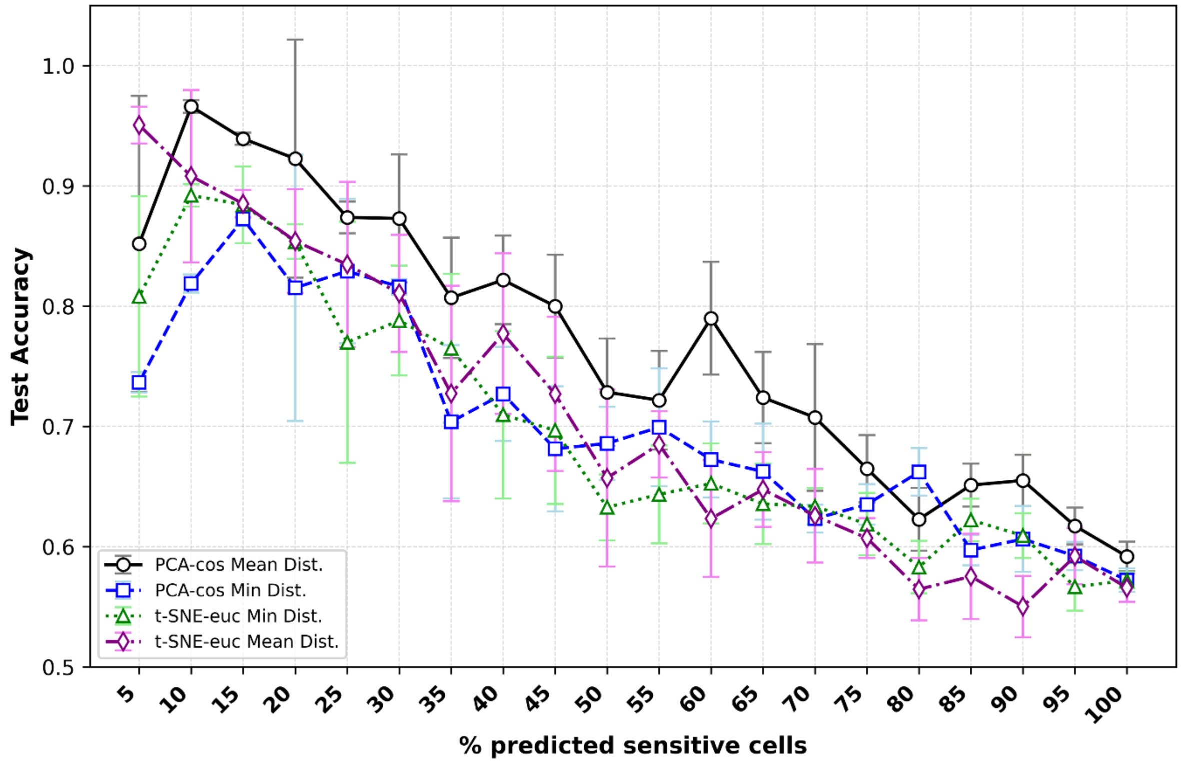

Figure S 1: Accuracy across stratification thresholds and methods. *Balanced accuracy (y-axis) across thresholds of predicted sensitivity (x-axis, in %, referring to the percentage of control cells labeled as predicted-sensitive) using the four above-mentioned distance-based stratification methods. Error bars indicate standard deviation. The PCA-cosine (mean, black line) method consistently achieves the highest accuracy and lowest variance, followed by PCA-cosine (min, blue line). Both t-SNE-Euclidean variants (green line for min distance, purple line for mean distance) show lower and less stable performance.*

While the PCA-cosine (mean) strategy consistently yielded the highest classification accuracy, prediction accuracy alone does not guarantee biological interpretability. Causally related genes that can be well biologically interpreted do not necessarily provide the highest accuracy. There can be genes whose expression very well correlates with the corresponding phenotype. However, a well working causal model that explains the phenotypic differences and changing the corresponding genes influences the phenotypes should have a high accuracy in predicting the phenotypes. Consequently, the genes that provide the causal relations sought might be under the

models that provide a high accuracy. To better understand the biological relevance of each stratification method, we next investigated which genes drive the separation between *predicted-sensitive* and GEM-treated cells.

To this end, we computed the mutual information (MI) between gene expression and class labels (GEM vs. Predicted-Sensitive) for each gene and ranked them accordingly. For each method and threshold, the top MI genes were extracted to identify features that most strongly distinguish the classes.

PCA-Cosine-Mean Distance Stratification (10%)

To assess the impact of distance calculation on transcriptional stratification, we applied a cosine distance-based labeling in PCA space using mean instead of minimal distances. Specifically, we calculated the average cosine distance of each control cell to all GEM-treated cells in PCA space and labeled the top 10% most dissimilar control cells as *predicted-sensitive*. This threshold was chosen because it yielded the highest classification accuracy (see Supplementary Figure S 1).

In contrast to the minimal distance approach presented in the main manuscript, mean distance yields a more conservative classification by averaging over the entire GEM population. This can reduce sensitivity to GEM-outlier cells but might influence the effect of substructures.

Despite its overall performance, gene-level analysis based on mutual information revealed less distinct marker genes compared to the cosine-minimal strategy. Several top-ranked genes lacked clear biological interpretability. While some of the genes (HF2AZ, CCN1, AKAP12, ZFP36, and MALAT1) were also among the top-ranked MI genes using the cosine-minimal strategy, other genes, such as NEAT1, were only identified by the cosine-mean strategy (Figure S 2).

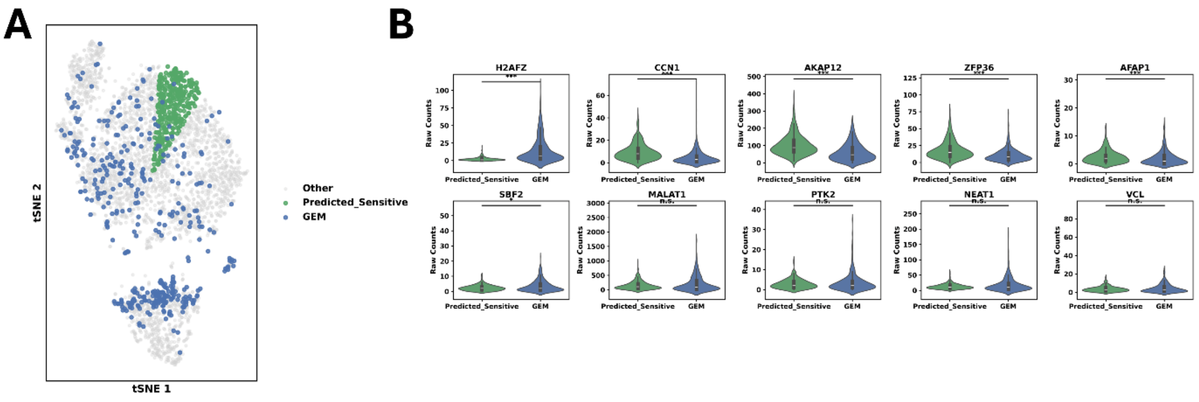

Figure S 2 PCA-Cosine-Mean Distance (10%). (A) *t-SNE visualization of GEM-treated cells (blue), the 10% of the control cells with the highest dissimilarities to GEM-treated cells (green), and the remaining control cells (gray).*

(B) Violin plots of the top 10 mutual-information-ranked genes distinguishing predicted-sensitive cells vs GEM-treated cells.

t-SNE-Euclidean-Minimal Distance Stratification (10%)

To complement our main PCA-based stratification, we additionally evaluated distance-based labeling using t-SNE with Euclidean distance. Specifically, we selected the 10% of control cells with the highest minimal Euclidean distance to GEM-treated cells in t-SNE space. This threshold was chosen as it yielded the highest classification accuracy in our benchmarking (Figure S 1), and all GEM-treated cells were retained as the reference group.

Although this approach achieved reasonable separation in the classifier, subsequent gene-level analysis revealed limitations. Using mutual information, we ranked genes by their ability to distinguish the *predicted-sensitive* group from GEM-treated cells. However, as visualized in Figure S 3B, these top 10 genes showed biologically less interpretable patterns compared to PCA-cosine-based methods. The functional relevance of the top-ranked genes remained ambiguous, although some of the top-ranked genes are also among the MI genes identified using one or both of the PCA-cosine-based methods (AKAP12, CCN1, and VCL), which might indicate their biological importance.

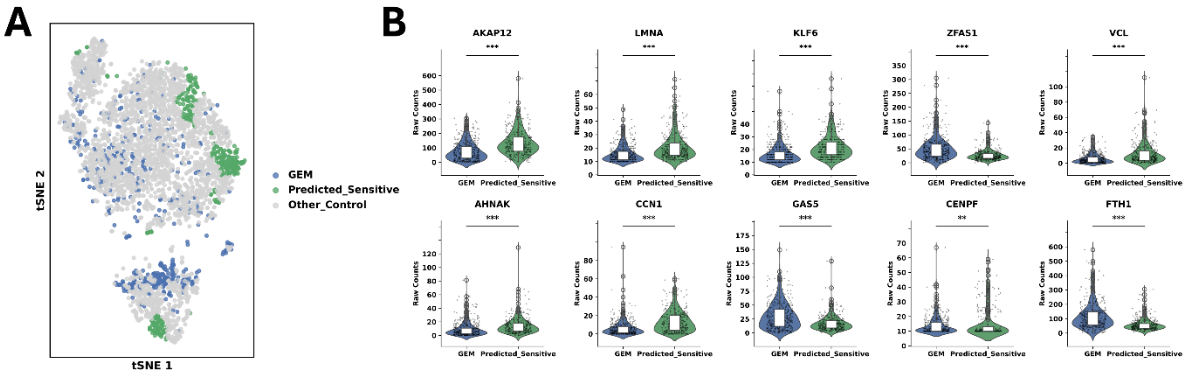

Figure S 3: Gene-level evaluation of predicted sensitivity based on t-SNE and Euclidean distance. (A) *Cells visualized in t-SNE space. The 10% of control cells with the highest minimal Euclidean distance to GEM-treated cells (blue) are labeled as predicted-sensitive (green); the remaining controls are visualized in gray.* ***(B)*** *Violin plots showing raw expression levels of the ten top-ranked genes with the highest mutual information (MI) between predicted-sensitive and GEM groups.*

t-SNE-Euclidean-Mean Distance

As an alternative to the minimum-distance heuristic, we also evaluated mean Euclidean distances in t-SNE space to stratify predicted sensitivity. According to the balanced test accuracy across the different thresholds (Figure S 1), we selected the top 5% of control cells with the highest average distance to all GEM-treated cells. This threshold was chosen because it yielded the highest classification accuracy (see Figure S 1).

Although this method resulted in technically valid group separation at the classifier level, interpretation of the gene-level signal was less conclusive. In Figure S 4B, we visualized the ten top-ranked genes ranked by mutual information between GEM-treated and predicted-sensitive cells. Also, the predicted-sensitive cells primarily clustered in the upper region of the t-SNE space (Figure S 4A), which might reflect local density effects rather than a distinct biological subpopulation. Interestingly, this approach also identified MI genes previously identified using PCA-cosine-based approaches (ZFP36 and H2AFZ).

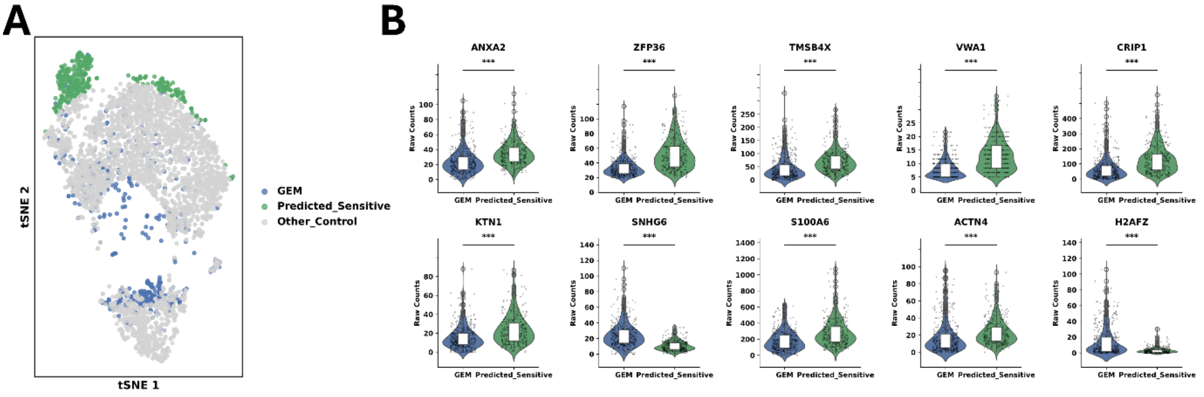

Figure S 4: t-SNE-Euclidean-Mean Distance-based labeling (5% threshold). (A) t-SNE embedding of all cells, with GEM-treated (blue), predicted-sensitive (green), and other control cells (gray). The 5% of control cells with the highest average Euclidean distance to all GEM cells were labeled as predicted-sensitive. (B) Violin plots showing the top 10 genes (raw counts) ranked by mutual information between GEM and predicted-sensitive cells

Supplementary Figures – Survival Analysis

Survival analyses of the ten top-ranked mutual information (MI) genes for the comparison between control and TGFB1-stimulated cells

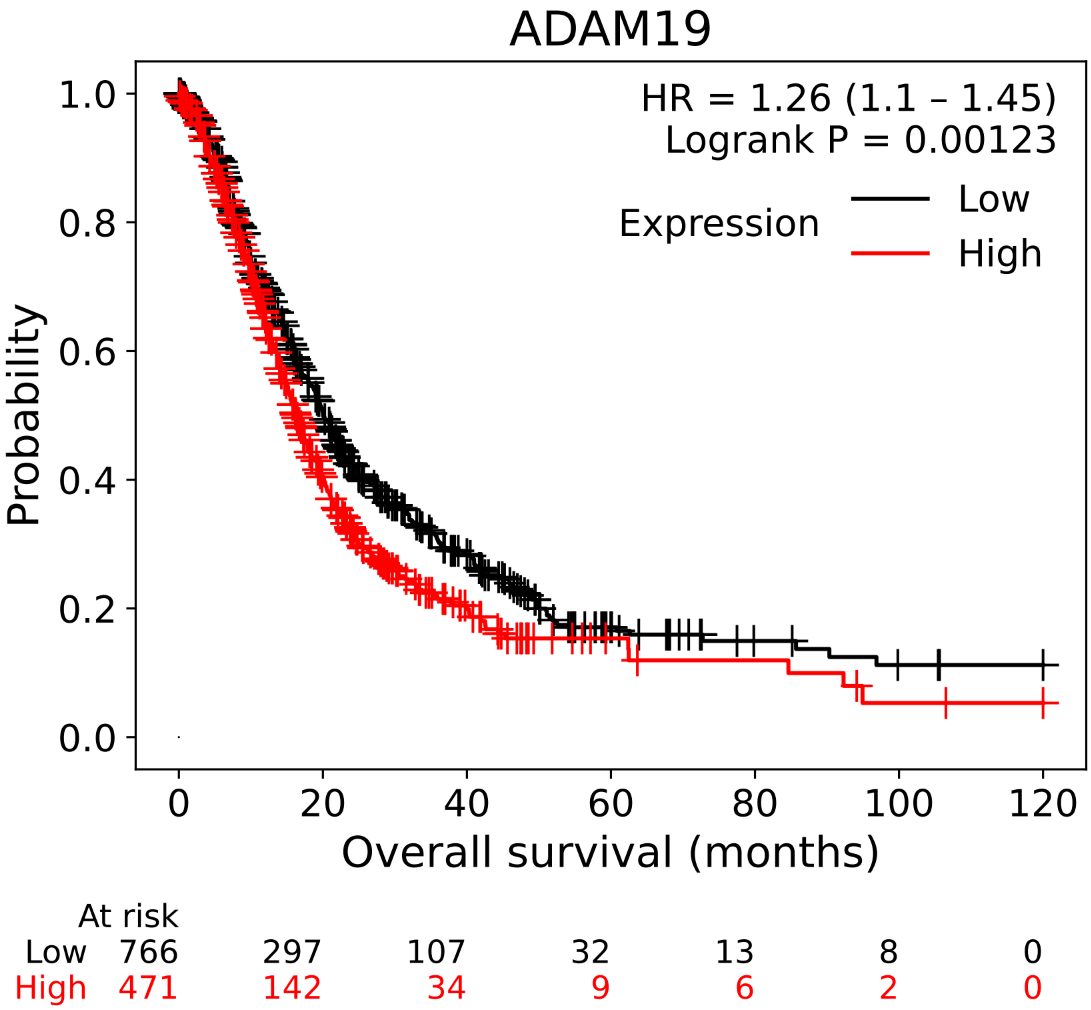

Figure S 5: Survival analysis for ADAM19, which is an MI gene in the comparison between control and TGFB1-stimulated cells. HR > 1: High expression associated with worse overall survival (OS), HR < 1: High expression associated with better OS, Log-rank P < 0.05 considered statistically significant.

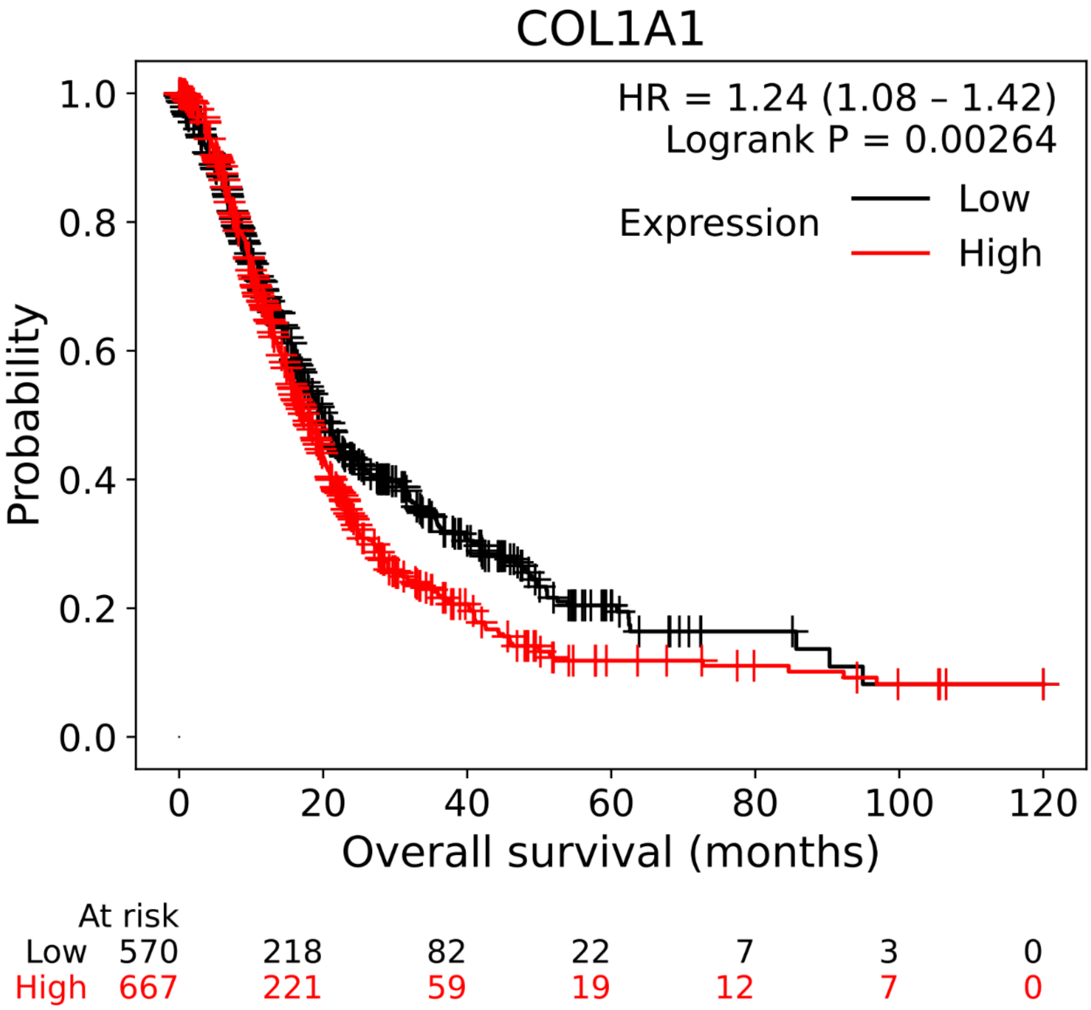

Figure S 6: Survival analysis for COL1A1, which is an MI gene in the comparison between control and TGFB1-stimulated cells. HR > 1: High expression associated with worse overall survival (OS), HR < 1: High expression associated with better OS, Log-rank P < 0.05 considered statistically significant.

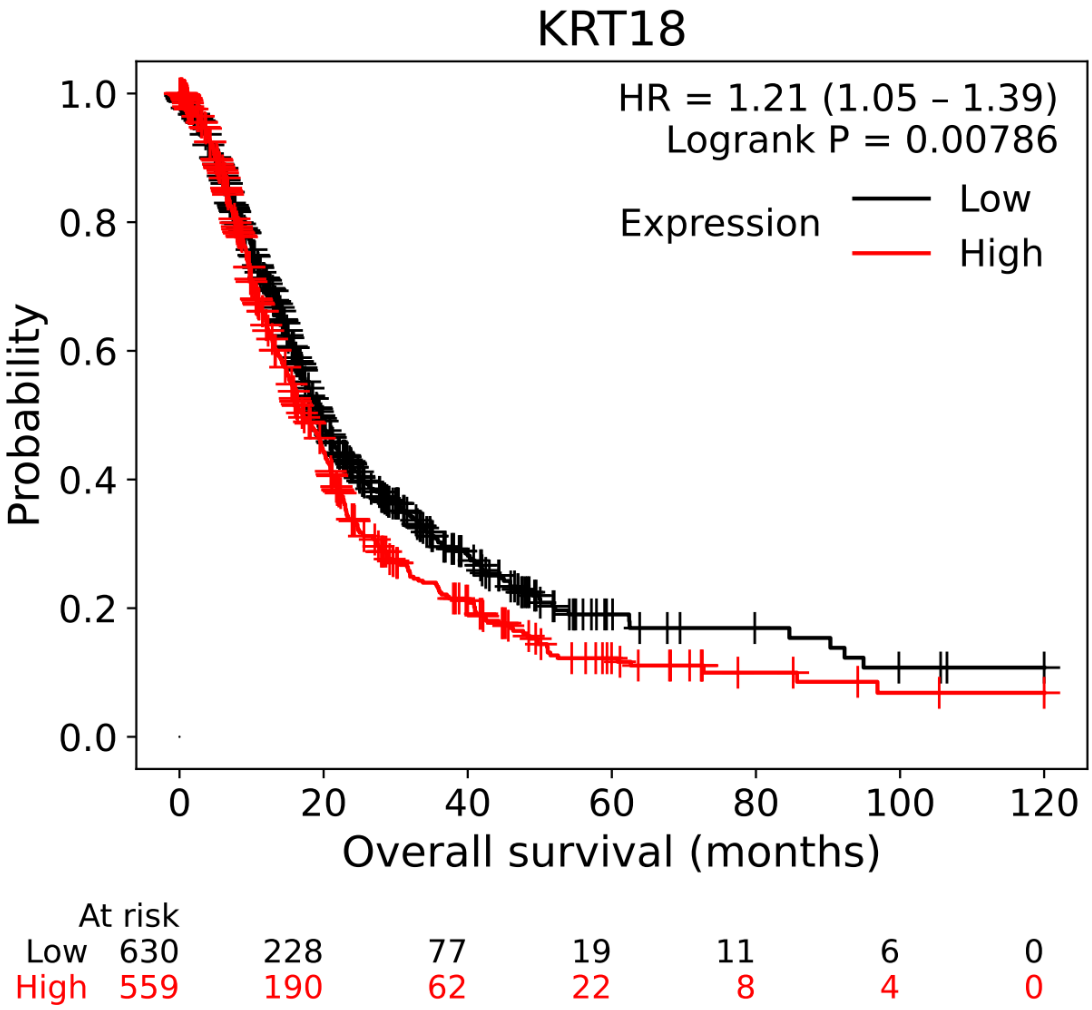

Figure S 7: Survival analysis for KRT18, which is an MI gene in the comparison between control and TGFB1-stimulated cells. HR > 1: High expression associated with worse overall survival (OS), HR < 1: High expression associated with better OS, Log-rank P < 0.05 considered statistically significant.

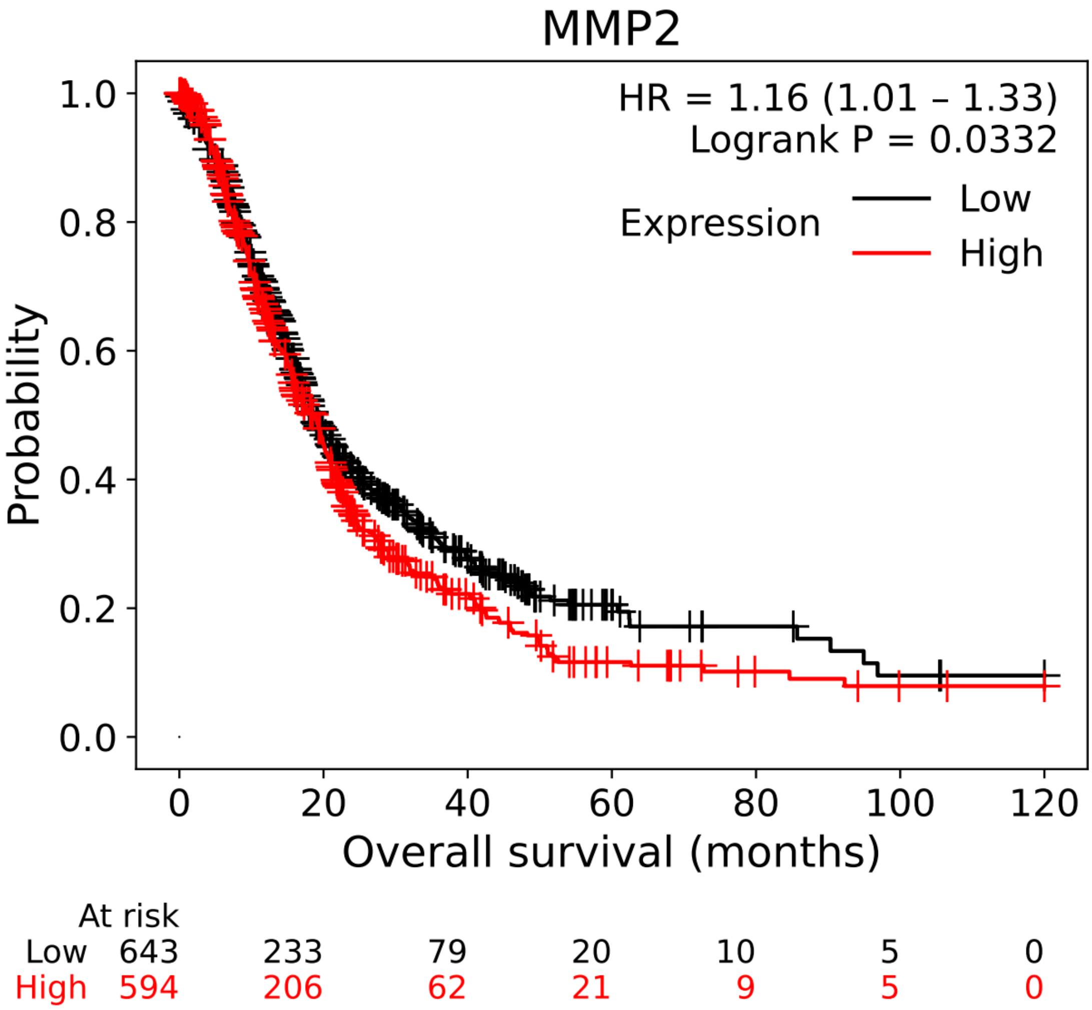

Figure S 8: Survival analysis for MMP2, which is an MI gene in the comparison between control and TGFB1-stimulated cells. HR > 1: High expression associated with worse overall survival (OS), HR < 1: High expression associated with better OS, Log-rank P < 0.05 considered statistically significant.

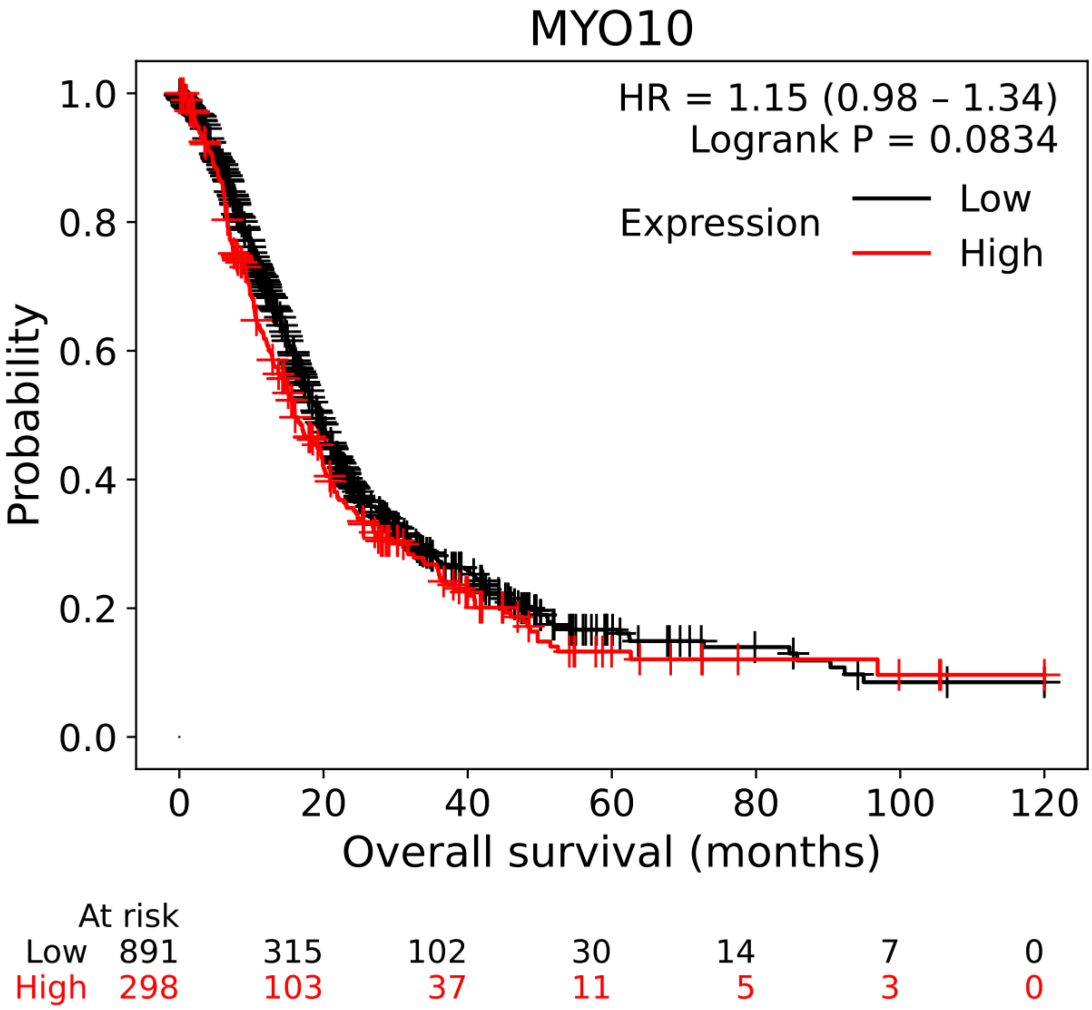

Figure S 9: Survival analysis for MYO10, which is an MI gene in the comparison between control and TGFB1-stimulated cells. HR > 1: High expression associated with worse overall survival (OS), HR < 1: High expression associated with better OS, Log-rank P < 0.05 considered statistically significant.

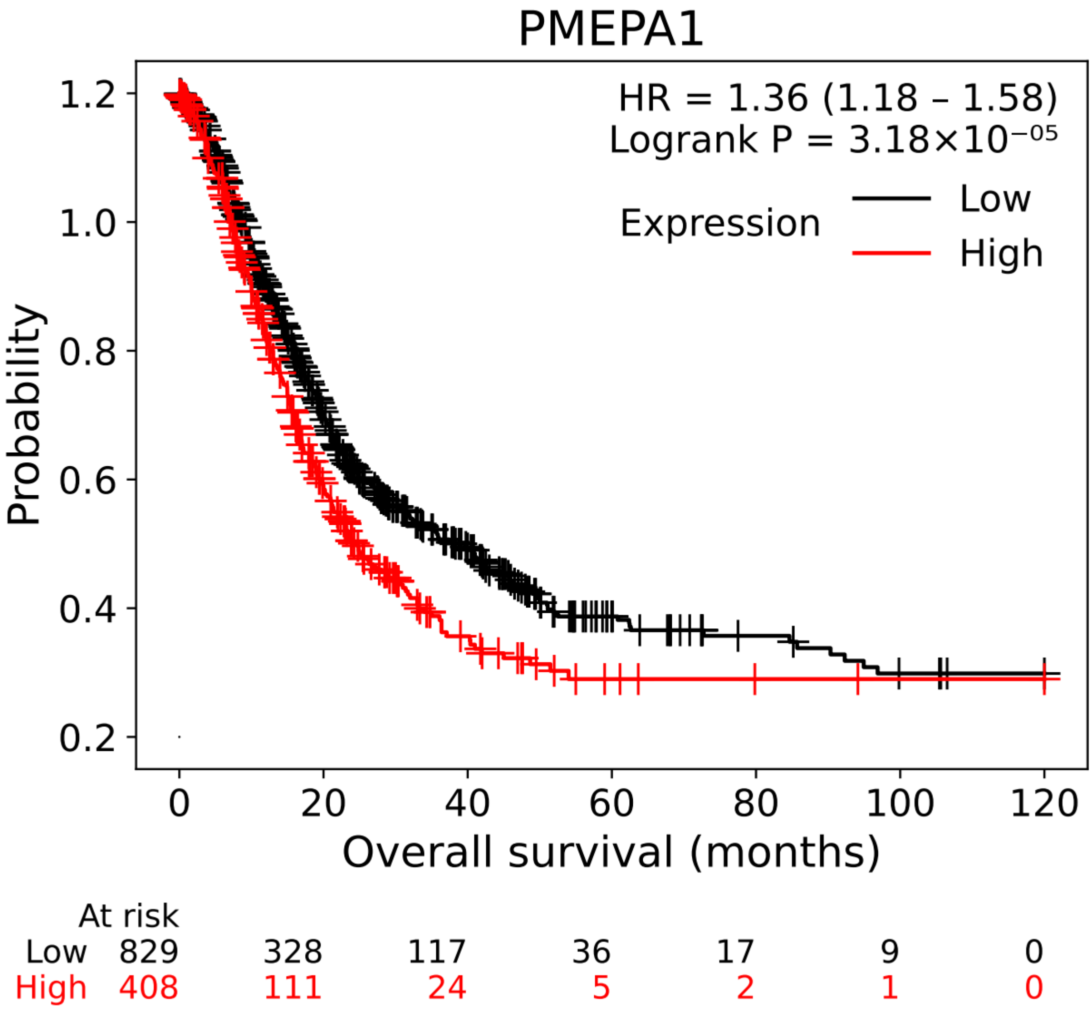

Figure S 10: Survival analysis for PMEPA1, which is an MI gene in the comparison between control and TGFB1-stimulated cells. HR > 1: High expression associated with worse overall survival (OS), HR < 1: High expression associated with better OS, Log-rank P < 0.05 considered statistically significant.

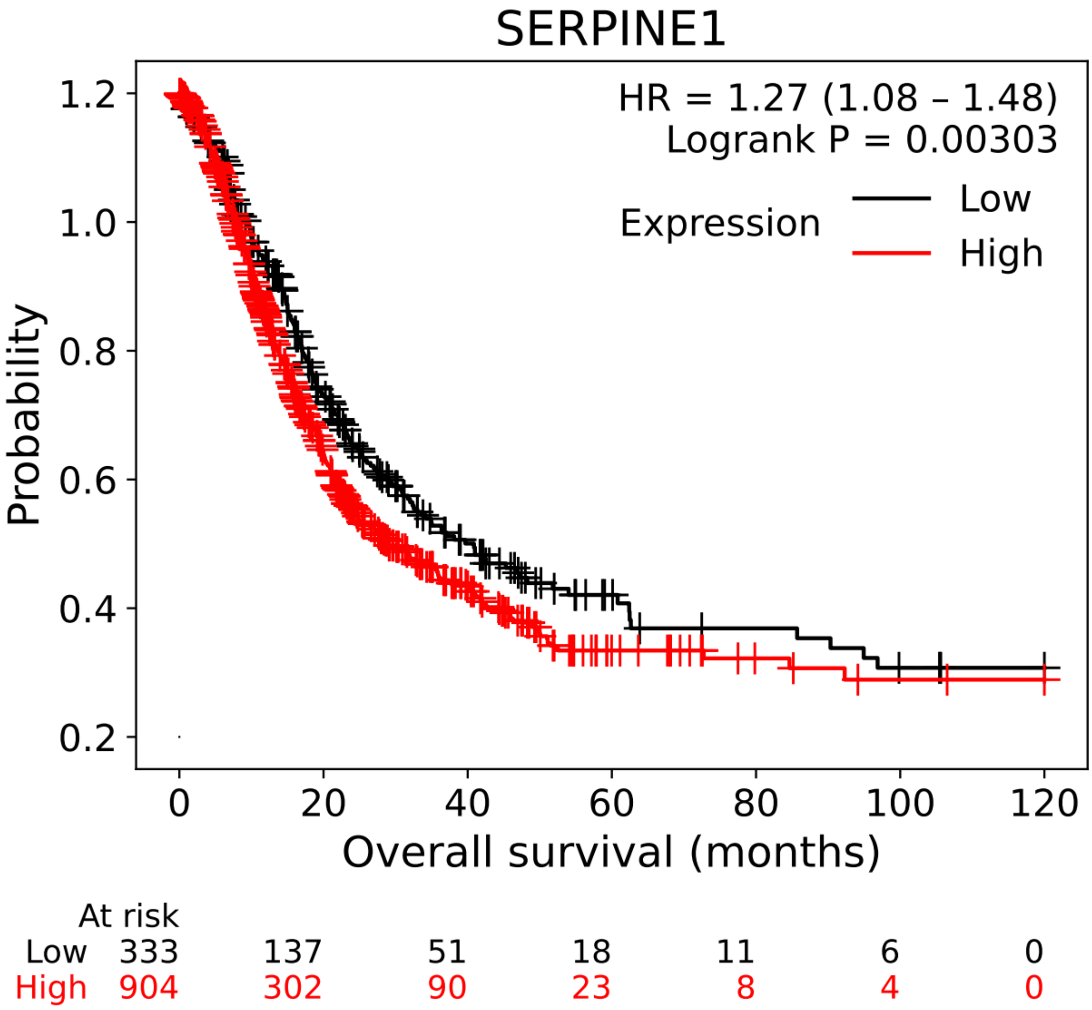

Figure S 11: Survival analysis for SERPINE1, which is an MI gene in the comparison between control and TGFB1-stimulated cells. HR > 1: High expression associated with worse overall survival (OS), HR < 1: High expression associated with better OS, Log-rank P < 0.05 considered statistically significant.

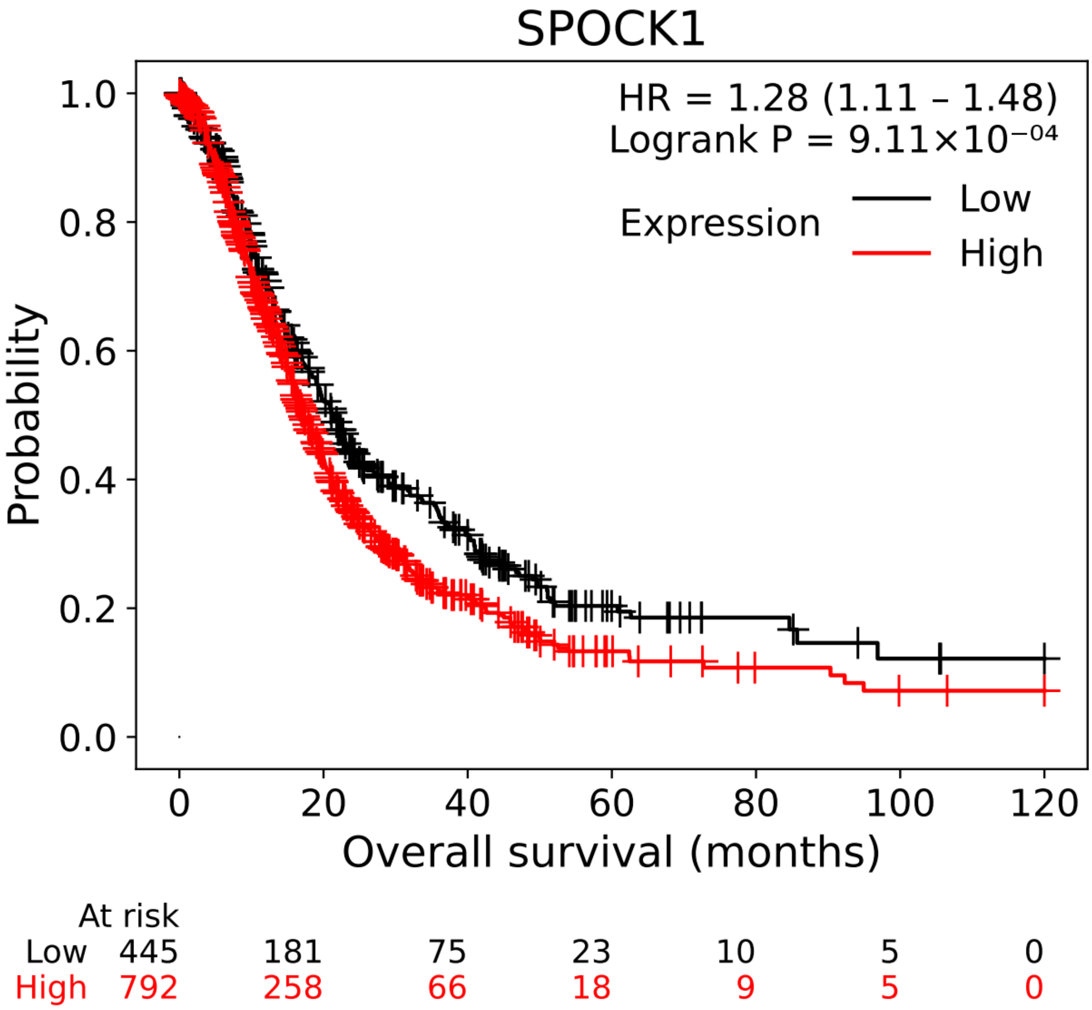

Figure S 12: Survival analysis for SPOCK1, which is an MI gene in the comparison between control and TGFB1-stimulated cells. HR > 1: High expression associated with worse overall survival (OS), HR < 1: High expression associated with better OS, Log-rank P < 0.05 considered statistically significant.

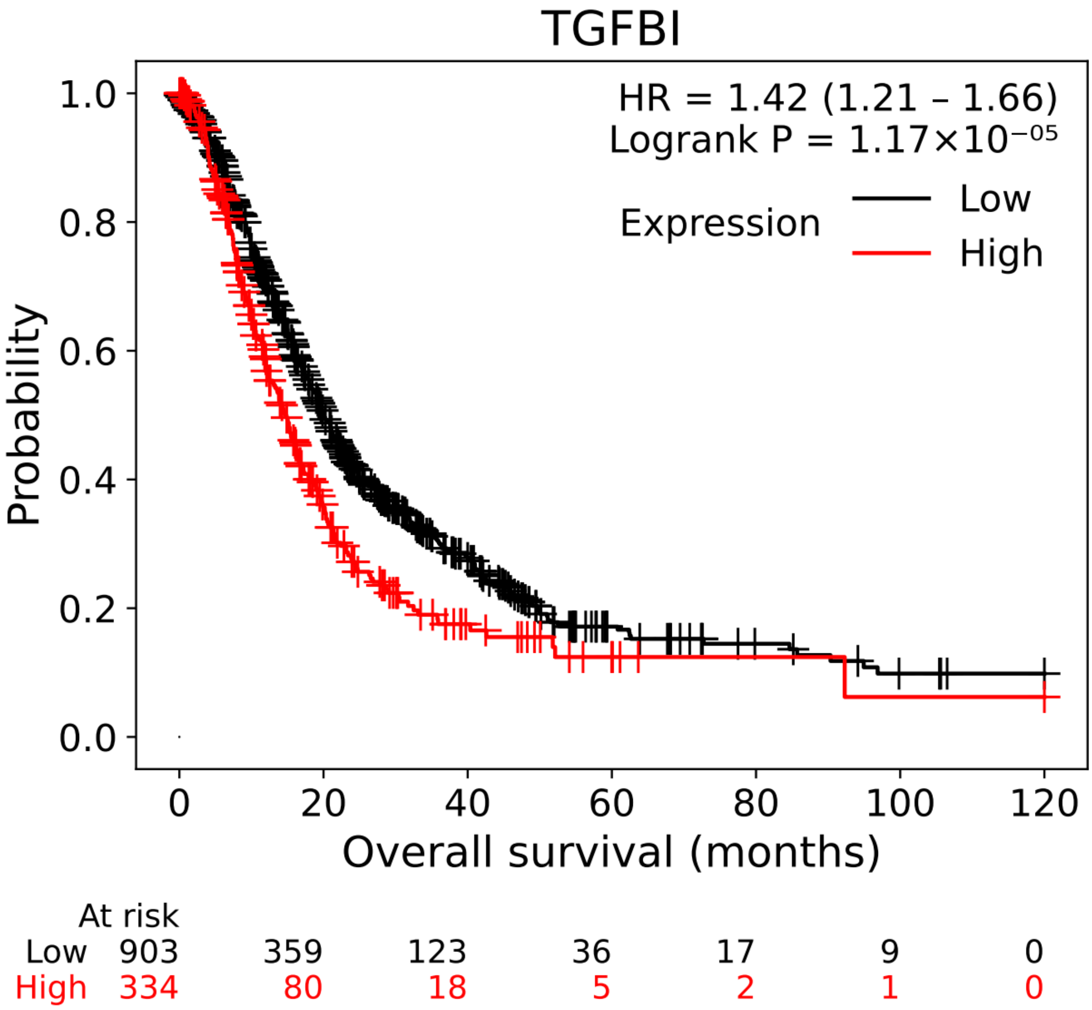

Figure S 13: Survival analysis for TGFBI, which is an MI gene in the comparison between control and TGFB1-stimulated cells. HR > 1: High expression associated with worse overall survival (OS), HR < 1: High expression associated with better OS, Log-rank P < 0.05 considered statistically significant.

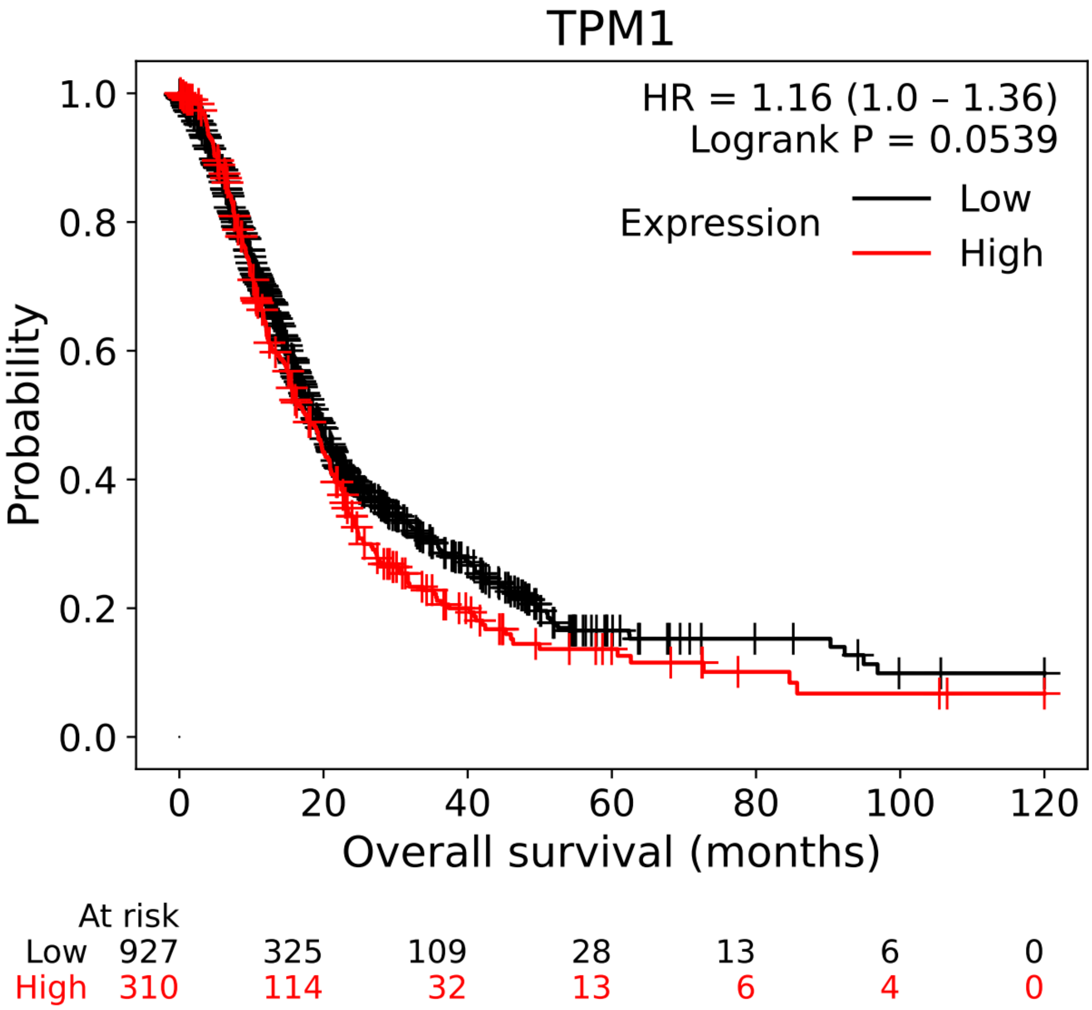

Figure S 14: Survival analysis for TPM1, which is an MI gene in the comparison between control and TGFB1-stimulated cells. HR > 1: High expression associated with worse overall survival (OS), HR < 1: High expression associated with better OS, Log-rank P < 0.05 considered statistically significant.

Survival analyses of the ten top-ranked mutual information (MI) genes for the comparison between control and TGFB1+GEM-treated cells

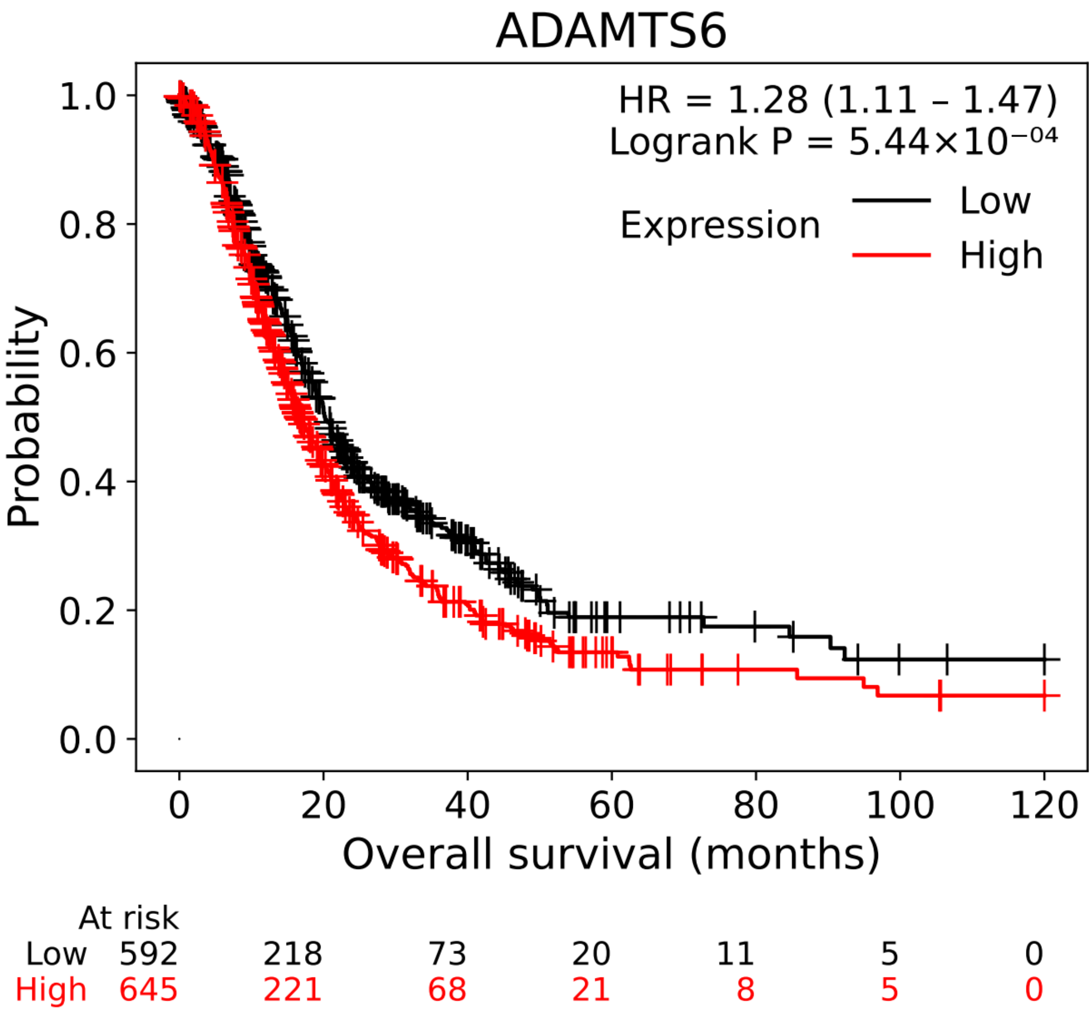

Figure S 15: Survival analysis for ADAMTS6, which is an MI gene in the comparison between control and TGFB1+GEM-treated cells. HR > 1: High expression associated with worse overall survival (OS), HR < 1: High expression associated with better OS, Log-rank P < 0.05 considered statistically significant.

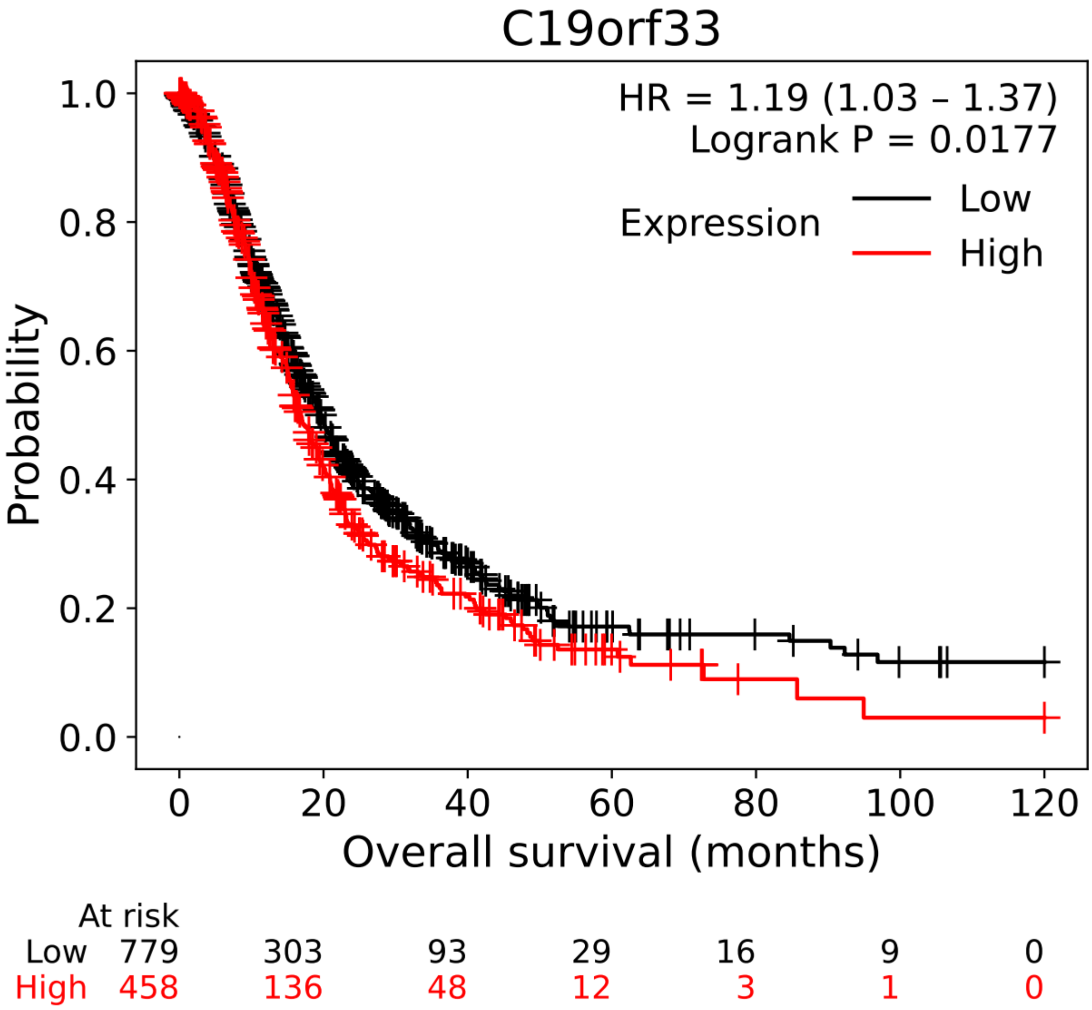

Figure S 16: Survival analysis for C19orf33, which is an MI gene in the comparison between control and TGFB1+GEM-treated cells. HR > 1: High expression associated with worse overall survival (OS), HR < 1: High expression associated with better OS, Log-rank P < 0.05 considered statistically significant.

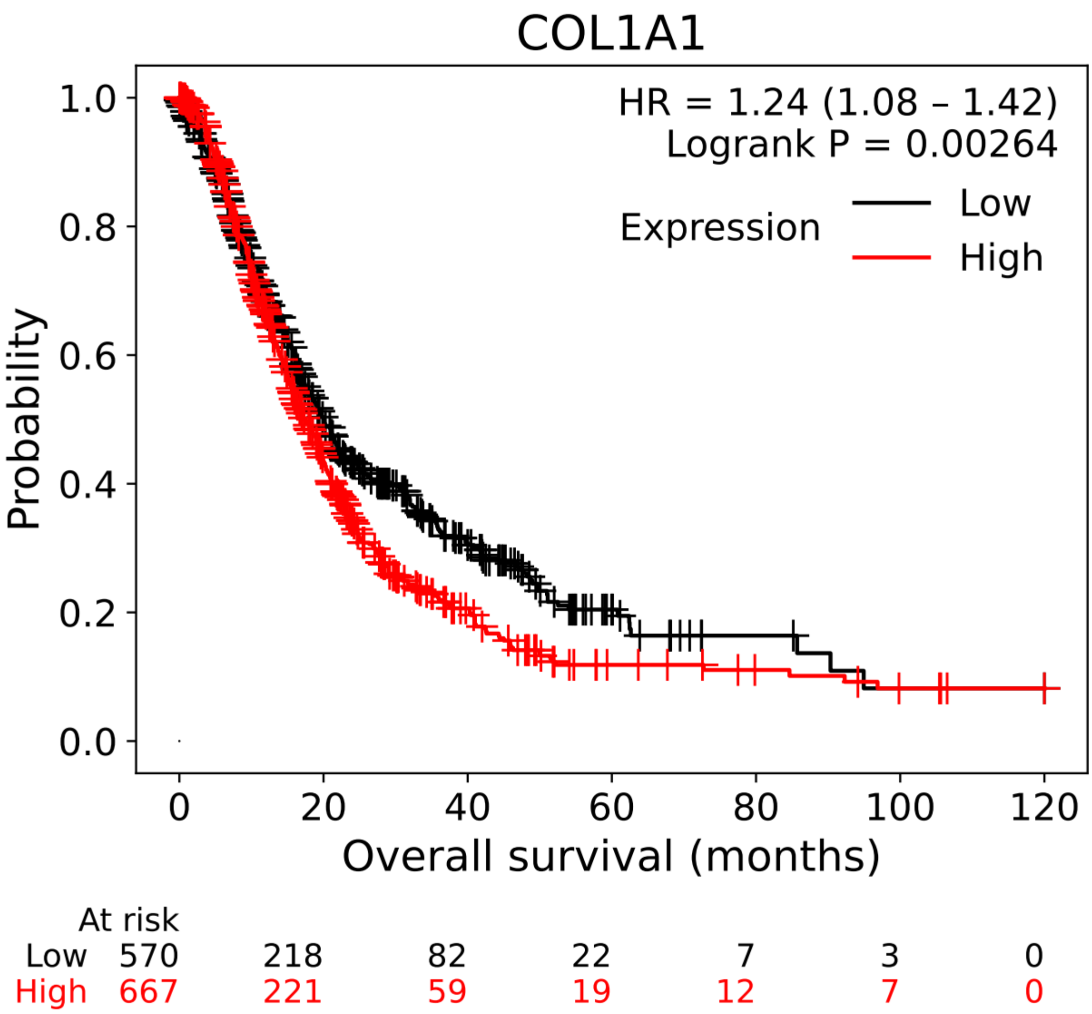

Figure S 17: Survival analysis for COL1A1, which is an MI gene in the comparison between control and TGFB1+GEM-treated cells. HR > 1: High expression associated with worse overall survival (OS), HR < 1: High expression associated with better OS, Log-rank P < 0.05 considered statistically significant.

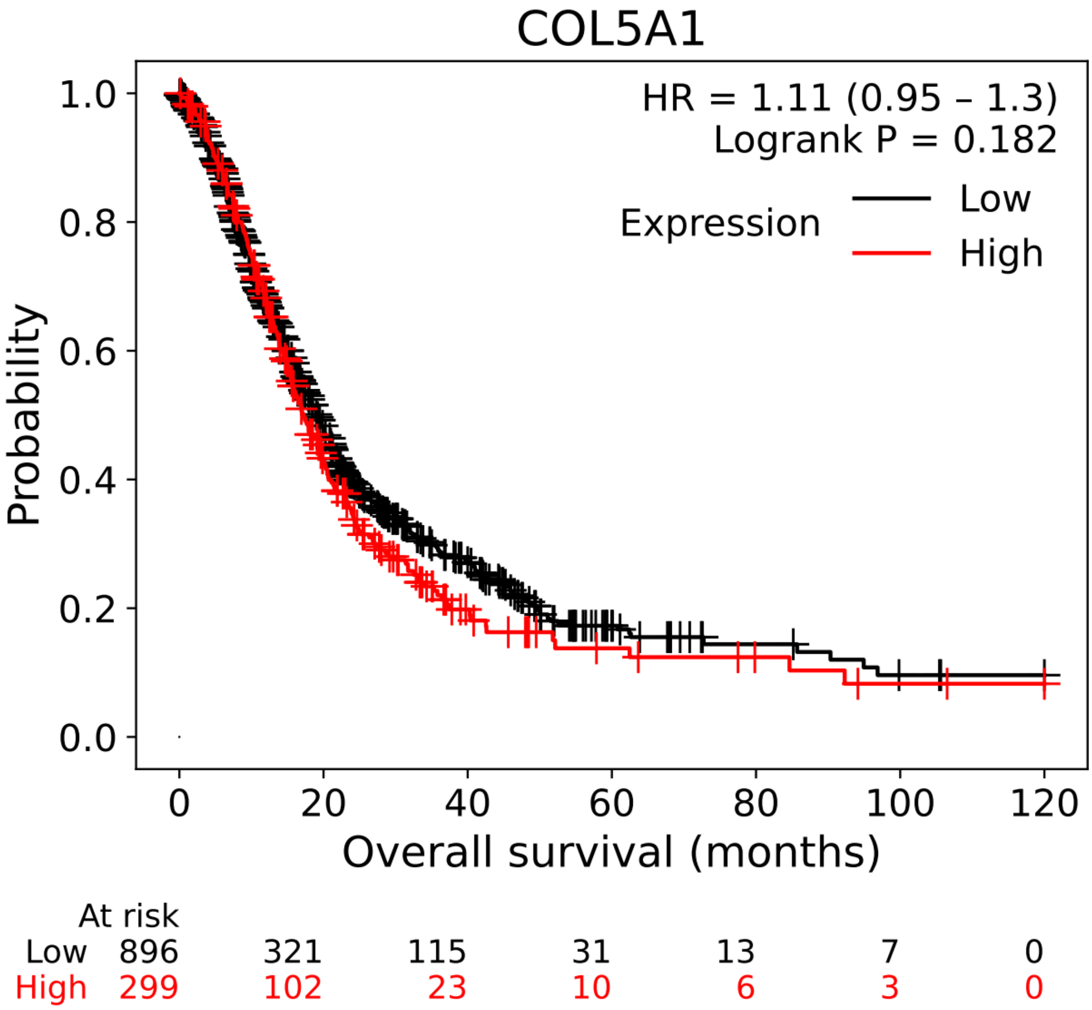

Figure S 18: Survival analysis for COL5A1, which is an MI gene in the comparison between control and TGFB1+GEM-treated cells. HR > 1: High expression associated with worse overall survival (OS), HR < 1: High expression associated with better OS, Log-rank P < 0.05 considered statistically significant.

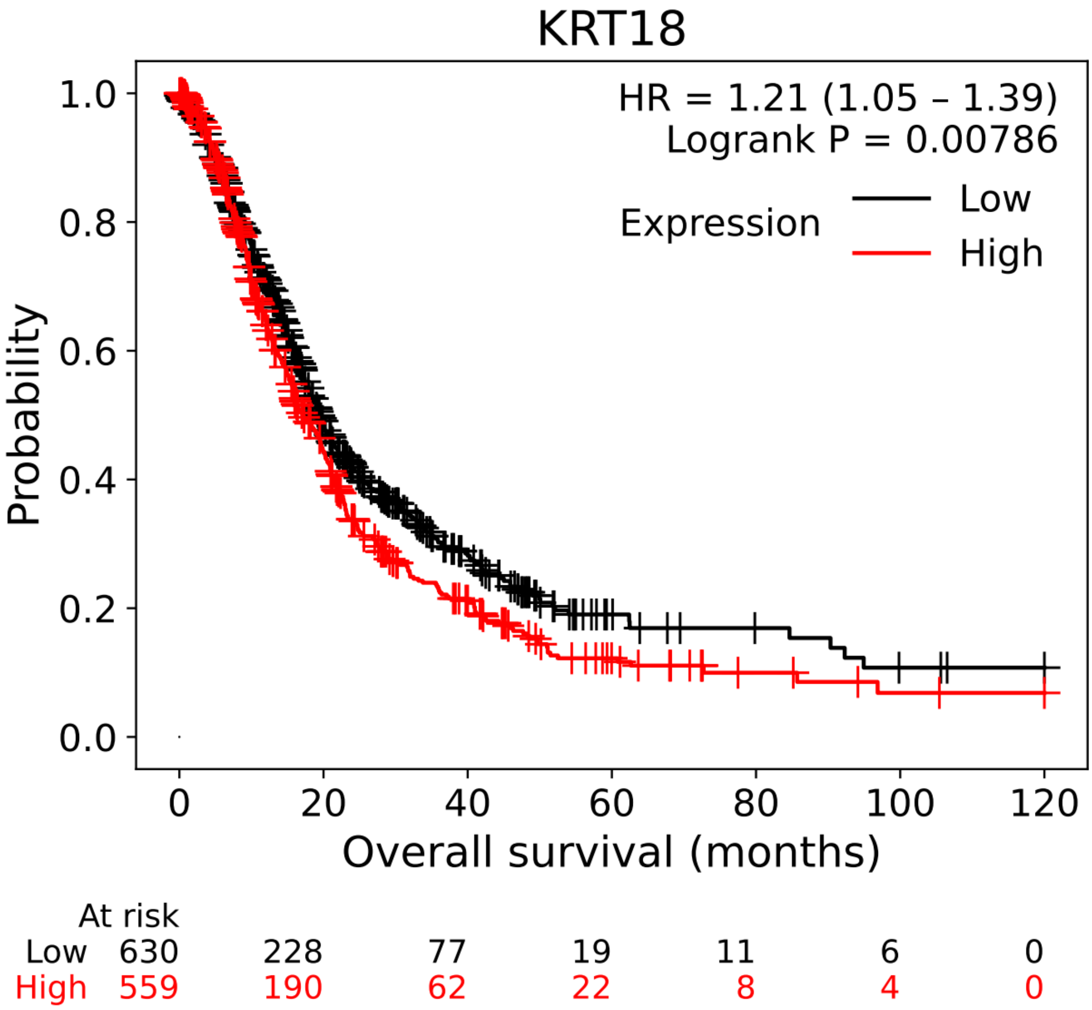

Figure S 19: Survival analysis for KRT18, which is an MI gene in the comparison between control and TGFB1+GEM-treated cells. HR > 1: High expression associated with worse overall survival (OS), HR < 1: High expression associated with better OS, Log-rank P < 0.05 considered statistically significant.

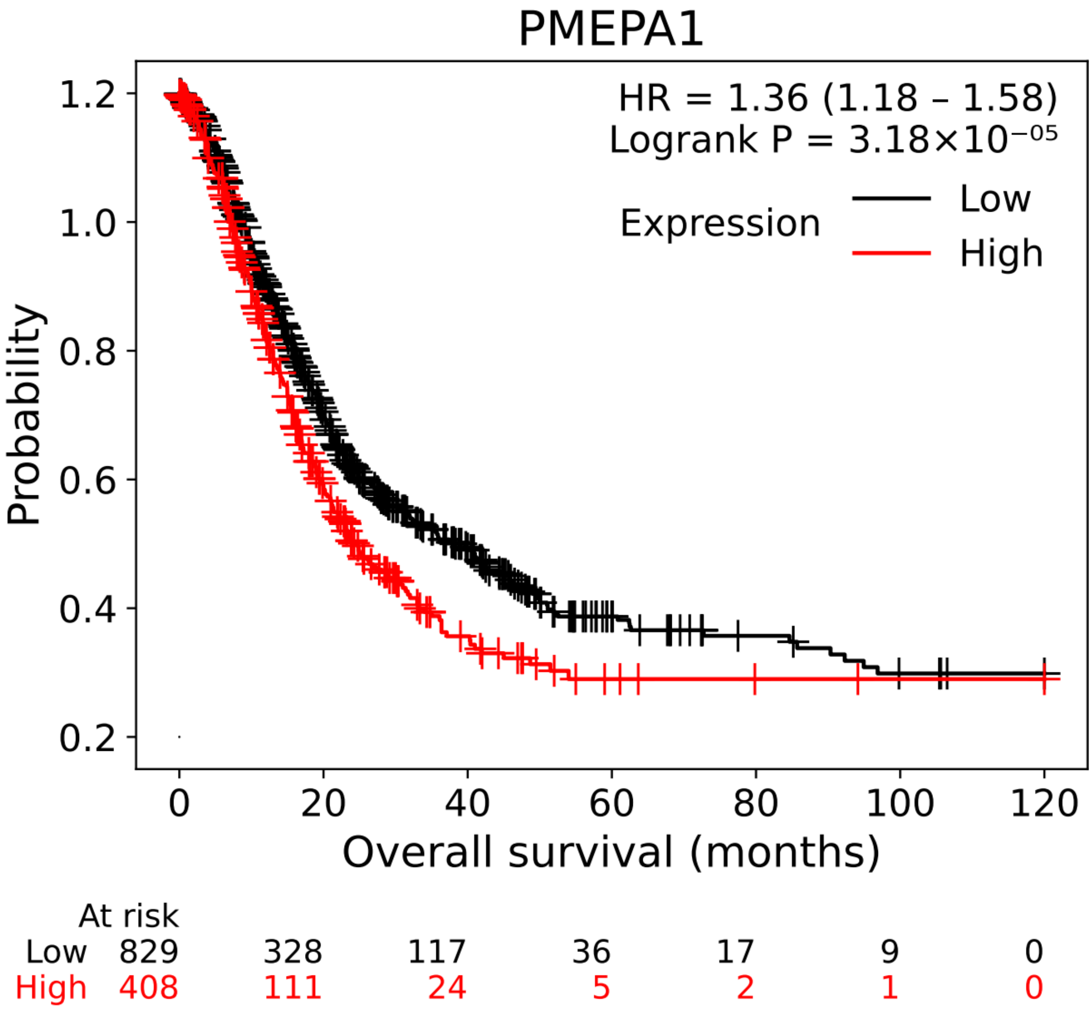

Figure S 20: Survival analysis for PMEPA1, which is an MI gene in the comparison between control and TGFB1+GEM-treated cells. HR > 1: High expression associated with worse overall survival (OS), HR < 1: High expression associated with better OS, Log-rank P < 0.05 considered statistically significant.

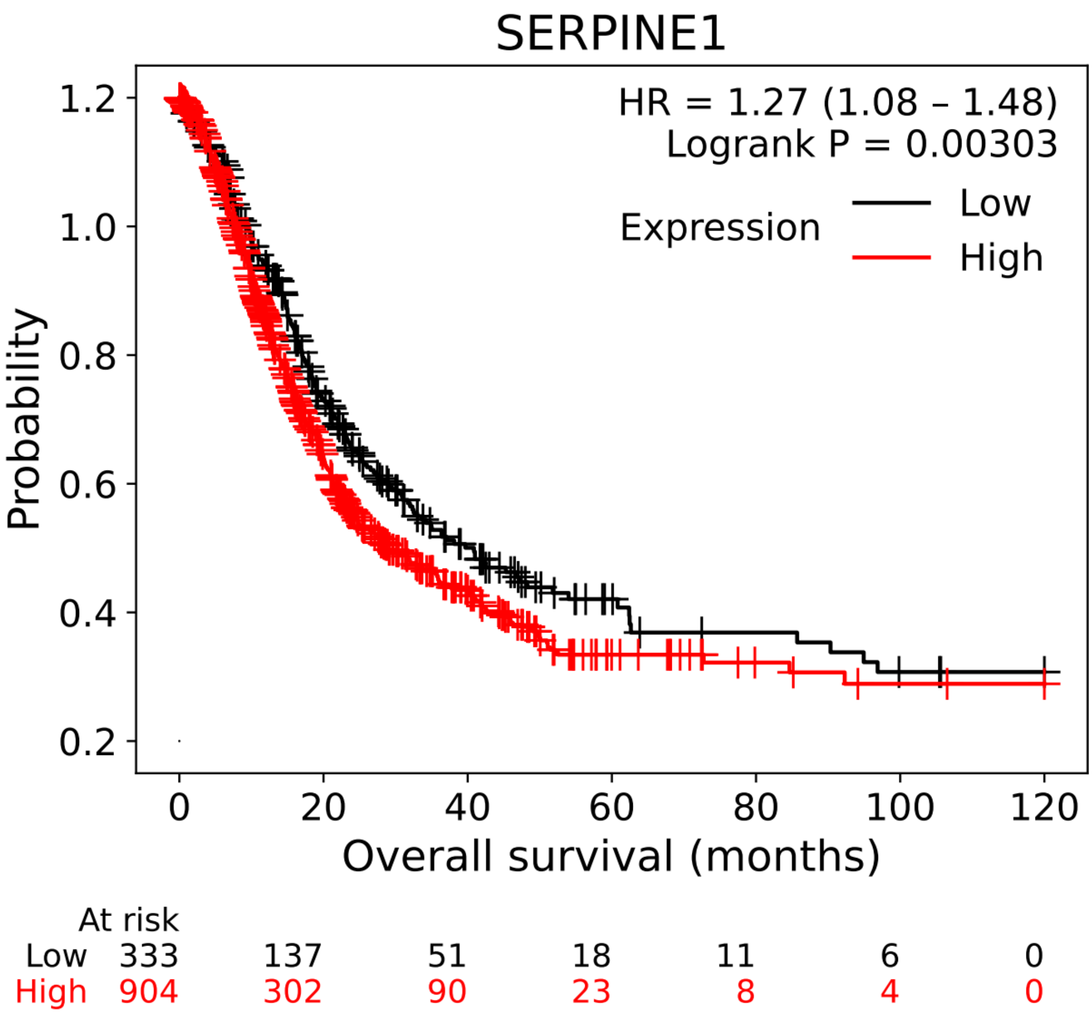

Figure S 21: Survival analysis for SERPINE1, which is an MI gene in the comparison between control and TGFB1+GEM-treated cells. HR > 1: High expression associated with worse overall survival (OS), HR < 1: High expression associated with better OS, Log-rank P < 0.05 considered statistically significant.

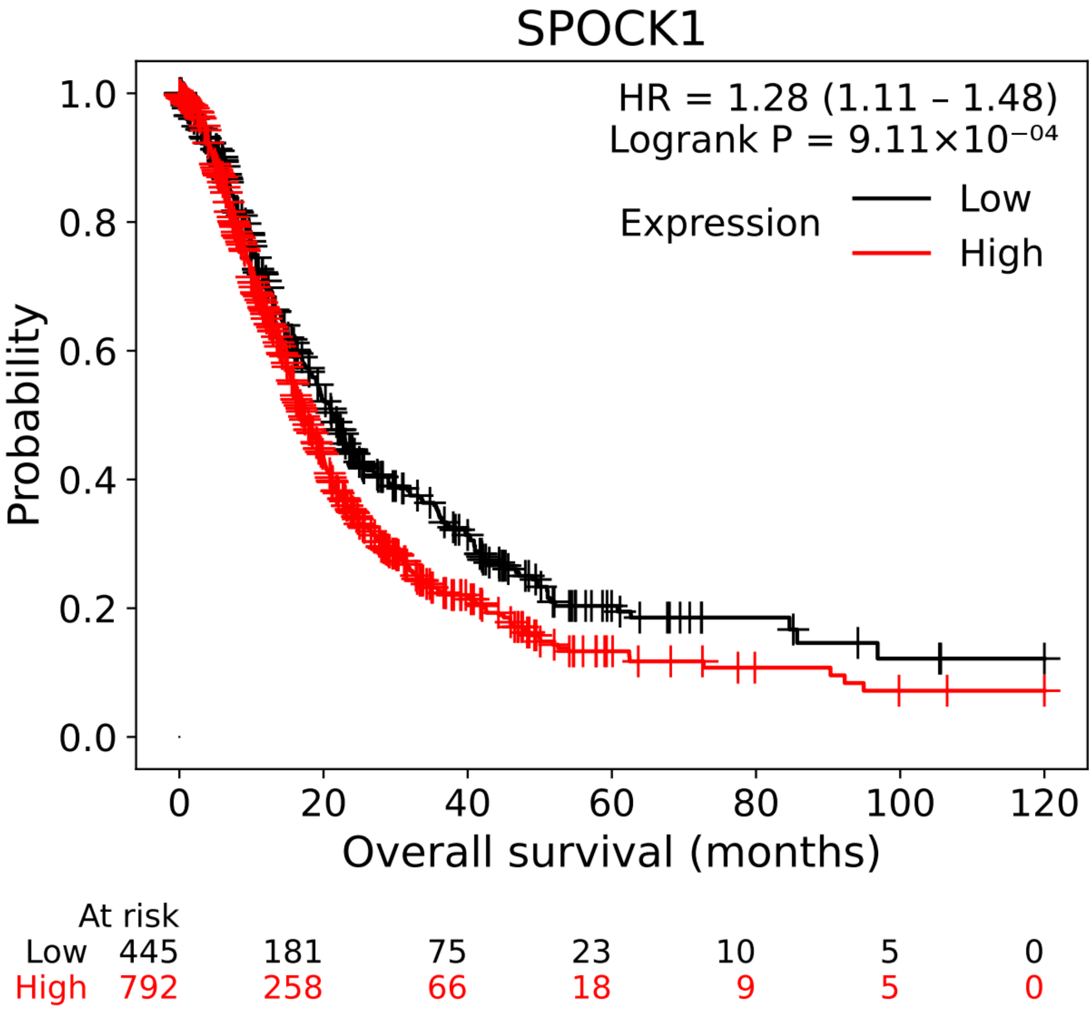

Figure S 22: Survival analysis for SPOCK1, which is an MI gene in the comparison between control and TGFB1+GEM-treated cells. HR > 1: High expression associated with worse overall survival (OS), HR < 1: High expression associated with better OS, Log-rank P < 0.05 considered statistically significant.

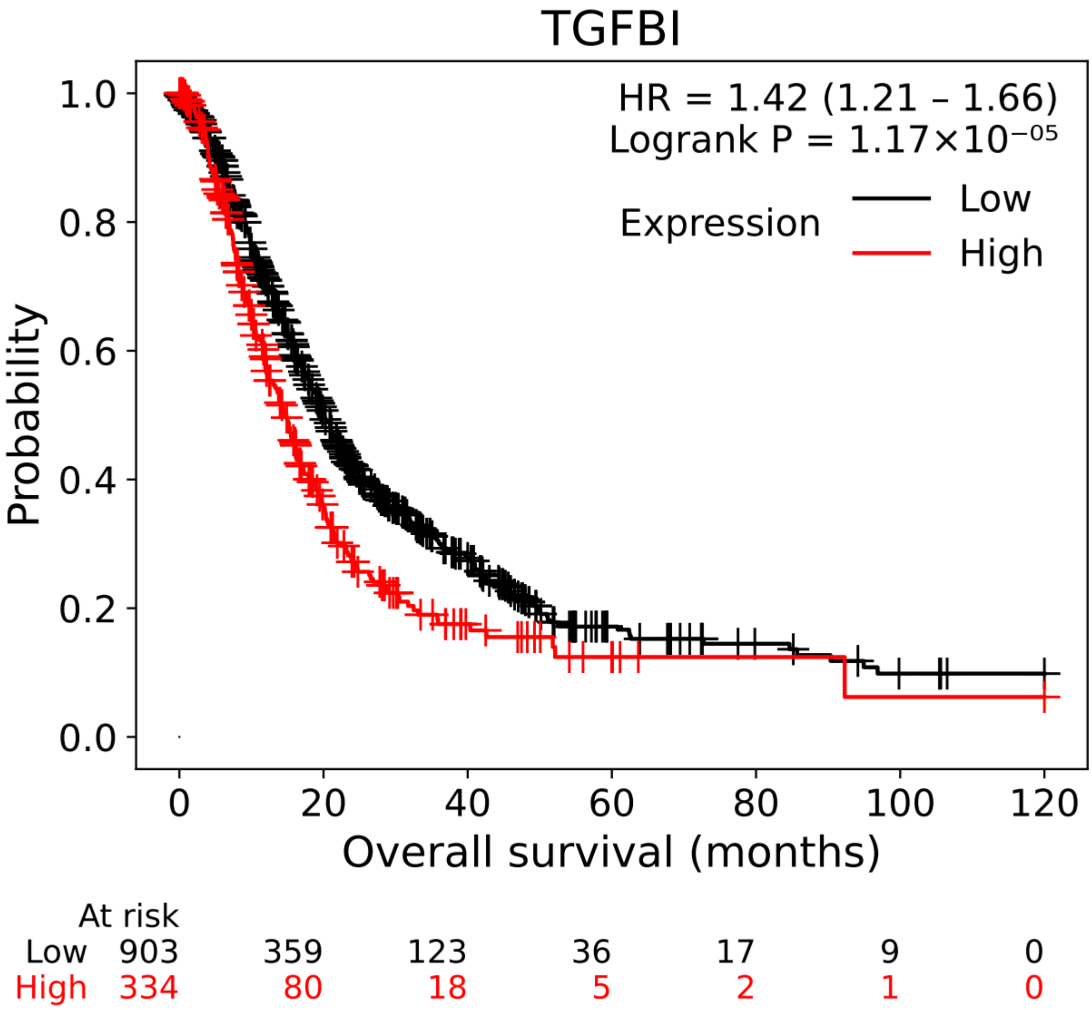

Figure S 23: Survival analysis for TGFBI, which is an MI gene in the comparison between control and TGFB1+GEM-treated cells. HR > 1: High expression associated with worse overall survival (OS), HR < 1: High expression associated with better OS, Log-rank P < 0.05 considered statistically significant.

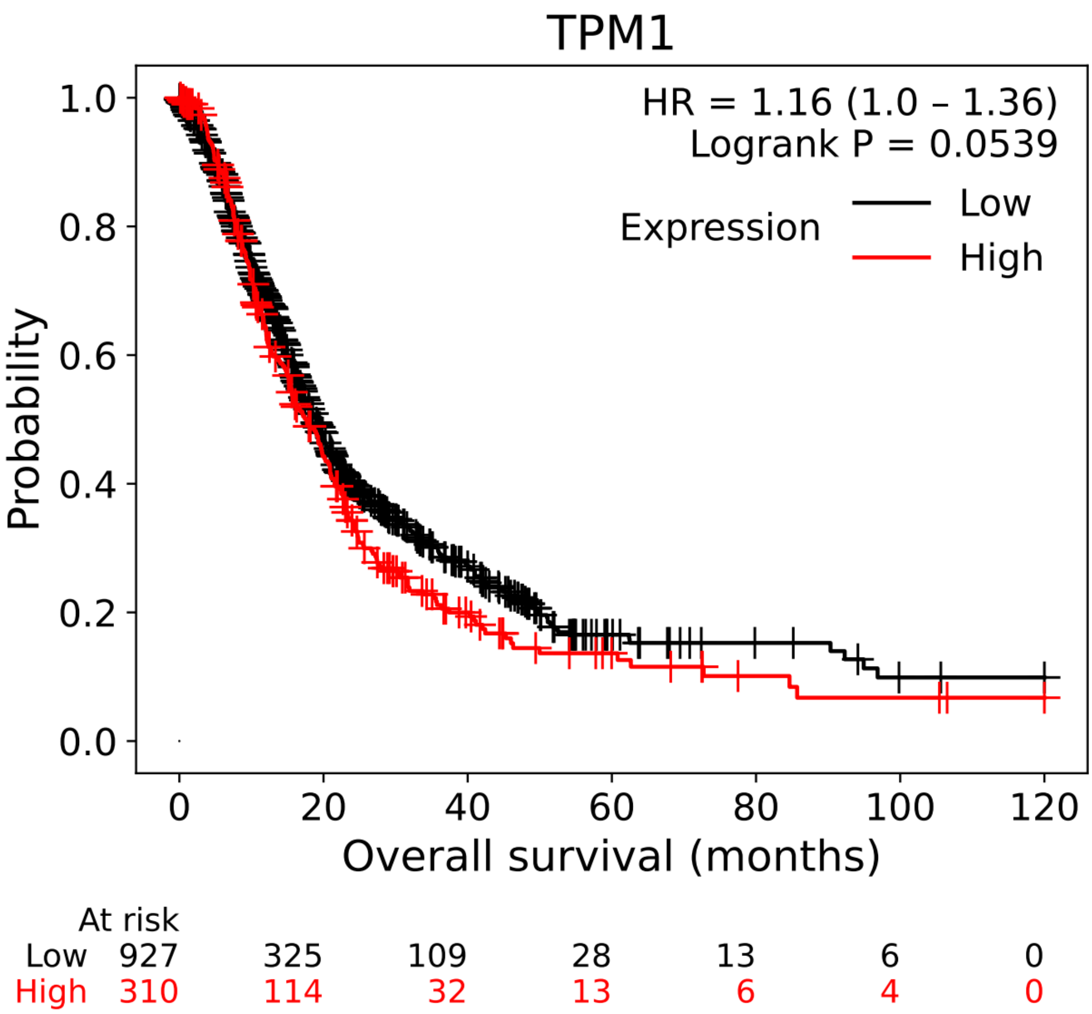

Figure S 24: Survival analysis for TPM1, which is an MI gene in the comparison between control and TGFB1+GEM-treated cells. HR > 1: High expression associated with worse overall survival (OS), HR < 1: High expression associated with better OS, Log-rank P < 0.05 considered statistically significant.

Survival analyses of the ten top-ranked mutual information (MI) genes for the comparison between TGFB1 stimulated cells and TGFB1+GEM-treated cells

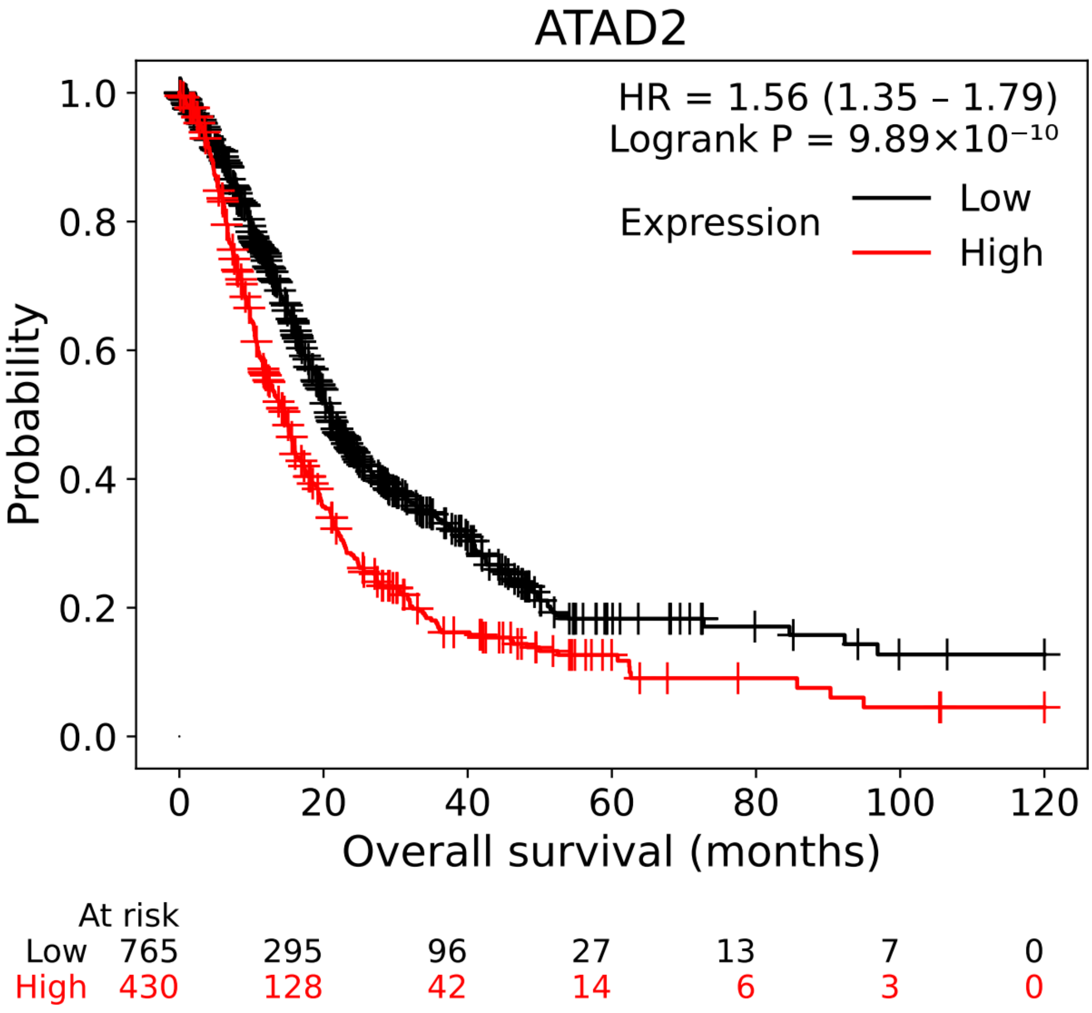

Figure S 25: Survival analysis for ATAD2, which is an MI gene in the comparison between TGFB1 stimulated cells and TGFB1+GEM-treated cells. HR > 1: High expression associated with worse overall survival (OS), HR < 1: High expression associated with better OS, Log-rank P < 0.05 considered statistically significant.

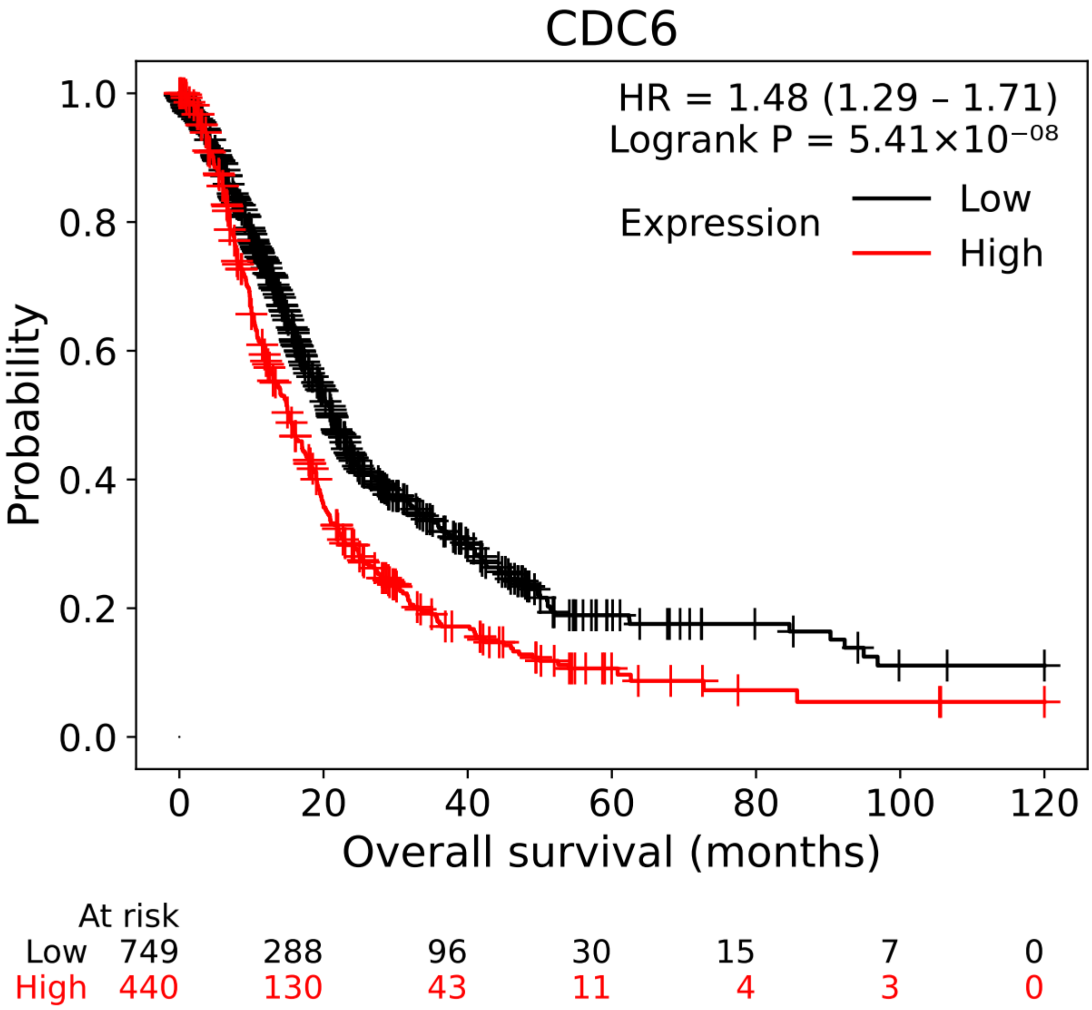

Figure S 26: Survival analysis for CDC6, which is an MI gene in the comparison between TGFB1 stimulated cells and TGFB1+GEM-treated cells. HR > 1: High expression associated with worse overall survival (OS), HR < 1: High expression associated with better OS, Log-rank P < 0.05 considered statistically significant.

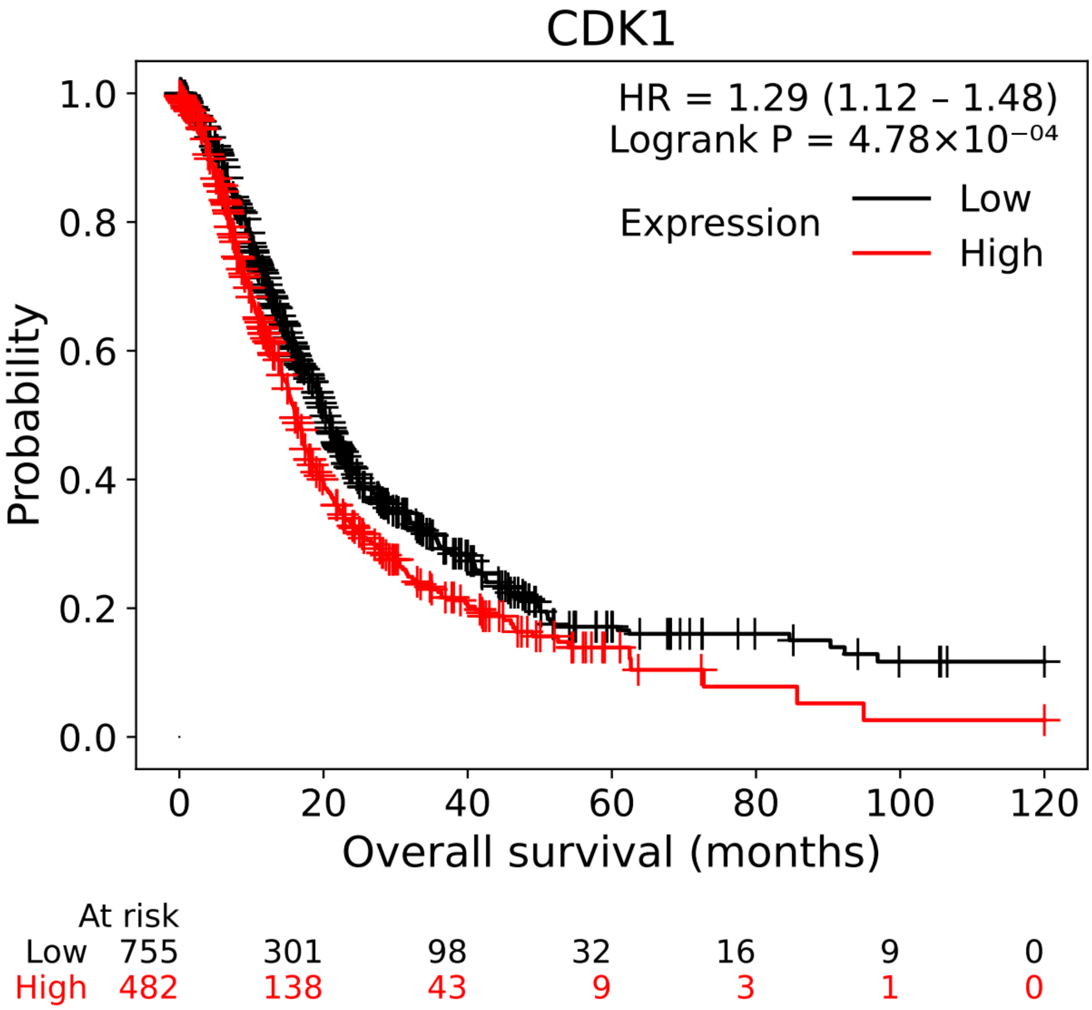

Figure S 27: Survival analysis for CDK1, which is an MI gene in the comparison between TGFB1 stimulated cells and TGFB1+GEM-treated cells. HR > 1: High expression associated with worse overall survival (OS), HR < 1: High expression associated with better OS, Log-rank P < 0.05 considered statistically significant.

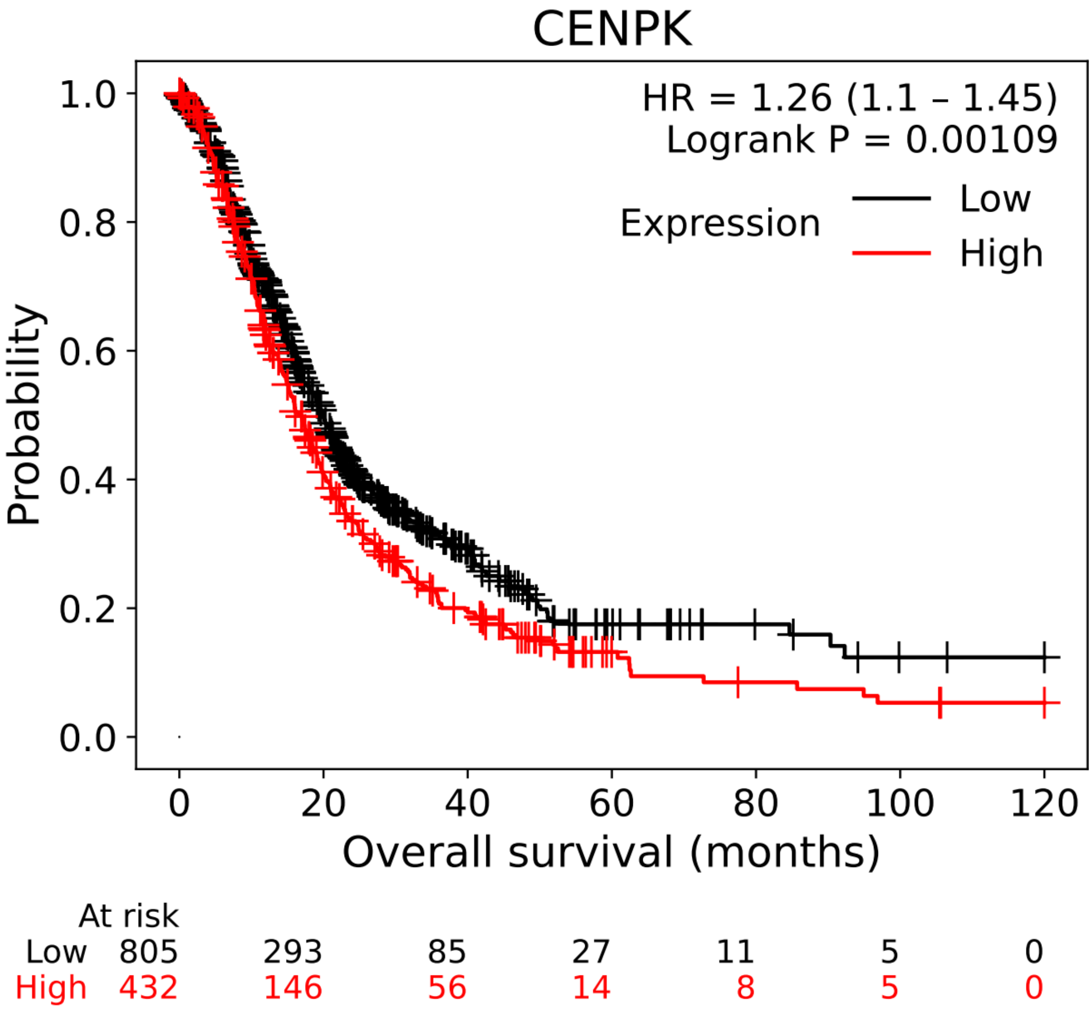

Figure S 28: Survival analysis for CENPK, which is an MI gene in the comparison between TGFB1 stimulated cells and TGFB1+GEM-treated cells. HR > 1: High expression associated with worse overall survival (OS), HR < 1: High expression associated with better OS, Log-rank P < 0.05 considered statistically significant.

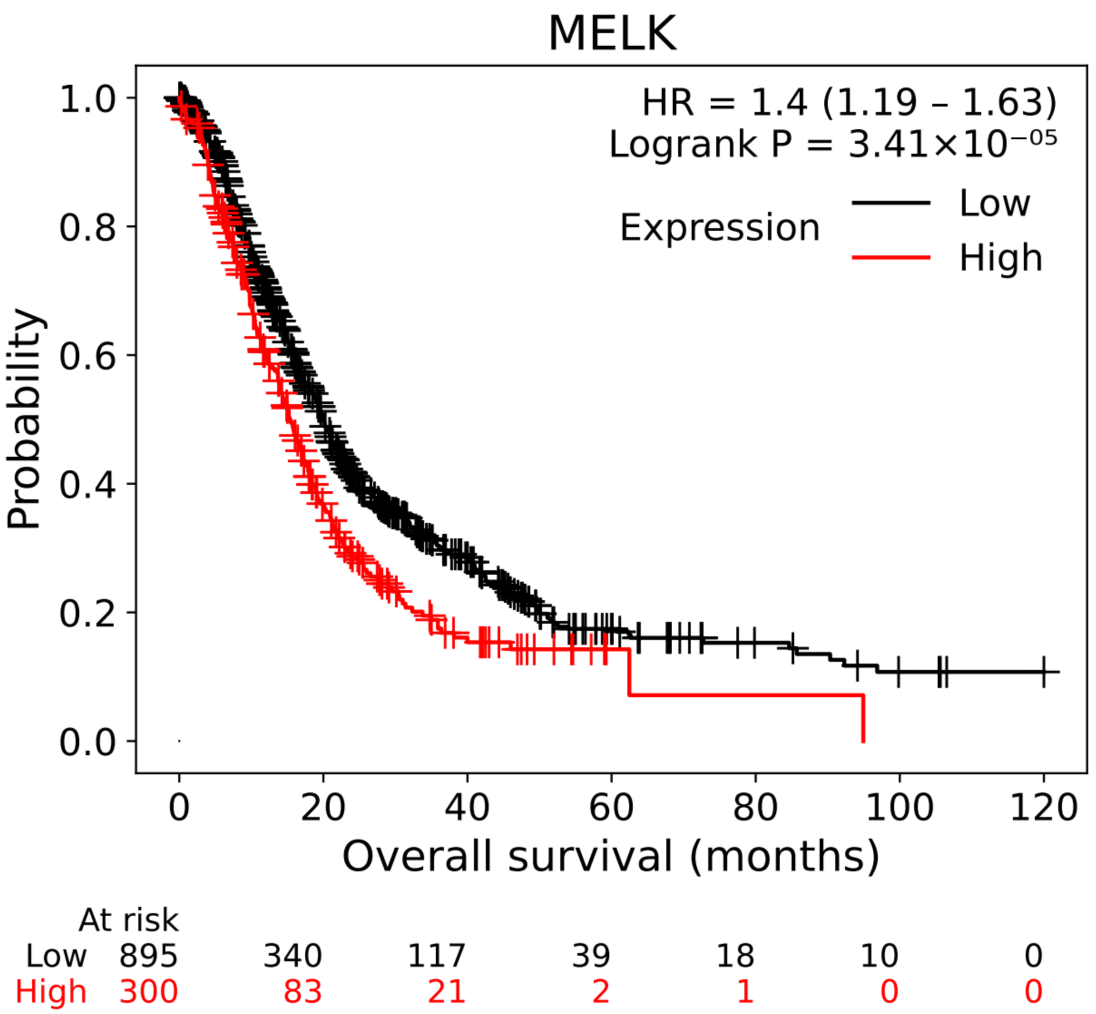

Figure S 29: Survival analysis for MELK, which is an MI gene in the comparison between TGFB1 stimulated cells and TGFB1+GEM-treated cells. HR > 1: High expression associated with worse overall survival (OS), HR < 1: High expression associated with better OS, Log-rank P < 0.05 considered statistically significant.

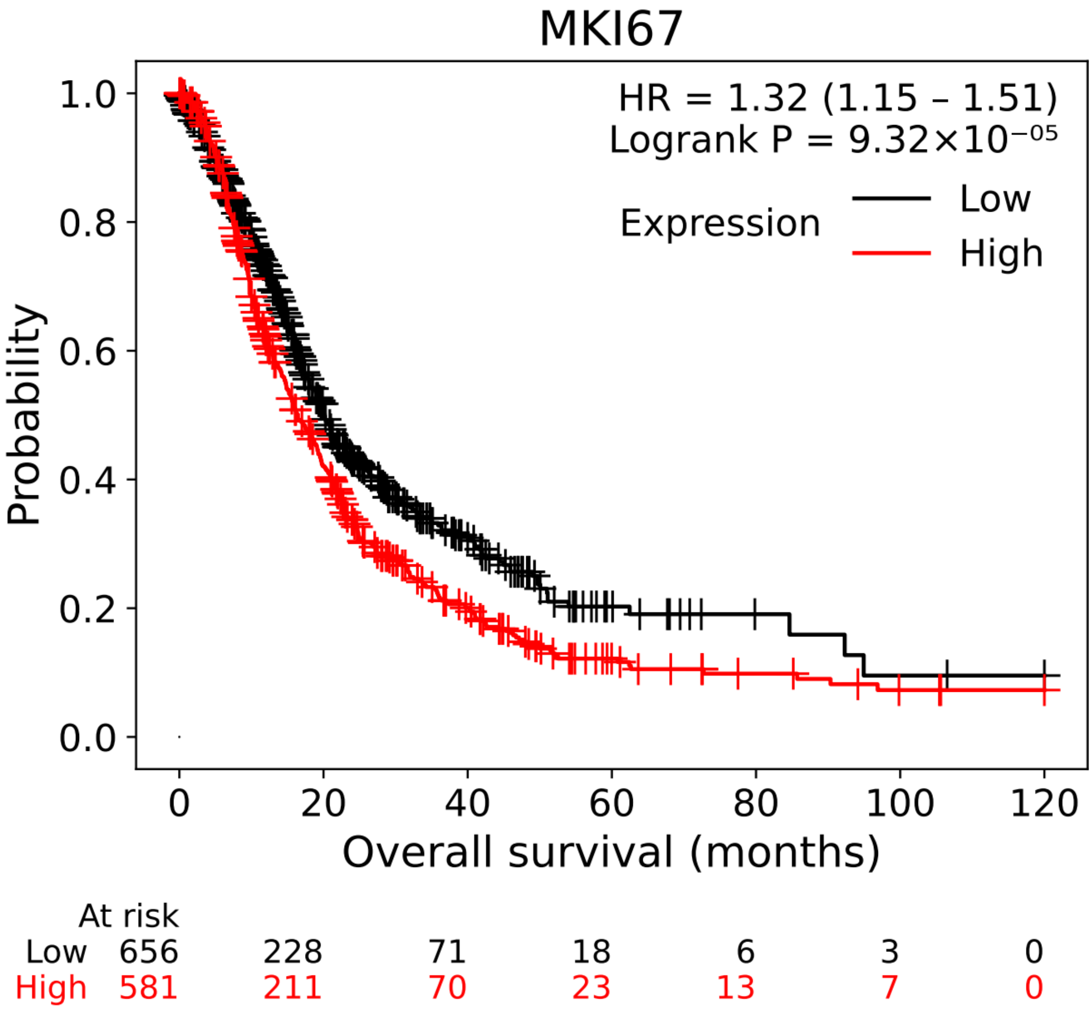

Figure S 30: Survival analysis for MKI67, which is an MI gene in the comparison between TGFB1 stimulated cells and TGFB1+GEM-treated cells. HR > 1: High expression associated with worse overall survival (OS), HR < 1: High expression associated with better OS, Log-rank P < 0.05 considered statistically significant.

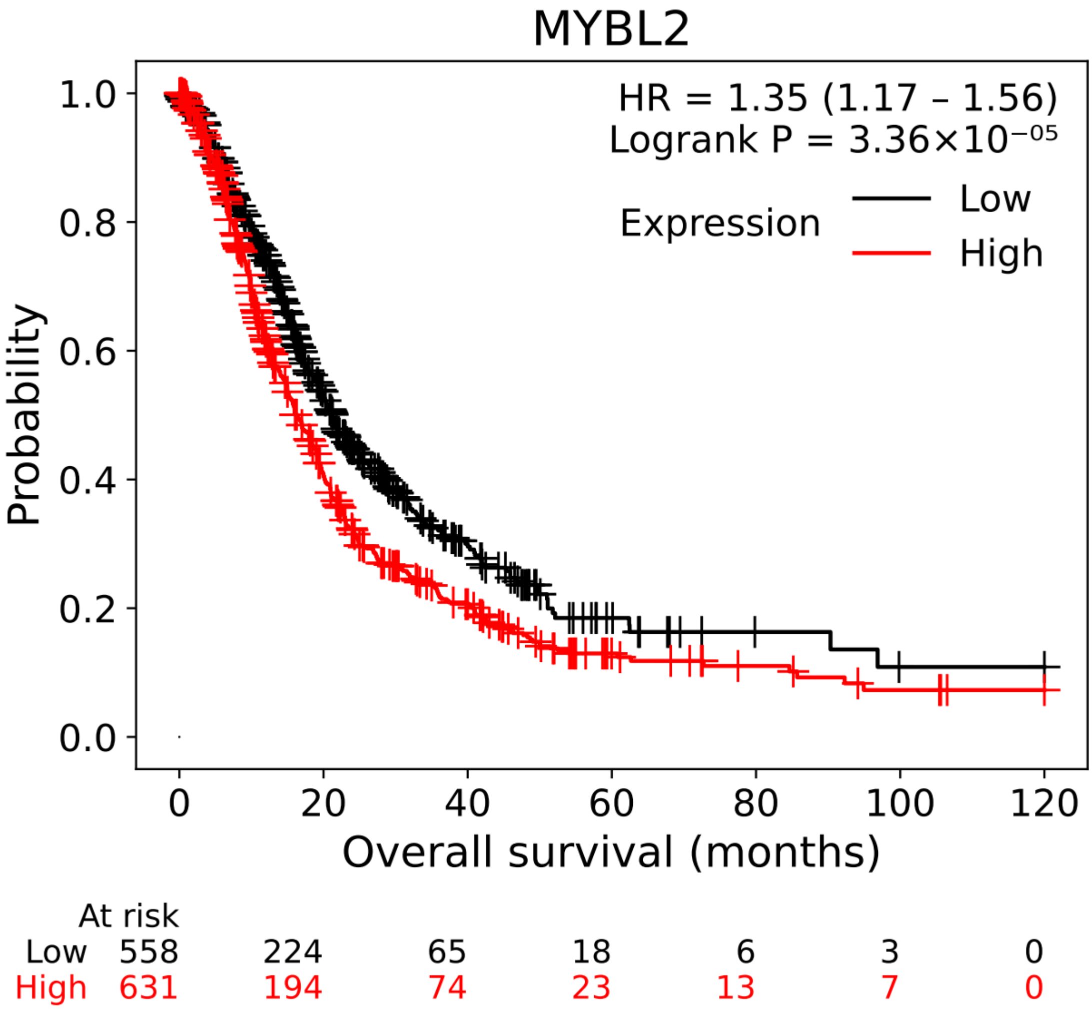

Figure S 31: Survival analysis for MYBL2, which is an MI gene in the comparison between TGFB1 stimulated cells and TGFB1+GEM-treated cells. HR > 1: High expression associated with worse overall survival (OS), HR < 1: High expression associated with better OS, Log-rank P < 0.05 considered statistically significant.

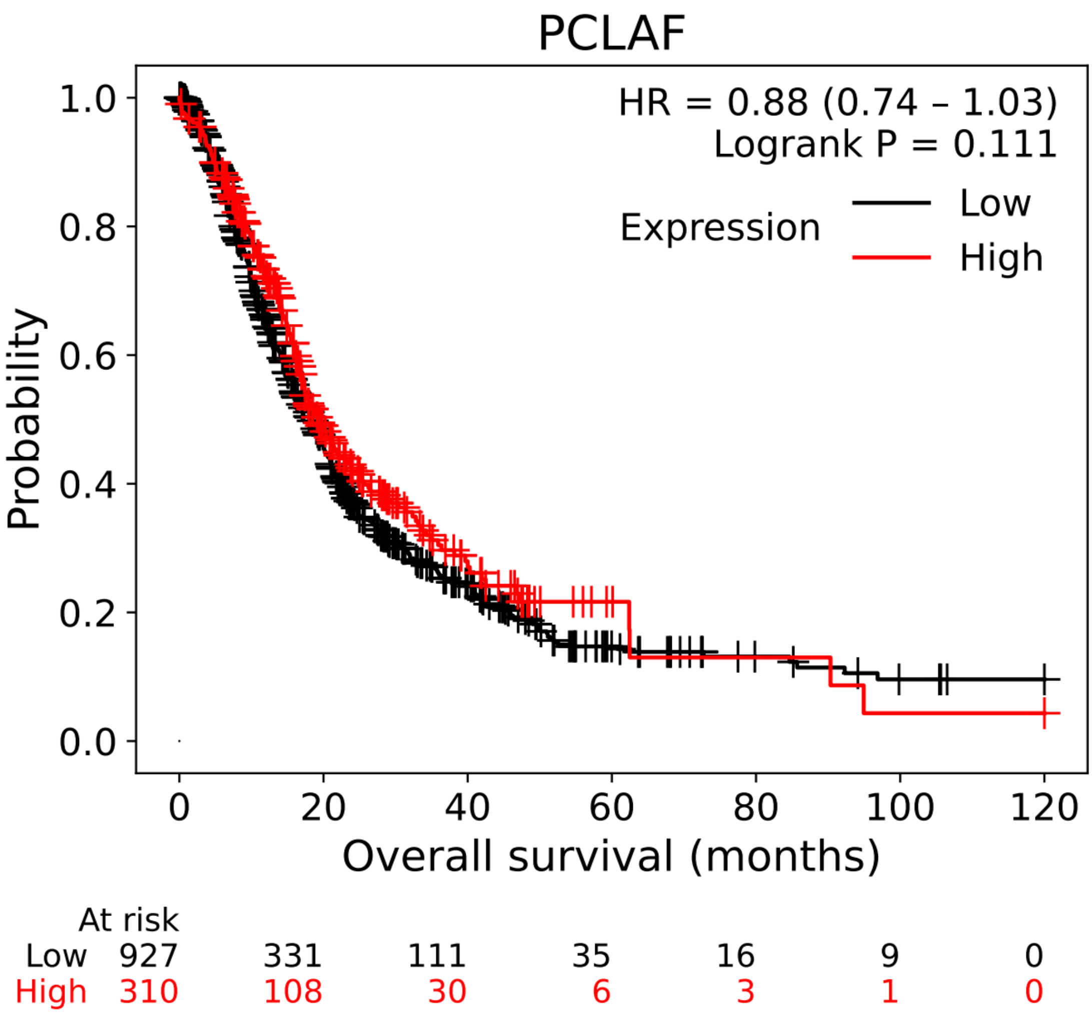

Figure S 32: Survival analysis for PCLAF, which is an MI gene in the comparison between TGFB1 stimulated cells and TGFB1+GEM-treated cells. HR > 1: High expression associated with worse overall survival (OS), HR < 1: High expression associated with better OS, Log-rank P < 0.05 considered statistically significant.

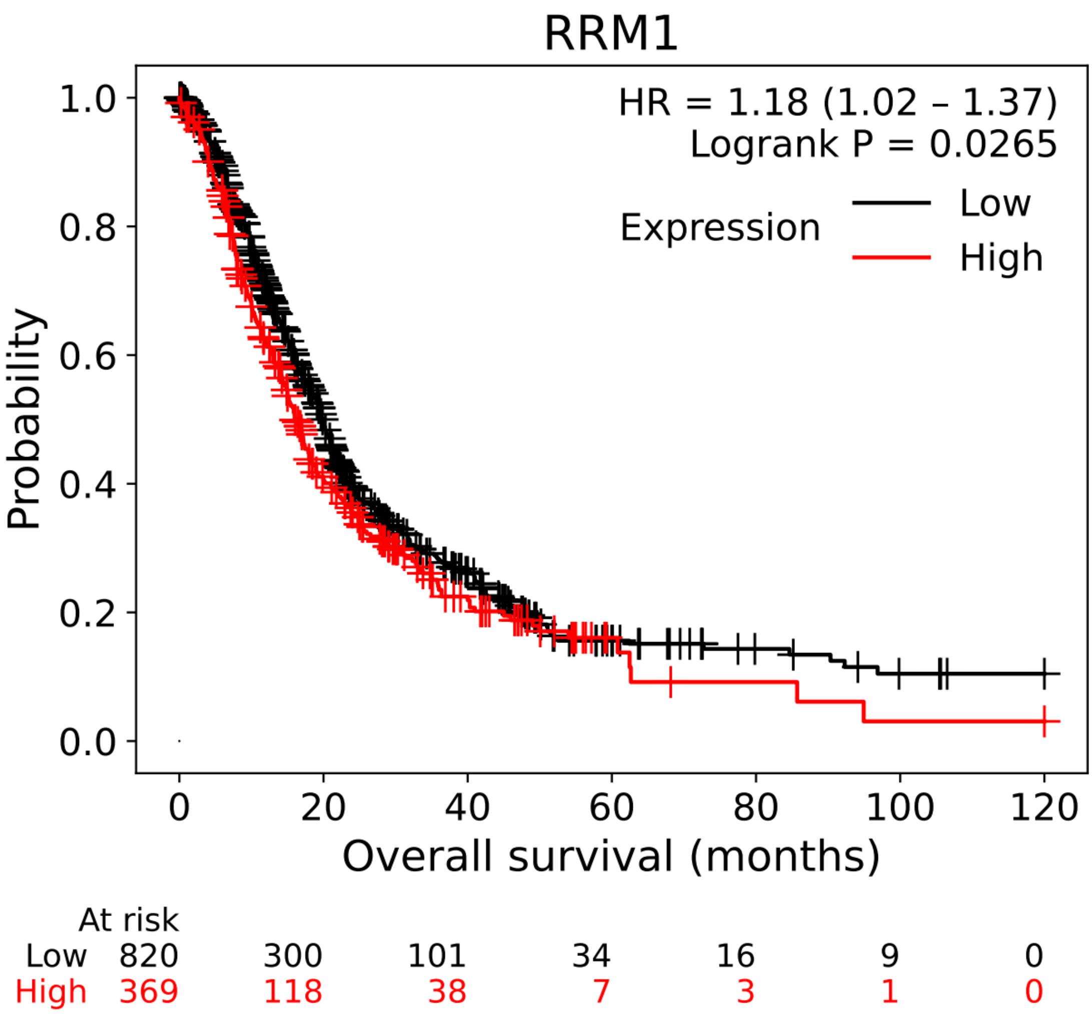

Figure S 33: Survival analysis for RRM1, which is an MI gene in the comparison between TGFB1 stimulated cells and TGFB1+GEM-treated cells. HR > 1: High expression associated with worse overall survival (OS), HR < 1: High expression associated with better OS, Log-rank P < 0.05 considered statistically significant.

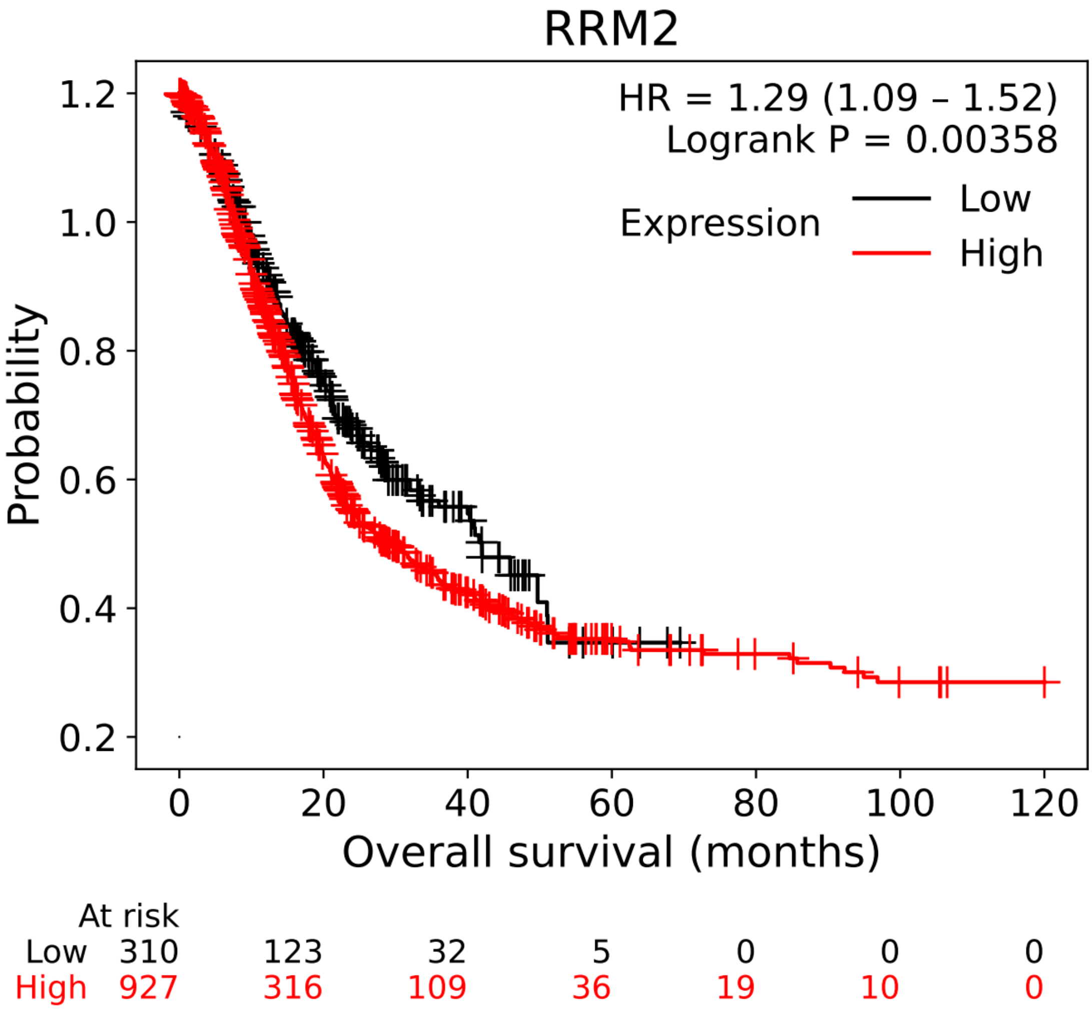

Figure S 34: Survival analysis for RRM2, which is an MI gene in the comparison between TGFB1 stimulated cells and TGFB1+GEM-treated cells. HR > 1: High expression associated with worse overall survival (OS), HR < 1: High expression associated with better OS, Log-rank P < 0.05 considered statistically significant.

Survival analyses of the ten top-ranked mutual information (MI) genes for the comparison between predicted-sensitive control cells and GEM-treated cells

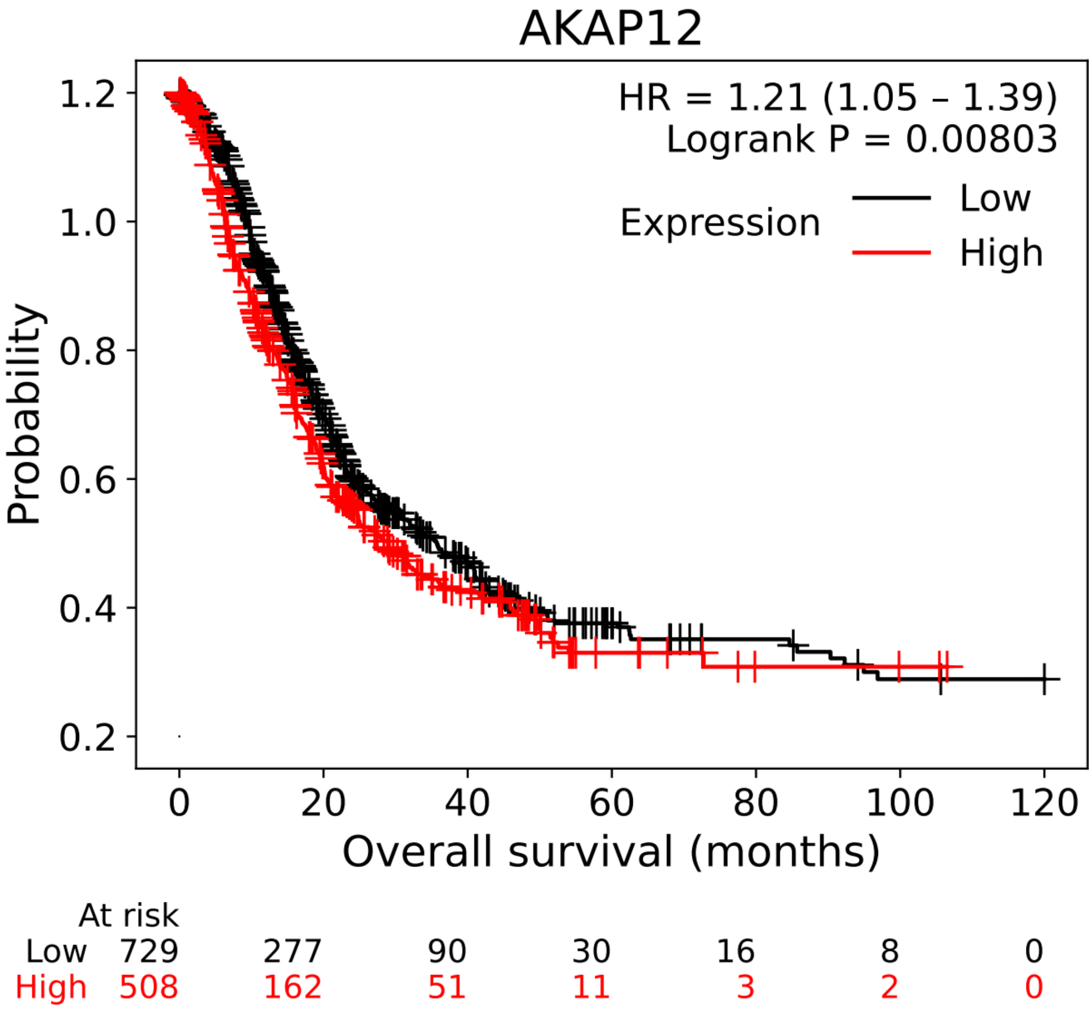

Figure S 35: Survival analysis for AKAP12, which is an MI gene in the comparison between predicted-sensitive control cells and GEM-treated cells. HR > 1: High expression associated with worse overall survival (OS), HR < 1: High expression associated with better OS, Log-rank P < 0.05 considered statistically significant.

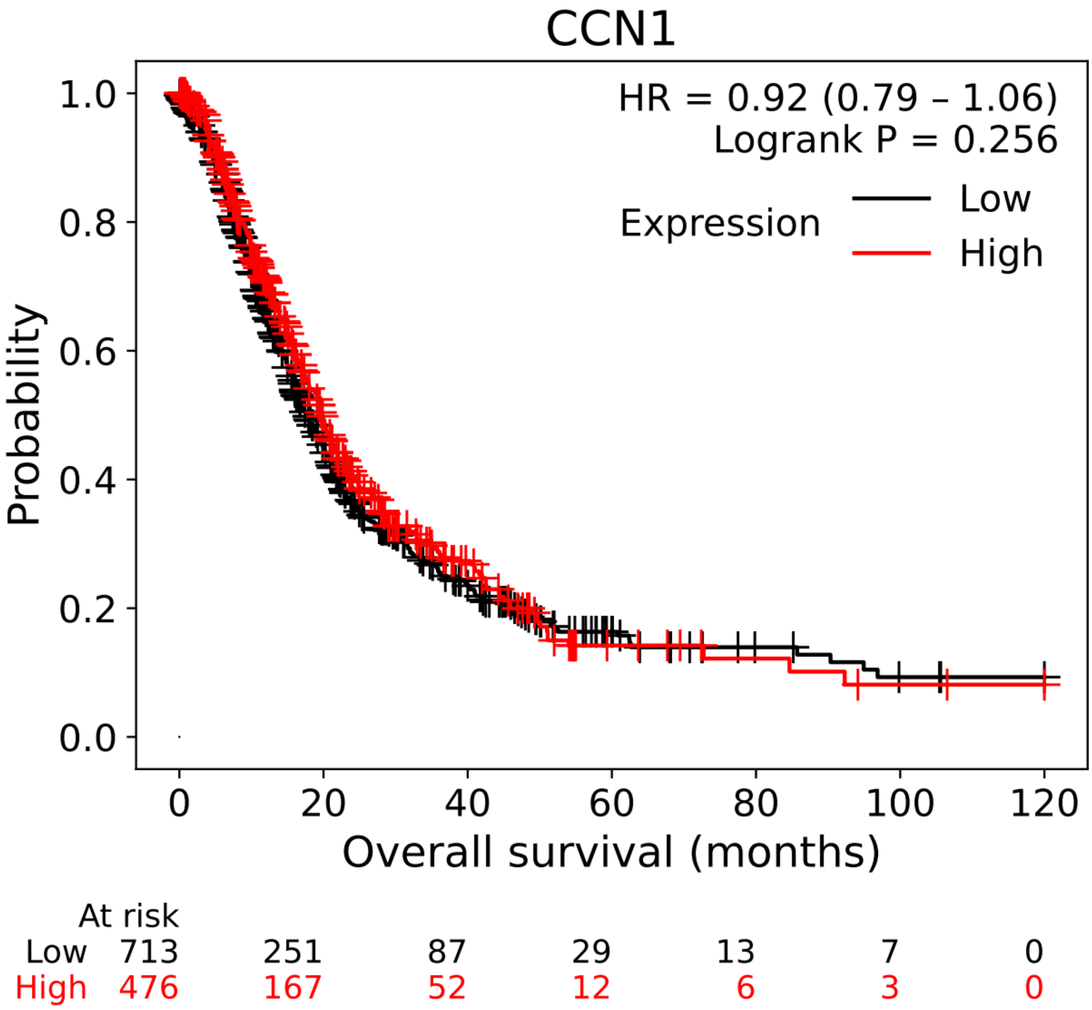

Figure S 36: Survival analysis for CCN1, which is an MI gene in the comparison between predicted-sensitive control cells and GEM-treated cells. HR > 1: High expression associated with worse overall survival (OS), HR < 1: High expression associated with better OS, Log-rank P < 0.05 considered statistically significant.

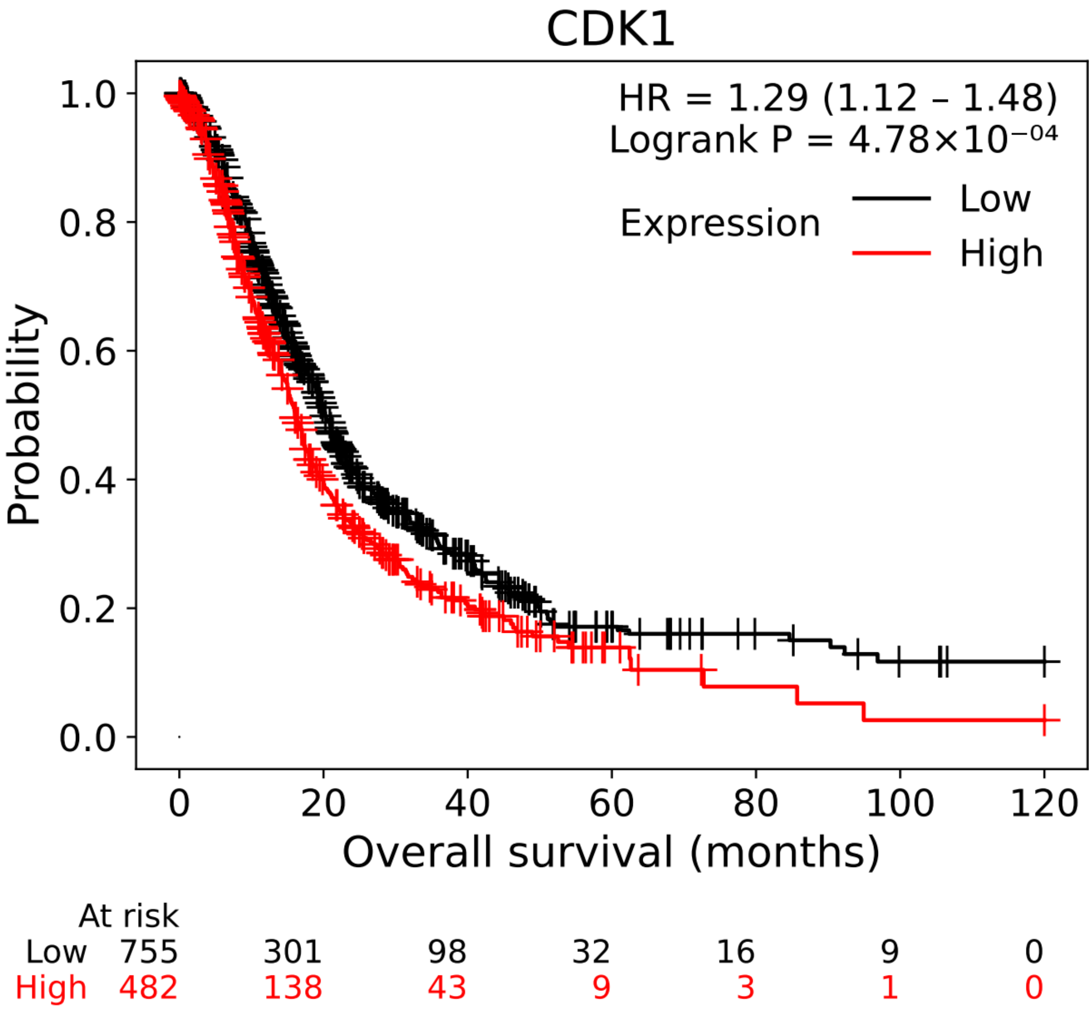

Figure S 37: Survival analysis for CDK1, which is an MI gene in the comparison between predicted-sensitive control cells and GEM-treated cells. HR > 1: High expression associated with worse overall survival (OS), HR < 1: High expression associated with better OS, Log-rank P < 0.05 considered statistically significant.

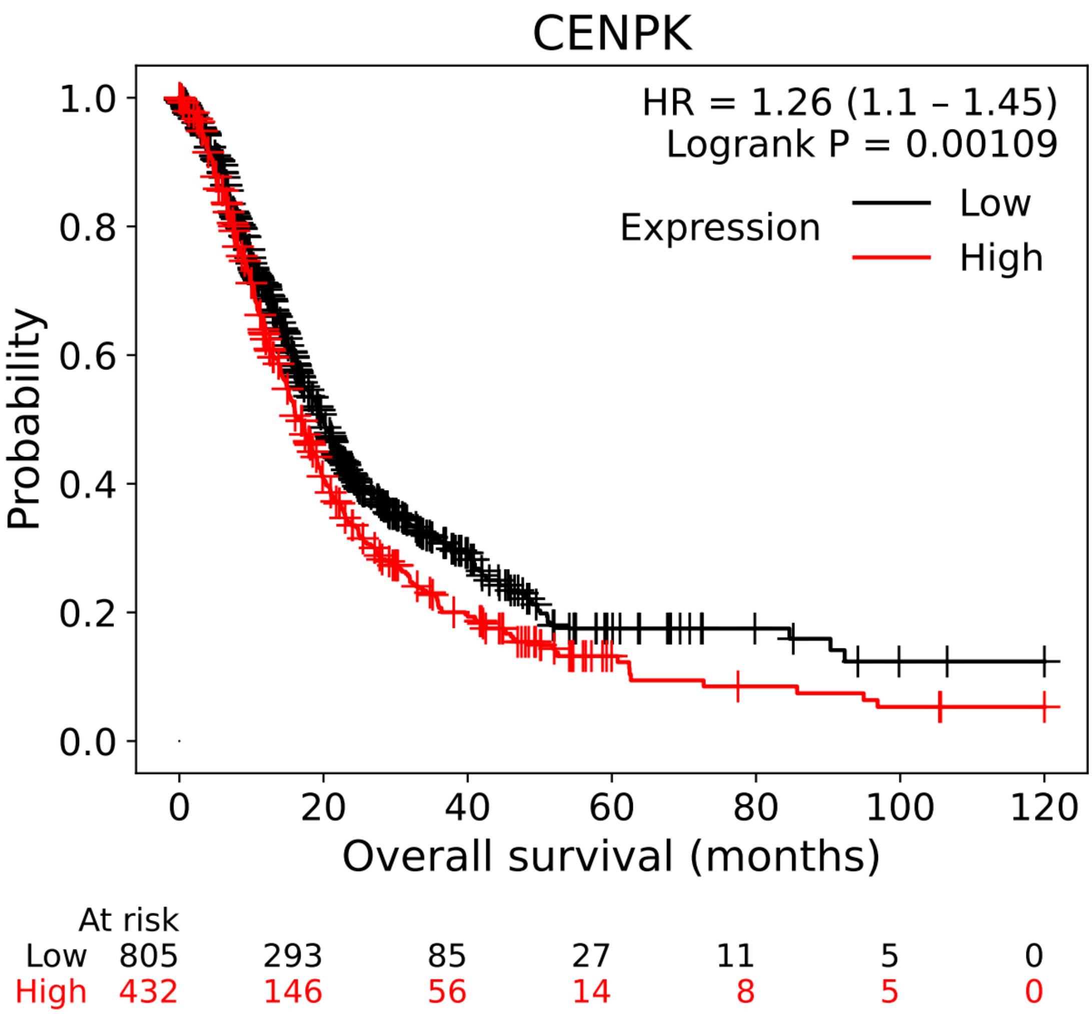

Figure S 38: Survival analysis for CENPK, which is an MI gene in the comparison between predicted-sensitive control cells and GEM-treated cells. HR > 1: High expression associated with worse overall survival (OS), HR < 1: High expression associated with better OS, Log-rank P < 0.05 considered statistically significant.

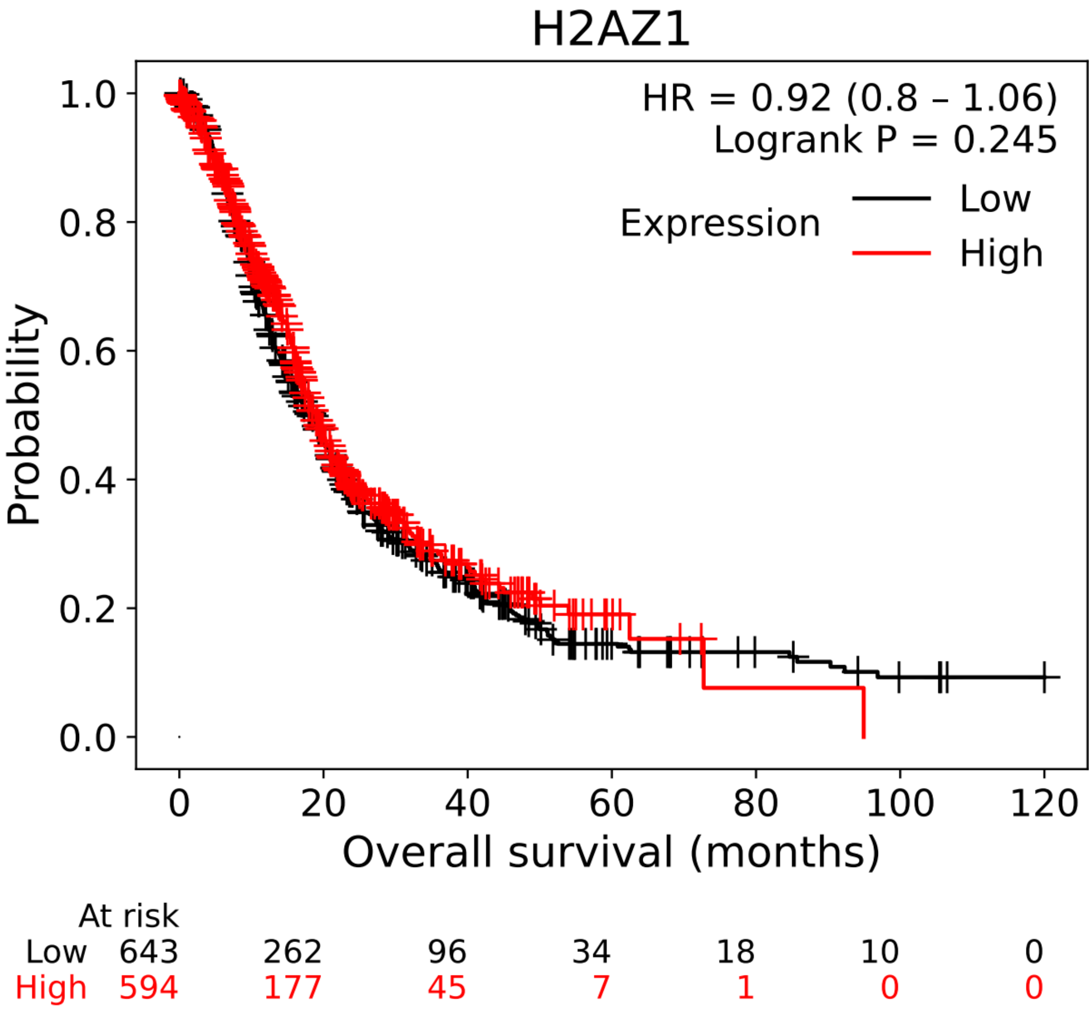

Figure S 39: Survival analysis for H2AZ1, which is an MI gene in the comparison between predicted-sensitive control cells and GEM-treated cells. HR > 1: High expression associated with worse overall survival (OS), HR < 1: High expression associated with better OS, Log-rank P < 0.05 considered statistically significant.

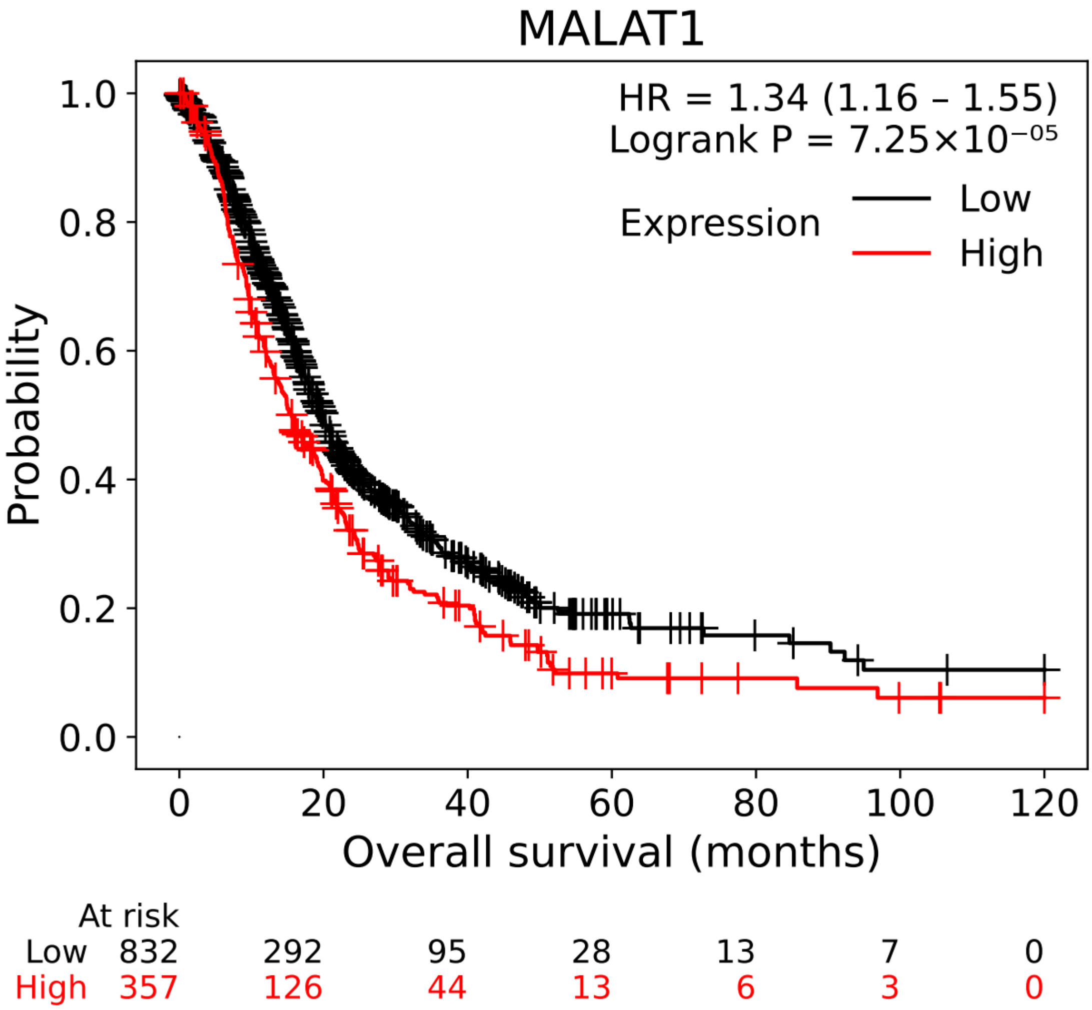

Figure S 40: Survival analysis for MALAT1, which is an MI gene in the comparison between predicted-sensitive control cells and GEM-treated cells. HR > 1: High expression associated with worse overall survival (OS), HR < 1: High expression associated with better OS, Log-rank P < 0.05 considered statistically significant.

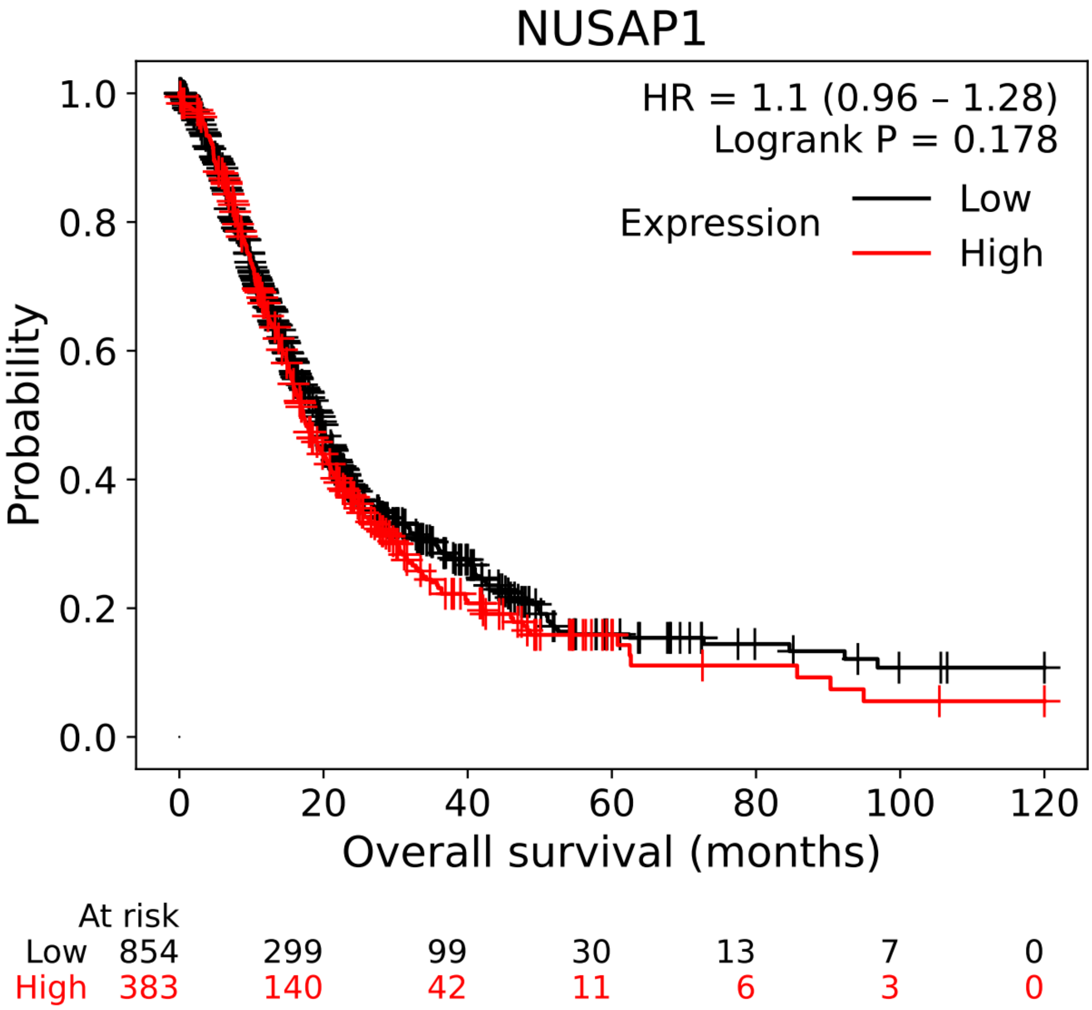

Figure S 41: Survival analysis for NUSAP1, which is an MI gene in the comparison between predicted-sensitive control cells and GEM-treated cells. HR > 1: High expression associated with worse overall survival (OS), HR < 1: High expression associated with better OS, Log-rank P < 0.05 considered statistically significant.

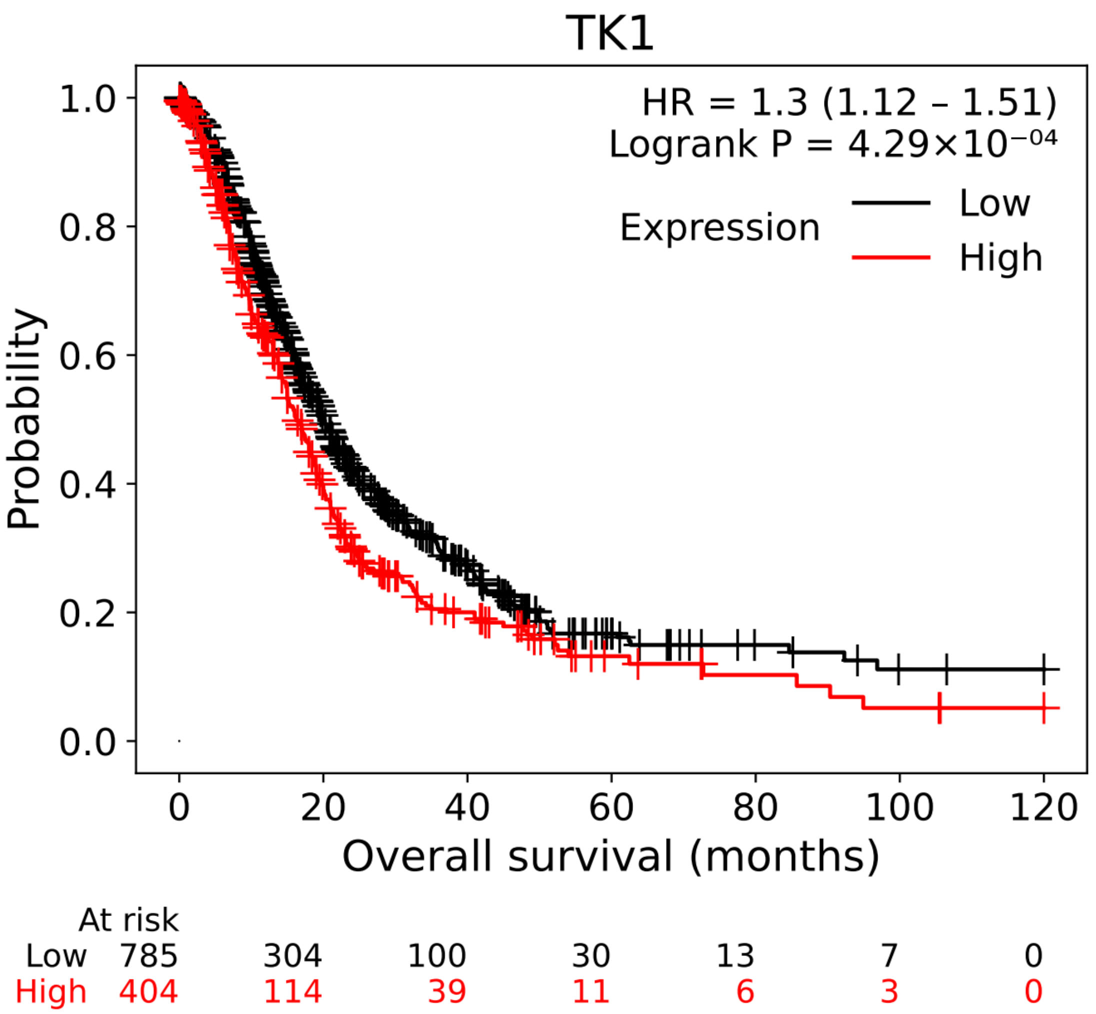

Figure S 42: Survival analysis for TK1, which is an MI gene in the comparison between predicted-sensitive control cells and GEM-treated cells. HR > 1: High expression associated with worse overall survival (OS), HR < 1: High expression associated with better OS, Log-rank P < 0.05 considered statistically significant.

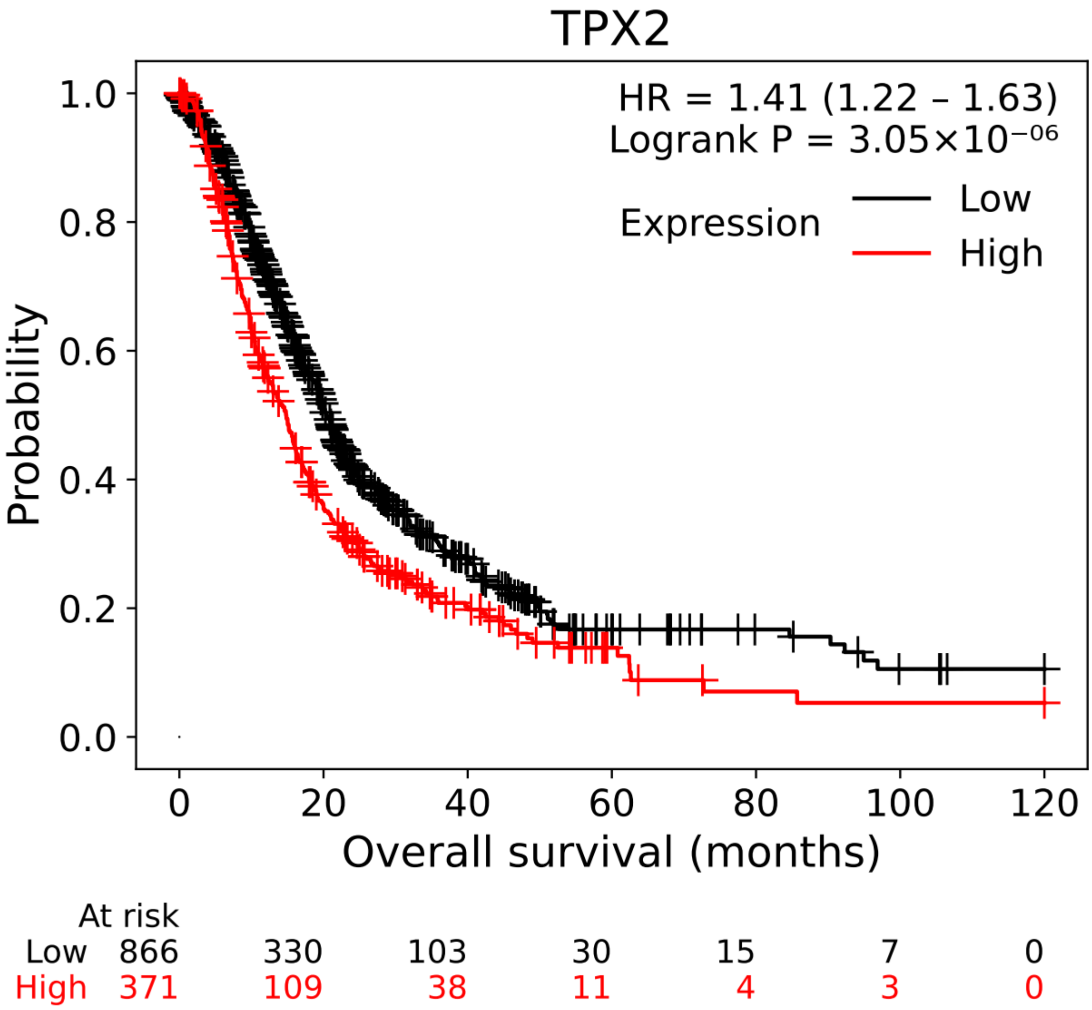

Figure S 43: Survival analysis for TPX2, which is an MI gene in the comparison between predicted-sensitive control cells and GEM-treated cells. HR > 1: High expression associated with worse overall survival (OS), HR < 1: High expression associated with better OS, Log-rank P < 0.05 considered statistically significant.

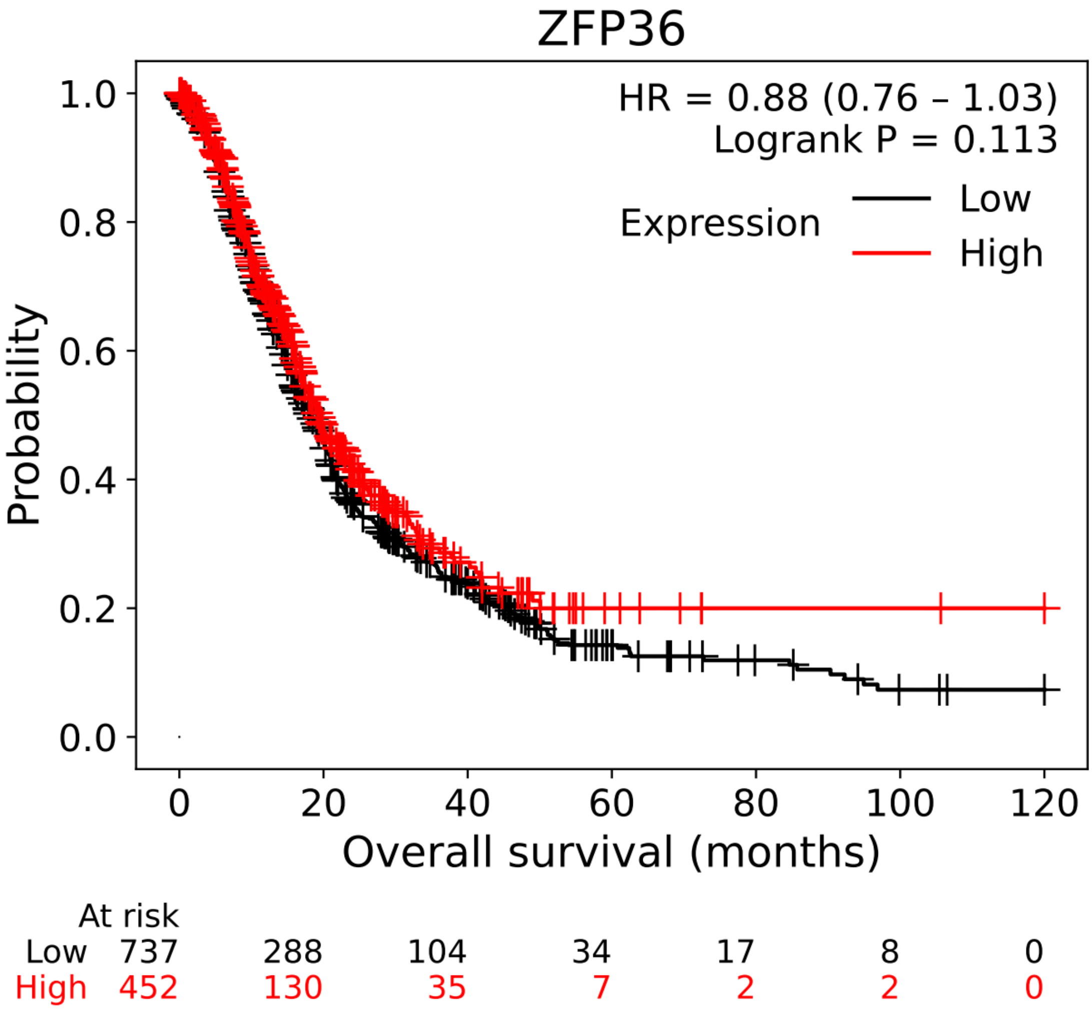

Figure S 44: Survival analysis for ZFP36, which is an MI gene in the comparison between predicted-sensitive control cells and GEM-treated cells. HR > 1: High expression associated with worse overall survival (OS), HR < 1: High expression associated with better OS, Log-rank P < 0.05 considered statistically significant.

Limitations and Conclusion

Gene-level inspection based on mutual information ranking identified a set of differentially expressed genes between predicted-sensitive and GEM-treated cells. While several of these genes displayed distinct differences in gene expression when visualized as violin plots, their biological relevance with respect to known GEM-response mechanisms remains uncertain.

Among the tested methods, the PCA-cosine (min) strategy produced the most interpretable gene-level profiles with several top-ranked genes showing expression trends broadly consistent with expected treatment effects according to the literature.

Although the PCA-cosine (mean) strategy achieved the highest classification accuracy across stratification thresholds, the gene-level separation observed with the PCA-cosine (min) method appeared more biologically interpretable and aligned better with known response-associated genes. This highlights a potential trade-off between statistical performance and biological interpretability that warrants further investigation. While most of the significantly differentially expressed genes identified using the PCA-cosine (mean) strategy were also identified using the PCA-cosine (min) strategy, *SBF2* was only among the top-ranked MI genes for the PCA-cosine (mean) strategy. As variants in the *SBF2* gene have been associated with survival in pancreatic adenocarcinoma [82], and knock-down of the long non-coding RNA SBF2 Antisense RNA 1 (SBF2-AS1) has been shown to promote apoptosis in GEM-resistant pancreatic cancer cells [83], further research regarding the role of *SBF2* in pancreatic cancer might be warranted.

Importantly, the proposed stratification is not intended as a predictive model for treatment outcomes, but rather as a hypothesis-generating framework to explore possible latent transcriptional heterogeneity in the untreated state.

However, the robustness and biological relevance of the resulting classifications remain to be validated. Future work should incorporate orthogonal data types such as proteomic or epigenomic information, evaluate dynamic responses over time, and assess whether the proposed framework can be applied to other drugs or other cell types. Experimental validation will be essential to assess whether the identified transcriptional states correspond to meaningful phenotypic outcomes.

Taken together, our retrospective similarity-based stratification offers a transparent and principled approach for exploring latent transcriptional heterogeneity prior to treatment. While not intended as a predictive model, it may serve as a useful entry point for identifying transcriptionally distinct cell states or gene expression patterns that might be linked to therapeutic susceptibility. As an initial step in this direction, the approach highlights how

transcriptional dissimilarity, e.g., between the predicted-sensitive cells identified using our retrospective similarity-based stratification approach and GEM-treated cells, might relate to latent sensitivity and offers a starting point for further hypothesis generation.

References

1. Song, M., et al., *PMEPA1 Stimulates the Proliferation, Colony Formation of Pancreatic Cancer Cells via the MAPK Signaling Pathway*. The American Journal of the Medical Sciences, 2021. **362**(3): p. 291-296.
2. Yang, Y., et al., *PMEPA1 interference activates PTEN/PI3K/AKT, thereby inhibiting the proliferation, invasion and migration of pancreatic cancer cells and enhancing the sensitivity to gemcitabine and cisplatin*. Drug Development Research, 2022. **83**(1): p. 64-74.
3. Porter, R.L., et al., *Epithelial to mesenchymal plasticity and differential response to therapies in pancreatic ductal adenocarcinoma*. Proceedings of the National Academy of Sciences, 2019. **116**(52): p. 26835-26845.
4. Ungefroren, H., et al. *The Quasimesenchymal Pancreatic Ductal Epithelial Cell Line PANC-1—A Useful Model to Study Clonal Heterogeneity and EMT Subtype Shifting*. Cancers, 2022. **14**, DOI: 10.3390/cancers14092057.
5. Liu, W.-J., et al., *Plasminogen Activator Inhibitor 1 as a Poor Prognostic Indicator in Resectable Pancreatic Ductal Adenocarcinoma*. Chinese Medical Journal, 2018. **131**(24).
6. Klimczak-Bitner, A.A., et al., *Expression of MMP9, SERPINE1 and miR-134 as prognostic factors in esophageal cancer*. Oncol Lett, 2016. **12**(5): p. 4133-4138.
7. Wong, S.Q.R., et al., *Modeling oxaliplatin resistance in colorectal cancer reveals a SERPINE1-based gene signature (RESIST-M) and therapeutic strategies for pro-metastatic CMS4 subtype*. Cell Death & Disease, 2025. **16**(1): p. 529.
8. Hongu, T., et al., *Permeable Lung Vasculature Creates Chemoresistant Endothelial Niche by Producing SERPINE1 at Breast Cancer Metastatic Sites*. Cancer Science, 2025. **116**(6): p. 1604-1615.
9. Su, Y.-H., et al., *Obesity promotes radioresistance through SERPINE1-mediated aggressiveness and DNA repair of triple-negative breast cancer*. Cell Death & Disease, 2023. **14**(1): p. 53.
10. Zhang, Z., et al., *TGFBI promotes EMT and perineural invasion of pancreatic cancer via PI3K/AKT pathway*. Medical Oncology, 2025. **42**(6): p. 181.
11. Zhou, J., et al., *A novel role of TGFBI in macrophage polarization and macrophage-induced pancreatic cancer growth and therapeutic resistance*. Cancer Letters, 2023. **578**: p. 216457.
12. Sato, T., et al., *Identification and characterization of transforming growth factor beta-induced in circulating tumor cell subline from pancreatic cancer cell line*. Cancer Science, 2018. **109**(11): p. 3623-3633.
13. Safina, A.F., et al., *Ras alters epithelial-mesenchymal transition in response to TGF-β by reducing actin fibers and cell-matrix adhesion*. Cell Cycle, 2009. **8**(2): p. 284-298.
14. Luan, H., et al., *The identification of liver metastasis- and prognosis-associated genes in pancreatic ductal adenocarcinoma*. BMC Cancer, 2022. **22**(1): p. 463.
15. Pan, H., et al., *Tropomyosin-1 acts as a potential tumor suppressor in human oral squamous cell carcinoma*. PLOS ONE, 2017. **12**(2): p. e0168900.
16. Li, Y., et al., *Co-delivery of microRNA-21 antisense oligonucleotides and gemcitabine using nanomedicine for pancreatic cancer therapy*. Cancer Science, 2017. **108**(7): p. 1493-1503.
17. Sun, T., et al., *Aberrant MicroRNAs in Pancreatic Cancer: Researches and Clinical Implications*. Gastroenterology Research and Practice, 2014. **2014**(1): p. 386561.
18. Ali, S., et al., *Differentially expressed miRNAs in the plasma may provide a molecular signature for aggressive pancreatic cancer*. Am J Transl Res, 2010. **3**(1): p. 28-47.

19. Miao, L., et al., *SPOCK1 is a novel transforming growth factor-β target gene that regulates lung cancer cell epithelial-mesenchymal transition*. Biochemical and Biophysical Research Communications, 2013. **440**(4): p. 792-797.
20. Cui, X., et al., *SPOCK1 promotes metastasis in pancreatic cancer via NF-κB-dependent epithelial-mesenchymal transition by interacting with IκB-α*. Cellular Oncology, 2022. **45**(1): p. 69-84.
21. Veenstra, V.L., et al., *Stromal SPOCK1 supports invasive pancreatic cancer growth*. Molecular Oncology, 2017. **11**(8): p. 1050-1064.
22. Li, J., et al., *A potential prognostic marker and therapeutic target: SPOCK1 promotes the proliferation, metastasis, and apoptosis of pancreatic ductal adenocarcinoma cells*. Journal of Cellular Biochemistry, 2020. **121**(1): p. 743-754.
23. Li, X., et al., *COL1A1: A novel oncogenic gene and therapeutic target in malignancies*. Pathology - Research and Practice, 2022. **236**: p. 154013.
24. Chen, Y., et al., *Oncogenic collagen I homotrimers from cancer cells bind to α3β1 integrin and impact tumor microbiome and immunity to promote pancreatic cancer*. Cancer Cell, 2022. **40**(8): p. 818-834.e9.
25. Li, Q., et al., *The type I collagen paradox in PDAC progression: microenvironmental protector turned tumor accomplice*. Journal of Translational Medicine, 2025. **23**(1): p. 744.
26. Tian, C., et al., *Suppression of pancreatic ductal adenocarcinoma growth and metastasis by fibrillar collagens produced selectively by tumor cells*. Nature Communications, 2021. **12**(1): p. 2328.
27. Chakravarthy, D., et al., *Palmatine suppresses glutamine-mediated interaction between pancreatic cancer and stellate cells through simultaneous inhibition of survivin and COL1A1*. Cancer Letters, 2018. **419**: p. 103-115.
28. Jensen, L.J., et al., *STRING 8—a global view on proteins and their functional interactions in 630 organisms*. Nucleic Acids Research, 2009. **37**(suppl_1): p. D412-D416.
29. Szklarczyk, D., et al., *STRING v11: protein–protein association networks with increased coverage, supporting functional discovery in genome-wide experimental datasets*. Nucleic Acids Research, 2019. **47**(D1): p. D607-D613.
30. Szklarczyk, D., et al., *The STRING database in 2023: protein–protein association networks and functional enrichment analyses for any sequenced genome of interest*. Nucleic Acids Research, 2023. **51**(D1): p. D638-D646.
31. Zhang, J., et al., *Single-cell analysis reveals the COL11A1+ fibroblasts are cancer-specific fibroblasts that promote tumor progression*. Frontiers in Pharmacology, 2023. **Volume 14 - 2023**.
32. Fortier, A.-M., E. Asselin, and M. Cadrin, *Keratin 8 and 18 Loss in Epithelial Cancer Cells Increases Collective Cell Migration and Cisplatin Sensitivity through Claudin1 Up-regulation **. Journal of Biological Chemistry, 2013. **288**(16): p. 11555-11571.
33. Xiong, F., et al., *Keratin 8 Is an Inflammation-Induced and Prognosis-Related Marker for Pancreatic Adenocarcinoma*. Disease Markers, 2022. **2022**(1): p. 8159537.
34. Kim, Y., et al., *Comparative Proteomic Profiling of Pancreatic Ductal Adenocarcinoma Cell Lines*. Molecules and Cells, 2014. **37**(12): p. 888-898.
35. Cook, D.P. and B.C. Vanderhyden, *Context specificity of the EMT transcriptional response*. Nature Communications, 2020. **11**(1): p. 2142.
36. Sunami, Y., et al. *Single Cell Analysis of Cultivated Fibroblasts from Chronic Pancreatitis and Pancreatic Cancer Patients*. Cells, 2022. **11**, DOI: 10.3390/cells11162583.
37. Walsh, N., et al., *Identification of pancreatic cancer invasion-related proteins by proteomic analysis*. Proteome Science, 2009. **7**(1): p. 3.
38. Ávila-López, P.A., et al., *H2A.Z overexpression suppresses senescence and chemosensitivity in pancreatic ductal adenocarcinoma*. Oncogene, 2021. **40**(11): p. 2065-2080.

39. Giaimo, B.D., et al., *The histone variant H2A.Z in gene regulation.* Epigenetics & Chromatin, 2019. **12**(1): p. 37.
40. Dong, M., et al., *H2AFZ Is a Prognostic Biomarker Correlated to TP53 Mutation and Immune Infiltration in Hepatocellular Carcinoma.* Frontiers in Oncology, 2021. **Volume 11 - 2021**.
41. Diegmüller, F., J. Leers, and S.B. Hake, *The "Ins and Outs and What-Abouts" of H2A.Z: A tribute to C. David Allis.* Journal of Biological Chemistry, 2025. **301**(2): p. 108154.
42. Mardin, W.A., et al., *SERPINB5 and AKAP12-- Expression and promoter methylation of metastasis suppressor genes in pancreatic ductal adenocarcinoma.* BMC Cancer, 2010. **10**(1): p. 549.
43. Liang, Q., et al., *Pan-cancer analysis of the prognosis and immunological role of AKAP12: A potential biomarker for resistance to anti-VEGF inhibitors.* Frontiers in Genetics, 2022. **Volume 13 - 2022**.
44. Wu, X., et al., *The Mechanism and Influence of AKAP12 in Different Cancers.* Biomedical and Environmental Sciences, 2018. **31**(12): p. 927.
45. Wijnen, R., et al., *Cyclin Dependent Kinase-1 (CDK-1) Inhibition as a Novel Therapeutic Strategy against Pancreatic Ductal Adenocarcinoma (PDAC).* Cancers, 2021. **13**(17): p. 4389.
46. Jiang, P., et al., *Expression patterns and prognostic values of the cyclin-dependent kinase 1 and cyclin A2 gene cluster in pancreatic adenocarcinoma.* Journal of International Medical Research, 2020. **48**(12): p. 0300060520930113.
47. Xu, X., et al., *Post-translational modification of CDK1–STAT3 signaling by fisetin suppresses pancreatic cancer stem cell properties.* Cell & Bioscience, 2023. **13**(1): p. 176.
48. Li, L., et al., *Long Noncoding RNA MALAT1 Promotes Aggressive Pancreatic Cancer Proliferation and Metastasis via the Stimulation of Autophagy.* Molecular Cancer Therapeutics, 2016. **15**(9): p. 2232-2243.
49. Jiao, F., et al., *Elevated expression level of long noncoding RNA MALAT-1 facilitates cell growth, migration and invasion in pancreatic cancer.* Oncol Rep, 2014. **32**(6): p. 2485-2492.
50. Chen, W., et al., *MALAT1 enhances gemcitabine resistance in non-small cell lung cancer cells by directly affecting miR-27a-5p/PBOV1 axis.* Cellular Signalling, 2022. **94**: p. 110326.
51. Wei, L., et al., *Noncoding RNAs: an emerging modulator of drug resistance in pancreatic cancer.* Frontiers in Cell and Developmental Biology, 2023. **Volume 11 - 2023**.
52. Kim, N.Y., et al., *Cannabidiol Suppresses EMT in Pancreatic Cancer via Inhibition of MALAT1 lncRNA and PI3K/Akt/mTOR Signaling Pathway.* IUBMB Life, 2025. **77**(8): p. e70042.
53. Pang, E.-J., et al., *Overexpression of long non-coding RNA MALAT1 is correlated with clinical progression and unfavorable prognosis in pancreatic cancer.* Tumor Biology, 2015. **36**(4): p. 2403-2407.
54. Liu, P., et al., *The lncRNA MALAT1 acts as a competing endogenous RNA to regulate KRAS expression by sponging miR-217 in pancreatic ductal adenocarcinoma.* Scientific Reports, 2017. **7**(1): p. 5186.
55. Haque, I., et al., *The Matricellular Protein CCN1/Cyr61 Is a Critical Regulator of Sonic Hedgehog in Pancreatic Carcinogenesis* *. Journal of Biological Chemistry, 2012. **287**(46): p. 38569-38579.
56. Kim, H., S. Son, and I. Shin, *Role of the CCN protein family in cancer.* BMB Reports, 2018. **51**(10): p. 486-492.
57. Gündel, B., et al., *The Crosstalk Analysis between mPSCs and Panc1 Cells Identifies CCN1 as a Positive Regulator of Gemcitabine Sensitivity in Pancreatic Cancer Cells.* International Journal of Molecular Sciences, 2024. **25**(17): p. 9369.

58. Zeng, H., et al., *Pan-cancer investigation of CENPK gene: clinical significance and oncogenic immunology.* Am J Transl Res, 2021. **13**(12): p. 13336-13355.
59. Chen, X., et al., *The cell cycle gene centromere protein K (CENPK) contributes to the malignant progression and prognosis of prostate cancer.* Translational Cancer Research, 2022. **11**(5): p. 1099-1111.
60. Wang, J., et al., *Downregulation of CENPK suppresses hepatocellular carcinoma malignant progression through regulating YAP1.* OncoTargets and Therapy, 2019. **12**: p. 869-882.
61. Huang, M., et al., *The Role of CENPK Splice Variant in Abiraterone Response in Metastatic Castration-Resistant Prostate Cancer.* Cells, 2024. **13**(19): p. 1622.
62. Huggett, M.T., et al., *Phase I/II study of verteporfin photodynamic therapy in locally advanced pancreatic cancer.* British Journal of Cancer, 2014. **110**(7): p. 1698-1704.
63. Zheng, H., et al., *Comprehensive pan-cancer analysis reveals NUSAP1 is a novel predictive biomarker for prognosis and immunotherapy response.* International Journal of Biological Sciences, 2023. **19**(14): p. 4689-4708.
64. Liu, Y., et al., *NUSAP1 promotes pancreatic ductal adenocarcinoma progression by drives the epithelial-mesenchymal transition and reduces AMPK phosphorylation.* BMC Cancer, 2024. **24**(1): p. 87.
65. Zhang, T., et al., *ZFP36 loss-mediated BARX1 stabilization promotes malignant phenotypes by transactivating master oncogenes in NSCLC.* Cell Death & Disease, 2023. **14**(8): p. 527.
66. Sun, X.-J., et al., *MicroRNA-29a Promotes Pancreatic Cancer Growth by Inhibiting Tristetraprolin.* Cellular Physiology and Biochemistry, 2015. **37**(2): p. 707-718.
67. Wei, Z.-R., et al., *Low tristetraprolin expression promotes cell proliferation and predicts poor patients outcome in pancreatic cancer.* Oncotarget, 2016. **7**(14).
68. Zhu, X., et al., *Thymidine kinase 1 silencing retards proliferative activity of pancreatic cancer cell via E2F1-TK1-P21 axis.* Cell Proliferation, 2018. **51**(3): p. e12428.
69. Nisman, B., et al., *Serum Thymidine Kinase 1 Activity in the Prognosis and Monitoring of Chemotherapy in Lung Cancer Patients: A Brief Report.* Journal of Thoracic Oncology, 2014. **9**(10): p. 1568-1572.
70. Warner, S.L., et al., *Validation of TPX2 as a Potential Therapeutic Target in Pancreatic Cancer Cells.* Clinical Cancer Research, 2009. **15**(21): p. 6519-6528.
71. Guenther, M., et al., *TPX2 expression as a negative predictor of gemcitabine efficacy in pancreatic cancer.* British Journal of Cancer, 2023. **129**(1): p. 175-182.
72. Jolliffe, I.T. and J. Cadima, *Principal component analysis: a review and recent developments.* Philosophical Transactions of the Royal Society A: Mathematical, Physical and Engineering Sciences, 2016. **374**(2065): p. 20150202.
73. Jolliffe, I.T., *Introduction*, in *Principal Component Analysis*, I.T. Jolliffe, Editor. 2002, Springer New York: New York, NY. p. 1-9.
74. van der Maaten, L. and G. Hinton, *Visualizing Data using t-SNE.* Journal of Machine Learning Research, 2008. **9**(86): p. 2579--2605.
75. Kobak, D. and P. Berens, *The art of using t-SNE for single-cell transcriptomics.* Nature Communications, 2019. **10**(1): p. 5416.
76. Kirişci, M., *New cosine similarity and distance measures for Fermatean fuzzy sets and TOPSIS approach.* Knowledge and Information Systems, 2023. **65**(2): p. 855-868.
77. Tjaden, B., *An approach for clustering gene expression data with error information.* BMC Bioinformatics, 2006. **7**(1): p. 17.
78. Tsuyuzaki, K., et al., *Benchmarking principal component analysis for large-scale single-cell RNA-sequencing.* Genome Biology, 2020. **21**(1): p. 9.
79. Caliskan, D., et al., *gSELECT: A novel pre-analysis machine-learning library enabling early hypothesis testing and predictive gene selection in single-cell data.* Computational and Structural Biotechnology Journal, 2025. **27**: p. 3510-3527.

80. Caliskan, A., et al., *Optimized cell type signatures revealed from single-cell data by combining principal feature analysis, mutual information, and machine learning*. Computational and Structural Biotechnology Journal, 2023. **21**: p. 3293-3314.
81. Rasbach, L., et al., *An orchestra of machine learning methods reveals landmarks in single-cell data exemplified with aging fibroblasts*. PLOS ONE, 2024. **19**(4): p. e0302045.
82. Franks, I., *Variants in SBF2 gene associated with survival in pancreatic adenocarcinoma*. Nature Reviews Gastroenterology & Hepatology, 2013. **10**(1): p. 4-4.
83. Hua, Y.-Q., et al., *Long non-coding SBF2-AS1 acting as a competing endogenous RNA to sponge microRNA-142-3p to participate in gemcitabine resistance in pancreatic cancer via upregulating TWF1*. Aging (Albany NY), 2019. **11**(20): p. 8860-8878.
